# *Hymenoscyphus fraxineus* vs. *Hymenoscyphus albidus* – A comparative light microscopic study on the causal agent of European ash dieback and related foliicolous, stroma-forming species

**DOI:** 10.1080/21501203.2014.963720

**Published:** 2014-10-14

**Authors:** Hans-Otto Baral, Martin Bemmann

**Affiliations:** ^a^ Blaihofstraße 42, D-72074Tübingen, Germany; ^b^ Kleingemünderstraße 111, D-69118Heidelberg, Germany

**Keywords:** *Hymenoscyphus aesculi* comb. nov. (for *Helotium*/*Lanzia aesculi)*, *Hy. honshuanus* nom. nov. (for *Lambertellinia scutuloides)*, *Hy. torquatus* comb. nov. (for *Lambertella torquata*), croziers, simple septa, homothallism, pseudosclerotium, *Fraxinus*, *Aesculus*, *Acer*, Helotiaceae, Rutstroemiaceae, invasive species, morphology, molecular markers

## Abstract

Five species of *Hymenoscyphus* that fruit on black stromatized parts of dead leaves of deciduous trees are presented, giving details on their morphological and ecological characteristics. Several of these species have previously been misplaced in rutstroemiaceous genera because of the presence of a substratal stroma. However, the heteropolar, scutuloid ascospores with an often hook-like lateral protrusion at the rounded apex and the ascus apical ring of the *Hymenoscyphus*-type represent two reliable morphological characteristics that, together with molecular data, provide clear evidence for their placement in the genus *Hymenoscyphus* (Helotiaceae). Among the species treated is *Hymenoscyphus fraxineus* (=*Hymenoscyphus pseudoalbidus*), the causal agent of the European ash dieback disease. Since 1992 this species started within Europe to replace the rather uncommon *Hymenoscyphus albidus*, which is likewise confined to leaves of *Fraxinus. Hy. fraxineus* has been recorded already since 1990 in Eastern Asia (Japan, Korea, northeast of China), where it had been initially misidentified as *Lambertella albida* (≡*Hy. albidus*). In these regions, it occurs as a harmless saprotroph on *Fraxinus mandshurica* and *Fraxinus rhynchophylla*, suggesting that those populations are native while the European ash dieback disease has a recent Eastern Asiatic origin. The distinctly higher genetic diversity found in Japanese *Hy. fraxineus* in contrast to European *Hy. fraxineus* supports this view. Genetic similarities between Japanese *Hy. fraxineus* and European *Hy. albidus* suggest that also *Hy. albidus* might be a descendant of Asian *Hy. fraxineus*, though having invaded Europe much earlier. However, consistent genetic deviation between European and Asian *Hy. fraxineus* at two nucleotide positions of the ITS region indicates that the European ash disease originates from a region different from the presently known areas in Eastern Asia.

Our results underline the importance of detailed morphological studies in combination with molecular work. *Hy. fraxineus* was described from Europe as a cryptic species that differed from *Hy. albidus* by molecular data alone. However, the *Hy. albidus*/*Hy. fraxineus* species complex represents one of many examples within the ascomycetes in which subtle microscopic differences between closely related species, in this case the presence or absence of croziers at the ascus base, are strictly correlated with molecular characteristics. Two species that closely resemble *Hy. albidus* and *Hy. fraxineus* form pseudosclerotia in *Aesculus* leaves and again differ from each other mainly in the ascus base: *Hymenoscyphus aesculi* on *Aesculus hippocastanum* from Europe lacks croziers, whereas *Hymenoscyphus honshuanus* from Japan on *Aesculus turbinata* possesses croziers. Other taxa treated here include *Hymenoscyphus vacini*, a European species growing on stromatized net veins of skeletonized leaves of *Acer*, and *Hymenoscyphus torquatus*, a Chinese species on unidentified herbaceous stems. An equivalent stroma-forming North American species on leaves of *Fraxinus, Rutstroemia longipes* (Rutstroemiaceae), is discussed and compared. A key to the *Hymenoscyphus* species that form a dark stroma on leaves of *Acer, Aesculus, Fraxinus,* and *Picrasma* is provided.

## Introduction

Ash dieback is a serious disease in Europe causing death of European ash. The disease is caused by *Hymenoscyphus fraxineus* (T. Kowalski) Baral et al. (≡*Chalara fraxinea* T. Kowalski, =*Hymenoscyphus pseudoalbidus* Queloz et al.), which was recently introduced from Asia and has rapidly replaced the non-pathogenic European species *Hymenoscyphus albidus*. The present study outlines the introduction and cause of the disease, and compares closely related harmless species on ash and other hosts.

### *Hymenoscyphus albidus*, a saprotroph on leaves of European ash

In late summer and early autumn, *Hy. albidus* (Roberge ex Gillet) W. Phillips forms white stipitate apothecia on fallen, previous year’s rachises (here used for the entire main axis including the basal petiole) and leaflet veins of the pinnate leaves of ash (*Fraxinus excelsior*, Oleaceae, Lamiales). During the past 150 years, the species has infrequently been recorded in temperate and montane Europe. It can be assumed that the ascospores infect living leaves, and the mycelium grows endophytically inside them, similar as it is known in *Hy. fraxineus*. Quite a long time after the leaves have fallen, and after the leaf blades have disappeared, the fungus forms a very thin black stromatic layer on the surface of the remaining rachises and veins, which is referred to as ‘pseudosclerotial plate’. This layer becomes slowly apparent during the winter months, and might serve as a protection of the underlying hyphae against ultraviolet light, or to avoid invasion into the pseudosclerotium by hyphae of other species. The terms pseudosclerotium and pseudosclerotial plate were adopted by Kowalski and Holdenrieder (2009) and Queloz et al. (2011) in the *Hy. albidus* complex, although they are somewhat ambiguously defined (see Gross & Holdenrieder 2013).

Due to the black pseudosclerotium, the fungus was placed by some authors in the genus *Lanzia* Sacc. or *Lambertella* Höhn. (Sclerotiniaceae, now Rutstroemiaceae). However, the type of ascus apical ring and the heteropolar (scutuloid) ascospores refer it to the genus *Hymenoscyphus* Gray (Baral & Krieglsteiner 1985, p. 121). This placement was meanwhile confirmed by molecular data (Queloz et al. 2011).

### Appearance of a second species of *Hymenoscyphus*

Recent research on the current epidemic ash dieback in Europe revealed the asexual state *Ch. fraxinea* as the causal agent of ash disease (Kowalski 2006), which, by molecular comparison, proved to be connected to a sexual morph (Kowalski & Holdenrieder 2009), though at first misidentified as *Hymenoscyphus albidus*. A molecular study by Queloz et al. (2011) on a variety of teleomorph collections, mainly from Switzerland, revealed that in fact two different ascocarp-forming species fruit on the blackened rachises of ash, which could not be distinguished morphologically. One of them (*Hy. albidus*) proved to be rather harmless, whereas the other represented the serious pathogen. The latter was described as a new species, *Hy. pseudoalbidus* Queloz et al., based mainly on the deviating rDNA sequence. It formed slightly longer ascospores and larger fruit bodies, but was otherwise thought to concur completely with *Hy. albidus* and was, therefore, referred to as a ‘cryptic species’ (Queloz et al. 2011).

Apothecia of *Hy. fraxineus*, as the holomorph of *Ch. fraxinea*/*Hy. pseudoalbidus* is now called, have been collected in Europe at least since 2006 (two collections dating from 1978 and 1987 as listed in the specimens examined by Queloz et al. 2011 were based on confused DNA sequences and concern *Hy. albidus*, Queloz et al. 2012). The symptoms of leaf wilting were first observed in the northeastern part of Poland in 1992 (Kirisits et al. 2009), and the first isolate of its asexual state dates from December 2000 and represents the type culture of *Ch. fraxinea* (Kowalski 2006).

Between 2000 and 2010, the invasive fungus spread to the west and south of Central Europe, to southern parts of Northern Europe, and to Eastern and Southeastern Europe (Kirisits 2010), and during 2008–2013 it invaded Great Britain (Hendry 2013). In the last years, *Hy. albidus* could still be found in high-montane areas of Central Europe (see Queloz et al. 2011), Southern and Southeastern Europe (N. Matočec & I. Kušan personal communication), in Atlantic lowland regions of Western Europe and in boreal to continental regions of Northern and Eastern Europe, where *Hy. fraxineus* did so far only as an exception expand or now occurs sympatric. *Hy. albidus* appears to be extinct all over Central Europe north of the Alps.

Besides the here treated *Hymenoscyphus honshuanus* Baral, *Hymenoscyphus aesculi* (Velen.) Baral & E. Rubio, and *Hymenoscyphus vacini* (Velen.) Baral & E. Weber, a further species was recently described, *Hy. albidoides* (Zheng & Zhuang 2014). It was found fruiting in Eastern China on blackened veins of leaves of *Picrasma quassioides* (Simaroubaceae, Sapindales). In its morphological characters, this species can hardly be distinguished from *Hy. fraxineus*, except for the shape of the crystals in the stipe base, but its molecular data significantly deviate. Another species was recently detected in Japan growing on rachises of *Fraxinus platypodia*, which will be described in a separate paper as *Hymenoscyphus linearis* Hosoya, A. Gross & Baral. It concurs microscopically quite well with *Hy. albidus* but forms very narrow, linear stromata of considerable length. We have included both species in our dichotomous key.

### Purpose of study

The observation by the first author of striking and unequivocal deviations in the ascus base within European populations collected in 1988–1991 (simple septate) and 2006–2010 (croziers) raised the question whether this character is correlated with the observed molecular markers and, as a consequence, *Hy. albidus* can be distinguished from *Hy. fraxineus* by morphological methods. In course of this study, a large number of fresh as well as herbarium specimens were examined in order to verify the presence or absence of croziers and to compare the result with the available molecular data. A detailed documentation of teleomorph morphology was made in order to clarify whether further distinguishing features exist. Japanese herbarium material of *H fraxineus* [originally identified as *Lambertella albida* (Roberge ex Gillet) Korf] was included in this study in order to find out whether differences exist to European *Hy. fraxineus*.

## Materials and methods

### Microscopy

Collections were examined preferably in the living state, but also from rehydrated herbarium material, using a Zeiss Standard 14 and a Zeiss Standard KF microscope equipped with achromatic and plan-apochromatic objectives. Tap water (H_2_O) was used as a standard medium, and viability of cells was evaluated in that medium according to Baral (1992). The iodine reaction was tested with Lugol’s solution (IKI = ~1% I_2_, 2% KI, in H_2_O), without (rarely with) pre-treatment by potassium hydroxide (KOH 3–5%). Brilliant Cresyl Blue (CRB, ~1% in H_2_O) added to a water mount was used for testing the presence of gel, also for vital staining of the refractive vacuoles (VBs). For testing the presence of croziers, fresh apothecia were sectioned free-hand, and sections transferred to the slide in a drop of H_2_O; any pressure on the cover slip was avoided during preparation. In the case of herbarium specimens, hymenial fragments (mainly sections) were placed in a drop of H_2_O, to which a small drop of KOH and often also one of aqueous Congo Red (CR) was added. Alternatively, CR_SDS_ (SDS = sodium dodecyl sulphate) was applied to a water mount. Dissolution of the oil drops (lipid bodies, LBs) by ethanol was conducted as follows: a dry apothecium was rehydrated and a fragment placed in 90% ethanol for ½ min; then the ethanol was removed and a drop of 3% KOH and one of 1% phloxine was added. Waterman blue-black ink was applied for a better visibility of ascospore sheaths. Photographic images (macro- and microphotos) were obtained using a Nikon Coolpix E4500 and a Nikon Coolpix 5000 (Nikon Corporation, Tokyo, Japan). All drawings are free-hand.

### Abbreviations

* = living state, † = dead state, n.v. = *non visus* (specimen or documentation not seen by us), d.v. = documentum visus (photos/drawings/descriptions seen by us), sq. = sequence of ITS1-5.8S-ITS2 region, vid. = examined, ø = unpreserved, # = not tested for the ascus base. Values in curled parenthesis {} refer to the number of collections that were examined or, after the host plant and the associated taxa, the number of certain/uncertain records. Countries are abbreviated according to the ISO 3166 two-letter standard code.

### Herbaria

Type and other herbarium material was studied from CUP (Cornell University, Ithaca, New York), HMIPC (Mycological Herbarium of the Department of Forest Pathology of Kraków), LUX (Musée national d’histoire naturelle Luxembourg), NMLU (Natur-Museum Luzern), PRM (National Museum, Praha), TNS (National Museum of Nature and Science, Tsukuba), and ZT (Eidgenössische Technische Hochschule Zürich), and two specimens were deposited in STU (Staatliches Museum für Naturkunde Stuttgart) and KR (Staatliches Museum für Naturkunde Karlsruhe). Mentioned herbaria from which material was not examined are the following: FH (Harvard University, Cambridge, Massachusetts), GENT (Ghent University, Belgium), K (Royal Botanic Gardens, Kew, London), KUS (Korea University), PC (Muséum National d’Histoire Naturelle, Paris). Additional collections are held in the private herbaria of A.G. = Alain Gardiennet (Véronnes, Côte d’Or), B.D. = Bernard Declercq (Stekene, Eastern Flanders), C.B. = Céline Besch (†, Mertert, Luxembourg, in LUX), C.Y. = Chris Yeates (Huddersfield, West Yorkshire), E.R.D. = Enrique Rubio (Salinas near Avilés, Asturias), G.K. = Gerhard Koller (Mattersburg Burgenland), G.M. = Guy Marson (Hesperange, Luxembourg), H.B. = H.-O. Baral, H.E. = Heinz Engel (Weidhausen near Coburg), H.H. = Hans Haas (†, Stuttgart, in STU), M.B. = Martin Bemmann, M.H. = Michel Hairaud (Poivendre de Marigny, Deux-Sèvres), M.T. = Marie-Thérèse Tholl (Doncols, Luxembourg), R.D. = René Dougoud (Fribourg, Switzerland), S.Å.H. = Sven-Åke Hanson, S.H. = Stip Helleman (Boxmeer, Noord-Brabant), W.D. = Wolfgang Dämon (St. Georgen, Salzburg), W.W. = Wulfard Winterhoff (Sandhausen near Heidelberg).

### Taxonomy

#### *Hymenoscyphus albidus* (Roberge ex Gillet) W. Phillips, Man. Brit. Discomyc. (London): 138 (1887) (as *Hymenoscypha albida*) – Figures 1 **–** 4

**Figure 1.**
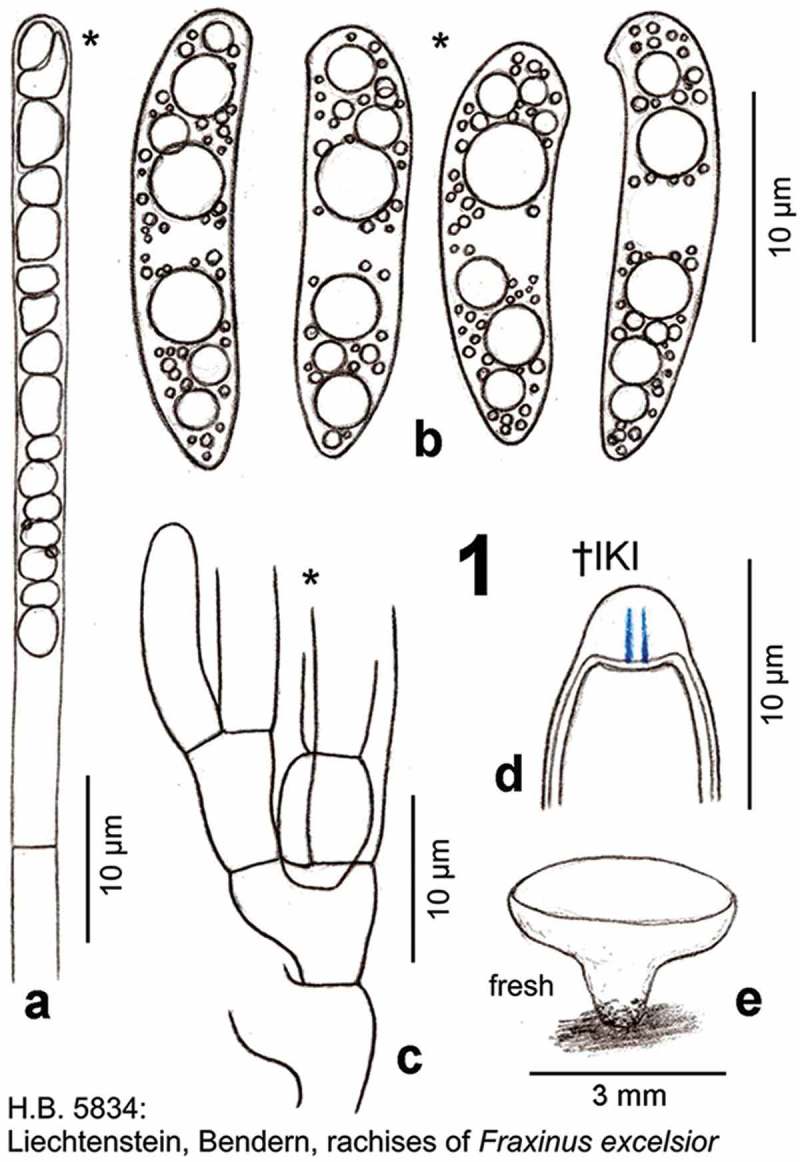
*Hymenoscyphus albidus*: a. paraphysis containing vacuolar bodies (=VBs); b. freshly ejected ascospores, containing oil drops (=LBs); c. simple-septate ascogenous hyphae without crozier formation; d. ascus apex with euamyloid apical ring (*Hymenoscyphus*-type); e. apothecium. – living state (except for d).

**Figure 2.**
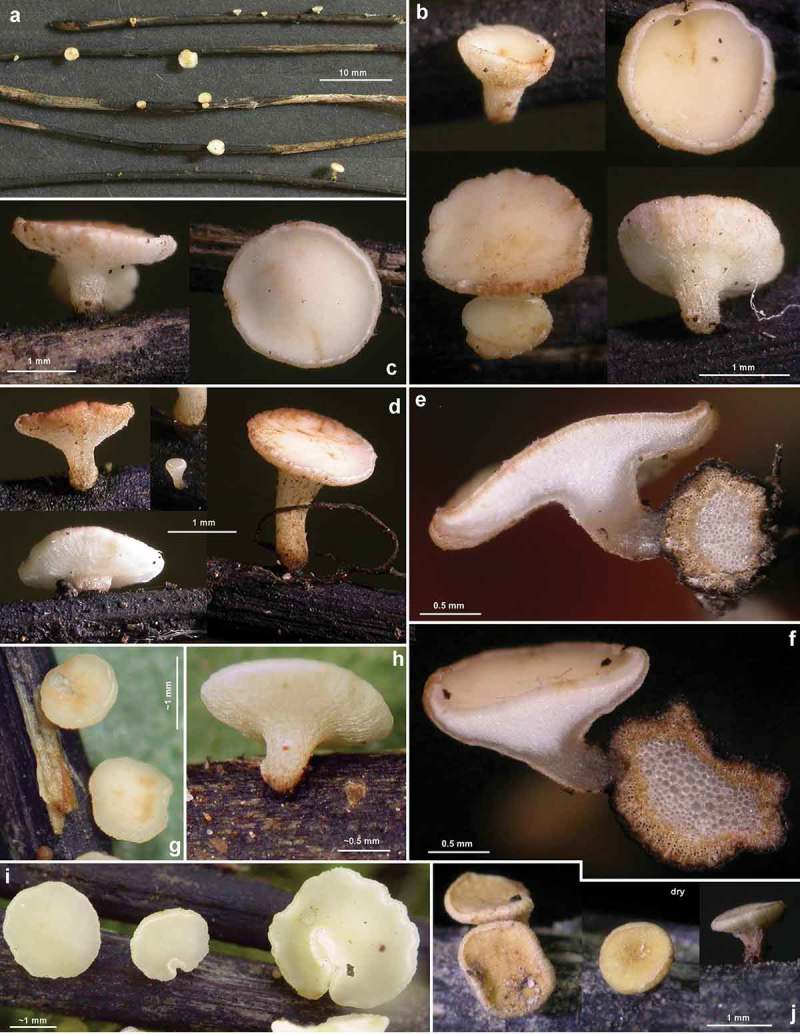
*Hymenoscyphus albidus*: a–j. apothecia emerging from black pseudosclerotia in rachises of *Fraxinus excelsior* (e–f: in median section). – a–i: fresh state, j: dry state. – a–f. H.B. 9699 (FR-PC, Granzay-Gript); g–h. 21.VII.2007 (CH-ZG, Unterägeri); i. 24.VI.2007 (FR-PC, Poitevin); j. H.B. 9454 (CH-LU, Aesch). – Phot. g–h: U. Graf, i: M. Hairaud.

**Figure 3.**
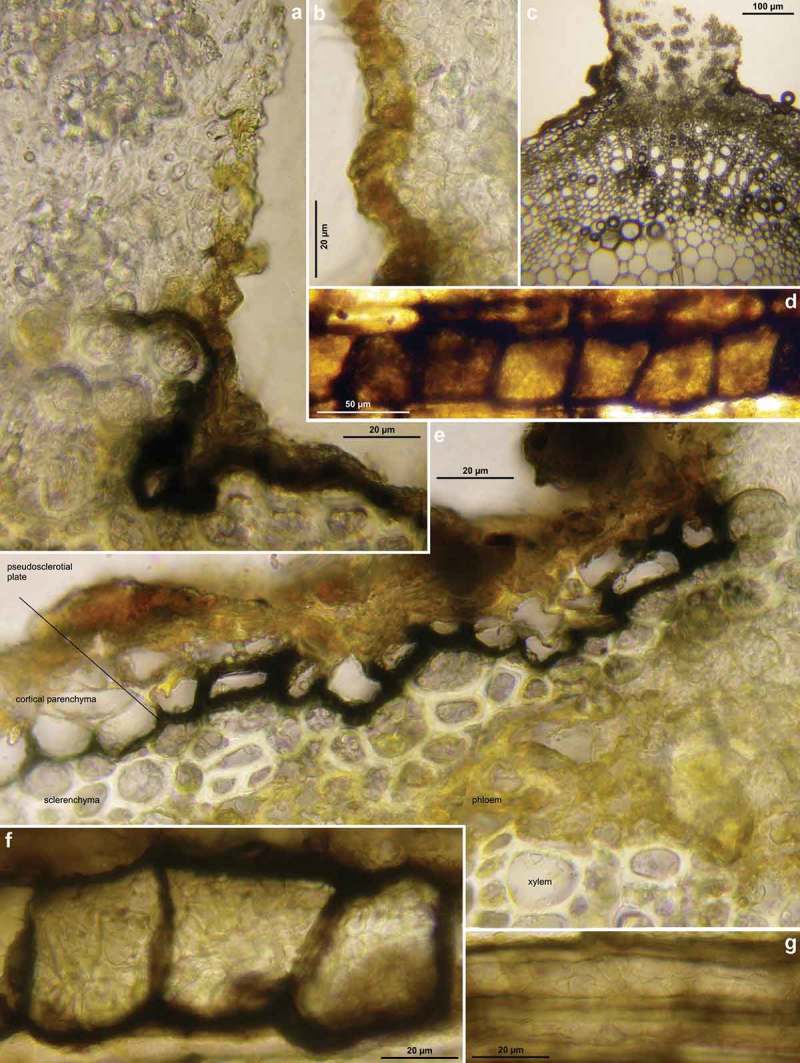
*Hymenoscyphus albidus*: a–c, e. median section of stipe base (with internal crystals) and cross section of pseudosclerotium in ash rachis (the black demarcation line is restricted to the border between cortical parenchyma and sclerenchyma, hyphae present in all tissues of petiole); d, f–g. external view on pseudosclerotial plate (tangential section of rachis surface), cells of cortical parenchyma and sclerenchyma densely filled with subhyaline hyphae (textura epidermoidea). – All in living state. – a–g. H.B. 9699 (FR-PC, Granzay-Gript).

**Figure 4.**
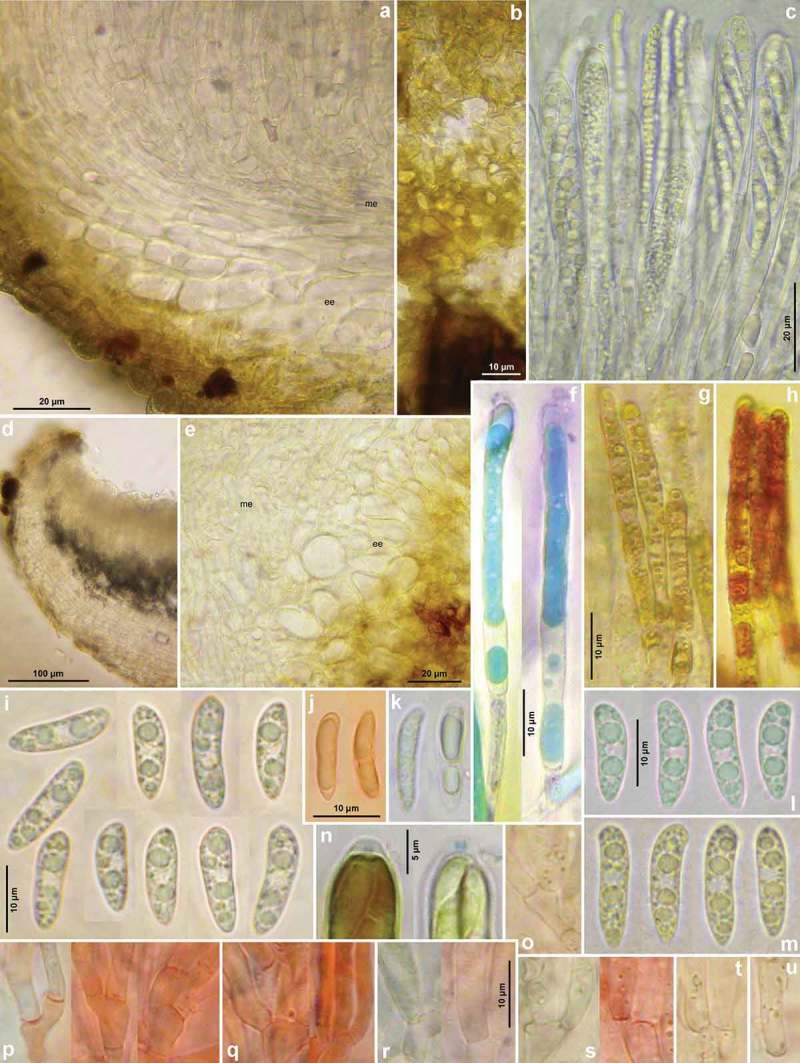
*Hymenoscyphus albidus*: a, d. median section of receptacle (ee = ectal excipulum, me = medullary excipulum); b. surface view on bright yellowish-ochraceous exudate covering the stipe base; c. paraphyses containing guttular VBs, immature and mature asci; e. median section of ectal excipulum at transition of stipe and receptacle; f–h. paraphyses (containing ± elongate VBs; f: under vital staining, g–h: VBs distorted in dead state); i–m. ascospores (containing large and small LBs; j–k: dead shrunken spores showing confluent LBs); n. ascus apices with euamyloid apical ring; p–u. ascus bases arising from simple septa. – Living state: a–d, f (in CRB), i, l–m; dead state: g (in H_2_O), h (in IKI), n (in IKI), j–k, p–u (in KOH or KOH+CR). – a–b, d–j, l. H.B. 9699 (FR-PC, Granzay-Gript); c, m. 16.VI.2007 (ibid.); k. H.B. 9611 (BE-LUX, Florenville); n. LUX 047699 (LU, Kehlen); o. ZT 3295 (CH-TI, Aquarossa); p. 21.VII.2007 (CH-ZG, Unterägeri); q. H.B. 9454 (CH-LU, Aesch); r. H.B. 1950 (DE-BW, Stockach); s. ZT 3299 (CH-BE, Eymatt); t. ZT 3294 (CH-TI, Lavorgo); u. ZT 3293 (FR-BN, Bellême). – Phot. c, l–m: M. Hairaud.

≡ *Peziza albida* Roberge in Desm., Pl. Crypt. N. France, #2004 (1850), Annls. Sci. Nat., Bot., sér. 3, 16: 323 (1851), nom. illegit. [ICBN Art. 53.1, non *Peziza albida* With. 1792, nec *P. albida* Sowerby 1799, nec *P. albida* (All.) Lam. 1804]
**≡** *Helotium scutula* var. *albidum* (Roberge in Desm.) P. Karst. Bidr. Känn. Finl. Nat. Folk 19: 112 (1871) [nom. nov., ICBN Art. 58, as *He. scutula* var. η *albidum* (Desm.) Karst.]
**≡** *Phialea albida* Roberge ex Gillet, Champignons de France, Discomyc., livr. 4, p. 105 (1881) [1879] [nom. nov., ICBN Art. 58, as *Phialea albida* Desm.]
**≡** *Helotium albidum* (Roberge ex Gillet) Pat., Tabl. analyt. Fung. France (Paris) 4: 173 (1885) nom. illegit. [ICBN Art. 53.1, non *Helotium albidum* P. & H. Crouan 1867, nec *Helotium albidum* (With.) P. Karst. 1884 (as ‘*albellum*’)]
**≡** *Helotium robergei* Dennis, Mycol. Pap. **62**: 93 (1956) [nom. nov., ICBN Art. 53.1]
**≡** *Lanzia albida* (Roberge ex Gillet) S.E. Carp., Mem. N. Y. Bot. Gdn **33**: 187 (1981) [as (Roberge & Desm.) S.E. Carp.]
**≡** *Lambertella albida* (Roberge ex Gillet) Korf, Mycotaxon **14(1)**: 2 (1982)

##### Etymology

*Albidus*: named after the white colour of the apothecia, *robergei*: after M.R. Roberge.

##### Lectoype *(selected by White (1944))*

France, Calvados, [(?) NE of Caen, Bois de Lébisey,] rachises of *F. excelsior*, summer ca. 1850, M.R. Roberge (Desm., Pl. Crypt. N. France, Ed. 1, n° 2004, FH; not seen).

##### Apothecia

Moist (0.8–)1–2.5(–4) mm diam., receptacle 0.24–0.4 mm thick (at margin 0.15–0.2 mm), ±round, scattered; disc whitish to very pale cream, turning reddish-brown when bruised, slightly concave to flat; margin distinct, up to 70 µm protruding, finely pubescent, exterior concolorous, stipe 0.2–1.5 mm long, 0.25–0.5 mm wide at the base, 0.4–7(–1) mm below receptacle, white, pubescent, whole exterior finely striate, turning reddish-brown with age, at base light reddish-brown, with a black basal collar, ±erumpent from sclerenchyma-phloem layer; dry with cream-ochraceous to orange disc. **Asci** *(75–)80–110(–125) {5} × 9–11.5 µm {4}, †(67–)75–100(–116) {7} × 7.5–8.5 {2} or (8.5–)9–9.5(–10) {5} µm, 8-spored, spores *obliquely biseriate, pars sporifera *44–53 µm long; **apex** (†) conical, dome †2–3 → 1–1.3 µm thick, apical ring occupying lower 1/3–3/4 of dome, medium to strongly blue (bb) in IKI, ring apically gradually or abruptly fading, *Hymenoscyphus*-type {8}; **base** arising from simple septa {27}, never with a subbasal protuberance. **Ascospores** *(13.5–)14.5–18.5(–20.5) × (3.5–)4–4.8(–5) µm {9}, †(13–)14–19(–21.3) × (3–)3.5–4.2(–4.5) µm {6}, slightly to strongly scutuloid, with ± cylindrical middle part, apex rounded to obtuse, with a slight to strong lateral protrusion, base medium attenuated, with ± blunt, never acute end, straight to inequilateral or medium curved, especially at the apex, containing 1–3(–4) large LBs in each half [(1–)2–3(–3.5) µm diam.], associated with many small LBs 0.3–1 µm diam. {6}, lipid content 5, rarely a very delicate sheath was seen that slips off the spore, without setulae; wall surface CRB–; overmature spores not observed. **Paraphyses** apically uninflated, terminal cell *40–61 × 3–4 µm {1}, †40–62 × 2–2.7 µm {1}, lower cells *9–23(–27) × (2–)3–4 µm {1}, †12.5–22 × 2–3 µm {1}; branched only in lower part, anastomoses not seen. **Medullary excipulum** hyaline, of a loose textura intricata, individual cells *(20–)35–73(–80) × (3–)5–10(–16) µm {2}, delimited from ectal excipulum by an 50–100 µm thick layer of textura porrecta, cells *35–85 × 3–8 µm, all hyphae covered by thin gel staining deep lilac in CRB. **Ectal excipulum** hyaline, at flanks of (*) slightly († medium) gelatinized textura prismatica(–porrecta), 30–40 µm thick, cells *15–35(–50) × (6–)8–13(–17) µm {1}, †7–11(–14) µm wide {1}, oriented at a (0–)10–30(–40)° angle to the surface, walls †1–1.5(–2) µm thick, common walls 2–4 µm, CRB–; 20–25 µm thick at margin, oriented at 0°; in stipe of 25–50 µm thick, of (†) slightly gelatinized t. angularis(-globulosa) oriented irregularly or t. prismatica oriented at 0°, cells *7.5–15(–20) × 5–7 or 7–10(–17 µm) {1}; whole exterior of receptacle and stipe covered by a 10–15 µm thick layer of cortical hyphae forming a loose network in surface view, individual cells *11–20 × 3–5 µm, partly forming projecting or appressed **hair-like protrusions** *10–45 × 3–5 µm, 1–3-celled, straight to irregularly flexuous. **Crystals** absent in complete tissue, except for medullary tissue in lower region of stipe at a length of ~200 µm, here forming abundant druses 10–20 µm diam., made up of rhomboid, KOH-inert individual crystals 7–15 µm diam. **VBs** in paraphyses medium to strongly refractive {6}, hyaline, turning light yellowish-ochre in dead state, young multiguttulate then angular to shortly elongate, finally very elongate, occupying upper 30–50(–70) µm {3} (terminal and partly first lower cell), staining bright turquoise in CRB and light reddish-brown in IKI. **Exudate** pale yellowish-ochraceous, smooth to rough, covering cortical hyphae of receptacle and stipe, more bright-coloured towards base of stipe. **Anamorph**: unknown.

##### Habitat

various subassociations of *Alnetum, Fagetum, Fraxinetum, Fraxino-Aceretum, Pruno-Fraxinetum, Salicetum*, (*Querco*-)*Ulmetum*, on fallen previous year’s blackened rachises {34} or rarely primary veins {4}, exceptionally on a corticated, blackened, 1 mm thick twig {1}, of *F. excelsior* {36}, lying on ground. **Assoc.**: None. **Desiccation tolerance**: not tested. **Geology**: basalt, limestone, sandstone, Knollenmergel. **Phenology**: (VI–)VII–IX(–X). **Altitude**: 3–1690 m.

##### Specimens included

All on rachises of *F. excelsior*, two records on *Acer* possibly concern in fact *Fraxinus*; list incomplete concerning records not examined by us.

**Sweden**: **Skåne,** 7.5 km ENE of Helsingborg, 1.2 km SSE of Kropp, Ljungberga, 42 m, 24.VIII.1994, S.Å. Hanson (S.Å.H. 15290). – The **Netherlands**: **Gelderland**, 6 km SW of Nijmegen, W of Meijhorst, Staddijkpark, 8 m, 7.VII.2007, S. Helleman (S.H. 444, H.B. 9854). – **Belgium**: **West Vlaanderen**, 5 km ENE of Kortrijk, 1.8 km SE of Harelbeke, De Gavers, 14 m, 30.IX.1989, B. Declercq (B.D. 89/118, n.v.). – 17 km NNE of Brugge, ESE of Knokke-Zoute, Koningsbos, 8 m, 24.VIII.1994, B. Declercq (B.D. 94/108, as *Pyrus communis*, H.B. 9847). – **Oost Vlaanderen**, 24 km NE of Ghent, 3.2 km NW of Sinaai, Heirnisse, 5 m, 16.VII.1994, B. Declercq (B.D. 94/092, d.v.). – **Luxembourg**, 21 km SW of Arlon, 3 km NNE of Virton, Bois de Virton, 250 m, 13.VIII.1989, B. Declercq (B.D. 89/076, d.v.). – ~7 km SSE of Florenville, Orval, ~200 m, partly on veins, ~5.IX.1990, C. Besch 1091 (C.B. 1091, LUX 047702, H.B. 9611ø). – **Luxembourg**: **Gutland**, 7 km W of Echternach, Schnellert, ~200 m, partly on a twig, 14.VII.1985, C. Besch (C.B. 183, LUX 047701, H.B. 9612ø). – 3 km NE of Grevenmacher, 1 km WNW of Mertert, N of Schënnerkaul, S of Fausermillen, 155 m, on petiole of ‘*Acer pseudoplatanus*’, 24.IX.1990, H.O. Baral, C. Besch & C.M. Swart-Velthuyzen (ø). – 8 km NW of Luxembourg, 1.3 km E of Kehlen, Maasselter, 370 m, 17.VII.1988, C. Besch (C.B. 181, LUX 047699, H.B. 9619ø). – 3 km N of Grevenmacher, E of Manternach, Syre, ~200 m, 15.VIII.1985, C. Besch (C.B. 182, LUX 047700, H.B. 9620ø). – **Germany**: **Rheinland-Pfalz**, 9 km NE of Pirmasens, 3 km SE of Waldfischbach-Burgalben, near Ruine Heidelsburg, 280 m, partly on veins, ~3.IX.1989, B. Mauer (H.B. 3841, sq.). – **Baden-Württemberg**, 6 km NE of Tübingen, 0.5 km ENE of Pfrondorf, Tiefenbach, 400 m, partly on veins, 14.IX.1991, E. Weber & H.O. Baral (ø). – ibid., on primary veins of ‘?*Acer*’, 3.IX.1988, H.O. Baral (ø). – 12 km W of Stockach, N of Aach, 550 m, 20.VIII.1976, H.O. Baral (H.B. 1950, sq.). – ~4 km NNE of Emmendingen, Stilzerfritz, 350 m, 2.IX.1975, H.O. Baral (H.B. 410ø, #). – ~8 km N of Breisach, ~1 km NW of Burkheim, Rheinauen, 190 m, 6.IX.1975, H.O. Baral (H.B. 411ø, #). – Konstanz, 4 km NE of Radolfzell, 1 km SE of Möggingen, Mindelsee, 410 m, 30.VII.1975, H.O. Baral (STU, ex H.B. 376, H.H. 10409). – 1.6 km SE of Möggingen, Mindelsee, 410 m, 28.VIII.1976, H.O. Baral (ø, #). – 8 km NE of Radolfzell, 2.7 km SE of Bodman, Steckenloch, 440 m, 18.VIII.1976, H.O. Baral (ø, #). – 6.5 km NE of Radolfzell, 1.7 km SW of Bodman, Dettelbach, 500 m, 22.VIII.1976, H.O. Baral (ø, #). – ibid., 460 m, 17.VIII.1977, H.O. Baral (ø, #). – **Bayern**, 13 km ENE of Ulm, Unterfahlheim, Rühmerhalbinsel, 460 m, 15.VIII.1979, M. Enderle (ø, #). – **Liechtenstein**: 7 km NNW of Vaduz, 1.4 km SW of Bendern, Bannriet, 445 m, 5.VII.1997, E. Weber & J.P. Prongué (H.B. 5834, sq.). – **Switzerland**: **Fribourg**, 15.5 km NW of Bulle, 1 km SE of Villars-Bramard, Bois de Boulogne, 820 m, 14.VIII.2011, R. Dougoud (ZT, ex R.D. 32.28.094.11, n.v.). – 1.1 km E of Villars-Bramard, Bois de Boulogne, Le Mothey, 800 m, 14.VIII.2011, R. Dougoud (ZT, ex R.D. 32.29.094.11, d.v.). – **Bern**, 4.5 km WNW of Bern, 0.4 km ENE of Eymatt, Bremgartenwald, 508 m, 5.VIII.2009, B. Senn-Irlet (ZT 3300, sq.: GU586878). – 0.5 km E of Eymatt, 530 m, 5.VIII.2009, B. Senn-Irlet (ZT 3299, sq.: GU586877). – **Luzern**, 24 km NNW of Luzern, 1.2 km E of Aesch, Gitzitobel, 530 m, 4.IX.1978, F. Müller (ZT, H.B. 9454ø, ex NMLU 0409–78 FM 1, sq.: GU586910). – **Zug**, 3 km SSW of Unterägeri, 0.3 km E of Ochsenfeissi, Haslen, 830 m, 21.VII.2007, U. Graf (ZT 3891, ex NMLU 2107–07, sq.: GU586897). – **Ticino**, 0.8 km S of Quinto, 0.4 km WNW of Varenzo, 990 m, 26.VII.2009, V. Queloz (ZT 3296, sq.: GU586887, 586888, 586889). – 1.6 km N of Aquarossa, 0.7 km ENE of Castro, 597 m, 28.VII.2009, V. Queloz (ZT 3295, sq.: GU586882, 586883). – 0.3 km SSW of Lavorgo, 616 m, 27.VII.2009, V. Queloz (ZT 3294, sq.: GU586884, 586885, 586886). – **France**: **Basse-Normandie, Calvados**, (?) c. 4 km NE of Caen, (?) Bois de Lébisey, (?) 65 m, summer ca. 1850, M.R. Roberge (Desm., Pl. Crypt. N. France, Ed. 1, n° 2004, FH, **lectotype; isolectotypes** in GENT, K, PC, d.v., sq.: HM193466; Ed. 2, n° 1604, **isolectotypes in** GENT, FH, K, PC, d.v., sq.: HM193465). – **Orne**, 3 km NNW of Bellême, near La Herse, 190 m, 28.IX.1995, R. Dougoud (ZT 3293, ex R.D. 22.11.094.95, sq.: GU586876). – **Poitous-Charentes, Deux Sèvres**, 12.5 km WNW of Niort, 1 km ENE of Le Vanneau-Irleau, Marais Poitevin, 3 m, 24.VI.2007, M. Hairaud (ø, #, d.v.). – 10.5 km SSW of Niort, 1.8 km NW of Granzay-Gript, La Courance, 25 m, 16.VI.2007, M. Hairaud (M.H. 220607ø, #, d.v.). – ibid., 2.VII.2012, M. Hairaud (M.H. 10712, H.B. 9699). – ibid., 8.VII.2014 (ø, n.v.). – 2 km E of Chives, N of Le Vivier Jusseau, 85 m, 5.IX.2012, M. Hairaud (M.H. 050812, n.v.). – ibid., 10.VIII.2013 (ø, n.v.). – S of Bassac, 18 m, 5.VII.2012, M. Hairaud (ø, n.v.). ― **Spain**: **Vasca,** Gipuzkoa, 2.2 kmWNW of Aia, 2 km ESE of Urdaneta, Mindi erreka, 295 m, 25.VII.2014, J. Teres, vid. I. Olariaga (ARAN-Fungi 000082, d.v.). ― **Atxondogoikoa (Zeanuri)**, 9 km WNW of Otxandio, 13 km SE of Orozko, 600 m, 18.VIII.2014, J.V. Fernandez (M.B. 10/2014).

##### Characterization

Morphologically, *Hymenoscyphus albidus* can be differentiated from *Hy. fraxineus* with certainty only by the absence of croziers at the ascus base. Further features include comparatively small apothecia (up to 4 mm diam.) growing on often rather small, insular pseudosclerotia, slightly smaller ascospores, and a rather late start of fructification, also the absence of an anamorph in pure culture.

##### Nomenclature

Korf (1982) and Hosoya et al. (1993) cited Desmazières’s exsiccatum ‘Plant. Crypt. N. France, #2004, 1850’ as basionym of *Peziza albida* Roberge in Desm., whereas other authors considered the first valid publication to be that of Desmazières (1851, p. 323). A description was only supplied in 1851, where Desmazières referred to the exsiccatum as ‘Rob. in herb.’. The label on the exsiccatum comprises notes on the substrate (on blackened areas of half-rotten rachises of *F. excelsior*) and phenology (summer), as well as a comparison with *Peziza inflexa* (=*Cyathicula coronata*), which is said to resemble in its pale colour and cyathiform shape. Since the Code does not require a minimum text for a diagnosis, Desmazières’ label from his widely distributed edition of exsiccata of Pl. Crypt. N. France meets the requirements for a valid publication of the new taxon. The date of 1850 is somewhat uncertain, however. According to Stafleu and Cowan (1976), the title pages of the fascicles of Pl. Crypt. N. France were preprinted in some cases before distribution and may give a too early date, thus the exact citation of *Peziza albida* remains unsettled.

Nevertheless, the name *Peziza albida* is illegitimate because it is a homonym of several older binomials. Therefore, the first legitimate name of the fungus at the species level is *Phialea albida* Roberge ex Gillet (1881) or *Phialea albida* Gillet, and this binomial is treated as a nomen novum attributed to Gillet. Likewise, *He. albidum* (Roberge ex Gillet) Pat. is illegitimate because of homonymy to older binomials. Dennis (1956) created the epithet *robergei* in order to avoid such homonymy within the genus *Helotium* Pers. The name *He. robergei* was in use until *Helotium* was abandoned by Dennis (1964) in favour of *Hymenoscyphus*, because of the older *Helotium* Tode (=*Omphalina* Quél.). Sharma (1976) and Svrček (1979) combined the epithet *robergei* in *Hymenoscyphus*, which is impermissible because here no older competing homonym with the epithet *albidus* exists.

Some workers have cited the authors of *Hymenoscyphus albidus* as ‘Roberge ex Desm.’, which would imply that Desmazières validated an invalid diagnosis of Roberge. On the label of Desmazières ‘Plantes Cryptogames Nord du France’ (1850) and in his ‘Notice sur les Plantes Cryptogames’ (1851), the authorship of the diagnosis and discussion to each species is actually difficult to know. We arrived at the conclusion that Desmazières considered himself as the author of a taxon only in those cases of his 1851 paper where ‘Desm.’ or ‘Nob.’ is mentioned after the fungal name. This is not the case in *Peziza albida* where he wrote ‘Rob. in herb.’. Also we believe that the author of the printed diagnoses of 1850 and 1851 is Desmazières, unless we would gain knowledge of a manuscript or correspondence proving different authorship. The remark ‘Desmaz.’ after the ecology in the 1851 paper is found in all those cases where the remark ‘Rob. in herb.’ or ‘Nob. in herb.’ appears after the fungal binomial, irrespective of whether or not an exsiccatum is cited. We interpret the remark ‘Desmaz.’ as an indication of the authorship of the diagnosis but not of the collection. The phrases ‘Rob.’ or ‘Rob. in herb.’ after the binomial on the printed label of 1850 and the article of 1851 indicate that Desmazières attributed the authorship of the taxon to Roberge, who was probably merely the collector, while Desmazières supplied the description, including the microscopical data.

Shortly after the demise of Roberge and Desmazières, authors were still quite consistent in their citation. Indeed, many of those in the nineteenth century followed Desmazières by citing the author of the taxon as ‘Rob.’, e.g., Roumeguère (1870, p. 130), Berkeley and Broome (1878, p. 29), Patouillard (1885, p. 173), Phillips (1887, p. 138), Rehm (1893, p. 797), and Massee and Crossland (1905, p. 285). Consistent with this citation is the variant ‘Rob. in Desm.’, which can earliest be found in Phillips (1887), and was adopted by Korf (1982) and Hosoya et al. (1993). In contrast, Roumeguère (1891, 1892) cited the authors as ‘R. et D.’ (Roberge & Desm.), hence he considered Desmazières as a coauthor. This version was followed by Boudier (1907, p. 111, 1908, p. 287), Schröter (1893, p. 74), Migula (1913, p. 1167), Dennis (1956), Arendholz (1979), and Carpenter (1981).

A very different view was adopted by Karsten (1871, p. 112), Cooke (1876, p. 132), and Gillet (1881, p. 105), who merely used the citation ‘Desm.’. The similar version ‘Rob. ex Desm.’, used by White (1944) and Cannon et al. (1985), considers Desmazières as a validating author.

We are inclined to adopt here the version ‘Roberge in Desm.’, because Desmazières attributed the authorship to Roberge. In any case, the original binomial was invalid due to homonymy; therefore, the correct author citation for combinations other than *Peziza albida* is Roberge ex Gillet or merely Gillet.

##### History of type studies

In or shortly before 1850 a rich collection was made, on which the description of *Hy. albidus* in Desmazières (1851, p. 323) is based. The sample was divided already in 1850 in a number of duplicates, and disposed as two exsiccata series in Pl. Crypt. N. France (Ed. 1, n° 2004; Ed. 2, n° 1604). For each of the two exsiccata, one duplicate was deposited in the herbarium of CN (Caen, Basse-Normandie), one came in Desmazières’ herbarium which is preserved at PC, and others are now to be found in herbaria such as FH, GENT, and K.

Desmazières (1851) mentioned the two exsiccata in his description, but without specifying which is the type. Apart from this rich collection, a herbarium specimen exists in K that bears Roberge’s handwriting and is apparently not part of the two distributed exsiccata series, but a different, later collection by Roberge, as it bears the note ‘*Peziza albida* Roberg. (Desm. 2004)’.

Nylander (1868, p. 40) examined two duplicates of *Hy. albidus*, possibly those from PC, since he was working at the Muséum national d’histoire naturelle, Paris in 1850–1858, and finally moved to Paris in 1863. He mentioned this only in a note under *Peziza albidula* (Hedw.) P. Karst. [as ‘(Hdw.) Whlnb.’, ≡ *Phialea cyathoidea* var. *albidula* (Hedw.) Rehm], in which he gave data on ascus and spore dimensions different from those of Desmazières. Karsten (1871, p. 112) made a description, apparently from type material (presumably in H), because he stated that the species was not yet collected in Finland.

Cooke (1876, p. 132, pl. LXV fig. 297) presented an uncommented microscopic drawing based on Desmazières n° 2004. Massee (1895, p. 260) supplied a description of an authentic specimen of Roberge, and also examined Desmazières n° 2004 which he considered conspecific (both in K). Like Nylander and Cooke, also Massee did not select a type. In the nineteenth century, it was not common usage to specify a single specimen as type when erecting a new taxon.

The American mycologist White (1944, p. 608, fig. 19–24) appears to be the first in the twentieth century who re-examined the type collection of *Hy. albidus*. He considered the two syntypes in FH as conspecific, and gave a thorough and precise redescription. His detailed illustration was based on n° 2004, and in the legend he wrote ‘all from **type** material, Desm. Pl. Crypt. Fr., Ed. I, 2004 (FH)’. We believe that this statement can be taken as a lectotypification (Art. 7.11, 9.21 ICBN).

When Arendholz (1979, p. 79) studied three duplicates of the two exsiccata (n° 2004 in K and PC, n° 1604 in K), he stated that n° 2004 is the ‘type’, apparently following White’s lectotypification. Also Carpenter (1981, p. 187) examined the two exsiccata in PC (one erroneously citing as n° 2005). By overlooking White’s lectotypification and unaware of Arendholz’ study, he designated n° 1604 (PC) as lectotype.

When erecting the new species *Hy. pseudoalbidus*, Queloz et al. (2011, p. 141) examined those two duplicates of *Hy. albidus* deposited in GENT. Unaware of the previous designations, the authors designated n° 2004 in GENT as lectotype.

Of course, the two latter designations must be rejected. According to Laundon (1979), the personal herbarium of J.B.H.J. Desmazières (1786–1862) is deposited in PC. Although the Code recommends to select material as lectotype that was in the personal herbarium of the author (Recommendation 9A.4., ICBN), White’s selection of a duplicate in FH must be followed.

Desmazières’ (1850) two exsiccata appear to be part of a single collection. This can be concluded from the numbers in each of the two editions, which refer over a large range consecutively to the same taxa. Desmazières (1850, 1851) did not indicate date and location of the collection (except for ‘summer’). However, the handwritten label of Roberge’s specimen preserved at K bears the note ‘Caen’ [dépt. Calvados, France], the city where Roberge lived. Although no locality is mentioned for the type specimen, it seems probable that Roberge collected also this in the vicinity of Caen.

Husson et al. (2011) surprisingly reported more detailed specimen data: ‘collected by Roberge in 1850 from the Bois de Lébisey, close to Caen’. However, these data are actually an assumption which J.P. Rioult (director of the Départerment de Botanique, Mycologie et Biotechnologies, University of Caen) conveyed to the authors (C. Husson, personal communication) and also to us, reasoned from his personal experience about the most frequent origin of Roberge’s collections. Indeed, Roberge mostly collected in the parc de Lébisey, where he discovered his most important cryptogams (Morière 1866).

##### Misidentifications

Trustworthy records of *Hy. albidus* are actually not many. Quite a few were reported on leaves from hosts other than *Fraxinus*, such as *Acer* (Trail 1887, p. 173 [but inn Trail 1889, p. 63 changed to *Fraxinus*], FRDBI [Graddon, Spooner]), *Aesculus* (FRDBI [Crossland, Clark, Leedal]; Thind & Singh 1969; Thind et al. 1983; Sharma 1991), *Betula* (FRDBI [J.P. Blunt]), *Corylus* (FRDBI [Manvell, Shorten]), *Eucalyptus* (Beltrán Tejera et al. 2008), *Filipendula* (FRDBI [anon.]), *Platanus* (Feltgen 1903), *Quercus* (Massee & Crossland 1905, FRDBI [Hawley, Hunt]), *Robinia* (Roumeguère 1892), *Rhododendron* (FRDBI [Weir]), and *Salix* (Allescher 1898). These records probably belong to different species of *Hymenoscyphus*, or concern misidentified hosts.

##### Misidentified hosts reported for *Hymenoscyphus Albidus*

Declercq (personal communication) reported collections of *Hy. albidus* from Belgium on *Juglans* (unpreserved) and *Pyrus* (B.D. 94/108). However, re-examination of the latter collection revealed the host to be *Fraxinus*. Two unpreserved records included in our description of *Hy. albidus* were thought by the first author to be on *Acer*. Both showed asci without croziers and unquestionably concern *Hy. albidus*: in that from Luxembourg (24.IX.1990, ‘on blackened petiole of *Acer pseudoplatanus*’) druses of crystals were noted in the stipe, and in that from Tübingen (3.IX.1988, ‘on blackened primary veins of skeletonized leaf of ?*Acer*’) the spores contained large LBs. These notes on the presence of crystals or large LBs exclude *Hy. vacini*. Based on the general observation of a strict occurrence of *Hy. albidus* on *Faxinus*, we assume that the host was misidentified here.

##### Records on Fraxinus leaves misidentified as *Hymenoscyphus albidus*

Among the records on *Fraxinus*, possibly all those can be excluded in which the apothecia grew verifiably on unblackened parts of the rachises. *Hy. albidus* was actually confused in the years before ca. 1950 with *Cyathicula fraxinophila* (Svrček) Baral. According to Arendholz (1979, p. 80), almost all German records found in herbaria differ from British specimens in a textura oblita and represent at least partly a *Cyathicula. Cy. fraxinophila* resembles *Hy. albidus* in external view and is also confined to rachises (including petioles) of *Fraxinus*. Based on personal observations of the first author, this species deviates in a strongly gelatinized ectal excipulum (textura oblita) covered by rhomboid crystals, much narrower asci and spores, the former arising from croziers and with a more conical apex with an amyloid apical ring that resembles the *Calycina*-type, the latter homopolar and with a low lipid content that consists of minute LBs [spore size *(10–)14–18(–20) × (2.2–)2.5–2.8(–3) µm], also in multiguttulate paraphyses (VBs smaller and always globose), and particularly in the absence of a black stromatic tissue on the rachis and stipe base. The whitish apothecia also deviate in having a concave disc (0.6–1.2 mm diam.) with a finely crenulate margin.

Indeed, Rehm’s (1893, p. 797) description under the name *He. albidum*, with spores 15–18 × 3 µm with one oil drop at each end, undoubtedly refers to *Cy. fraxinophila*. Rehm mentioned the species from three localities: Bayern (Franken), Switzerland, and Südtirol; yet, he excluded the collection from Südtirol (leg. G. Bresadola) as deviating from the others, although he noticed here only the characteristic black stroma described by Desmazières. Rehm mentioned the Bresadola specimen also under *Helotium virgultorum* (p. 782), a lignicolous taxon which he described as growing on blackened wood surface, and he was sure that it belongs here. So only this specimen seems to represent true *Hy. albidus*.

Fautrey (in Roumeguère 1891, p. 124) described *Phialea albida* f. *microspora* Fautrey on ash rachises, with spores 10–12 × 2 µm, which again appears to concern *Cy. fraxinophila*. Also Schröter (1893, p. 74) reported under the name *Hymenoscypha albida* (Silesia, Oct.–Nov.) obviously *Cy. fraxinophila* (spores 13–18 × 2–3 µm, with homogeneous contents). Likewise Velenovský (1934, p. 205, pl. XX fig. 39) described and illustrated under the name *He. albidum* in fact *Cy. fraxinophila* (spores 12–18 × 2 µm, both ends obtuse and with granular content). Oddly enough, Velenovský confused *Cy. fraxinophila* and true *Hy. albidus*: only two of his four specimens preserved at PRM represent *Cy. fraxinophila*, according to their revision by Svrček (1985) and O. Koukol (personal communication). However, Svrček identified only one of these two (Mnichovice, XI.1933, PRM 147478) correctly (as *Conchatium fraxinophilum*), while he misidentified the other collection (Zvánovice, 24.X.1929, PRM 148804) as *Hy. albidus*, although also here the rachises were not blackened.

Without description, Le Gal (1938) reported *He. albidum* from Île-de-France (Villecresnes, Bois de la Grange et de l’Etoile) and stated the species to be very common in summer. It is possible that she was dealing in fact with *Cy. fraxinophila*. Grelet (1949, p. 35) mentioned this record, but his diagnosis is more or less a copy of Boudier’s, and it seems that Le Gal’s material has never been re-examined. Also Jaap’s (1910) report on *Fraxinus* rachises is without description; the stated association with *Cy. coronata* suggests that he was dealing with *Cy. fraxinophila*, a species which we found three times in association with *Cy. coronata*, whereas *Hy. albidus* grew always alone on the rachises. However, during survey in Czechia in 2013 *Hy. fraxineus* and *Cy. fraxinophila* were found sometimes on the same petiole (O. Koukol, personal communication). Also Gillet (1881) might have treated *Cy. fraxinophila*, because he did not mention a dark stroma.

This confusion includes also the leaf-inhabiting *Hymenoscyphus caudatus* (P. Karst.) Dennis. White (1944) stated that *Hy. albidus* is ‘known with certainty only from the material of Desmazières’, whereas he found all the many other European records under this name to be largely based upon specimens better referred to *Hy. caudatus* or *He. scutula* (Pers.) W. Phillips. This statement is in contradiction to Arendholz (1979), who mentioned only the genus *Cyathicula* as misidentification.

##### Records on leaves of *Platanus* and *Aesculus* misidentified as *Hymenoscyphus Albidus*

Feltgen (1903, p. 59) identified a collection from Luxembourg, on rotten leaves and petioles of *Platanus orientalis*, as *Phialea albida*. The small (0.2–0.5 mm diam.), yellowish, cyathiform apothecia and the narrow, obtusely fusoid spores (12–16 × 2–3.5 µm) might refer to a *Cyathicula* or *Allophylaria*. Apothecia could not be found by us in the preserved specimen (LUX 045915).Under the names *He. robergei* or *Hymenoscyphus albidus*, a taxon was reported on fallen petioles and leaf blades of *Aesculus indica* from the Northwestern Himalaya (India and Nepal), where it seems to be frequent (Thind & Singh 1969, p. 251, fig. 1; Thind et al. 1983, p. 278; Sharma 1976, p. 338, pl. 39 fig. 5–6, pl. 57 fig. 98, 1991, p. 123, pl. 4 fig. 5–6). Due to the similarity of *Hy. albidus* to both *Hy. aesculi* and *Hy. honshuanus*, which typically occur on *Aesculus*, this determination appears to be questionable and needs reinvestigation, particularly concerning the ascus base. The apothecia are described as cream to yellowish-brown when fresh, with a dark brown to black stipe base. Yet, the authors did not mention blackening of the substrate. Apart from the different substrate, Thind and Singh (1969, p. 253) noted that the Indian collections deviate from European *Hy. albidus* in narrower asci and ascospores, whereas Sharma (1976, p. 339) mentioned also ‘their more robust ascocarps’ and ‘longer ascospores’. However, the ascus and spore size given by Sharma [†(64–)75–95(–102) × 7–9.5(–11) µm, †15–22(–22.3) × 3–5 µm] fits all these species, and the fusoid-clavate spore shape would quite well match that of *Hy. honshuanus*. Those given by Thind and Singh are distinctly narrower (†85–100 × 7–7.5 µm, †16–20 × 3–3.5 µm) and the spores drawn rather cylindrical, which might better correspond to *Hy. aesculi*. However, Sharma’s remark that the apothecia become entirely dark brown to almost black on drying would fit the present observations on *Hy. aesculi.*

#### *Hymenoscyphus fraxineus* (T. Kowalski) Baral, Queloz & Hosoya, IMA Fungus 5(1): 80 (2014) – Figures 5–11

**Figures 5–6.**
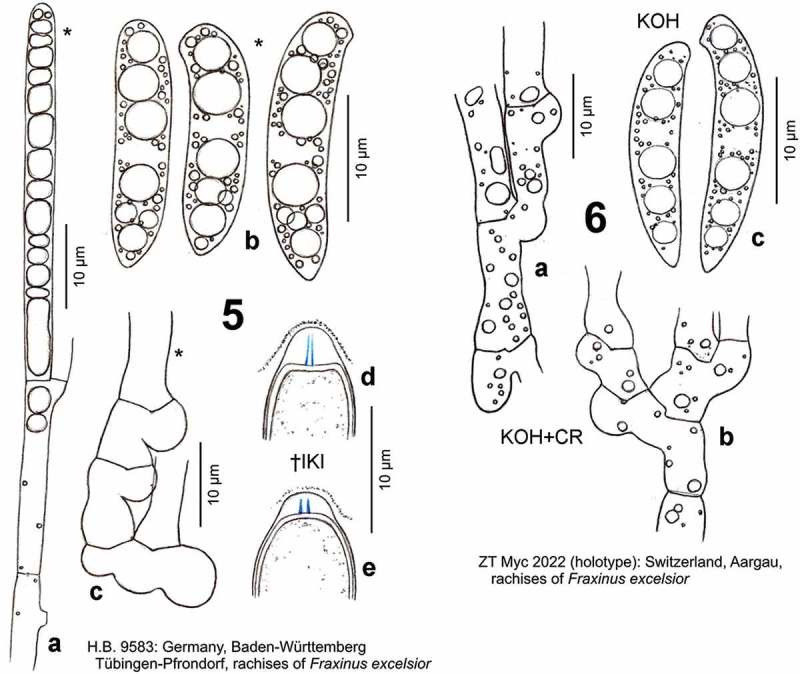
*Hymenoscyphus fraxineus*. 5a. paraphysis containing VBs; 5b. freshly ejected ascospores containing LBs, 6c. do., from inside asci; 5d–e. ascus apices with euamyloid apical ring of the *Hymenoscyphus*-type; 5c, 6a–b. ascogenous hyphae with crozier formation (in 6a–b containing LBs). – Living state: 5a–c; dead state: 5d–e, 6a–c.

**Figure 7.**
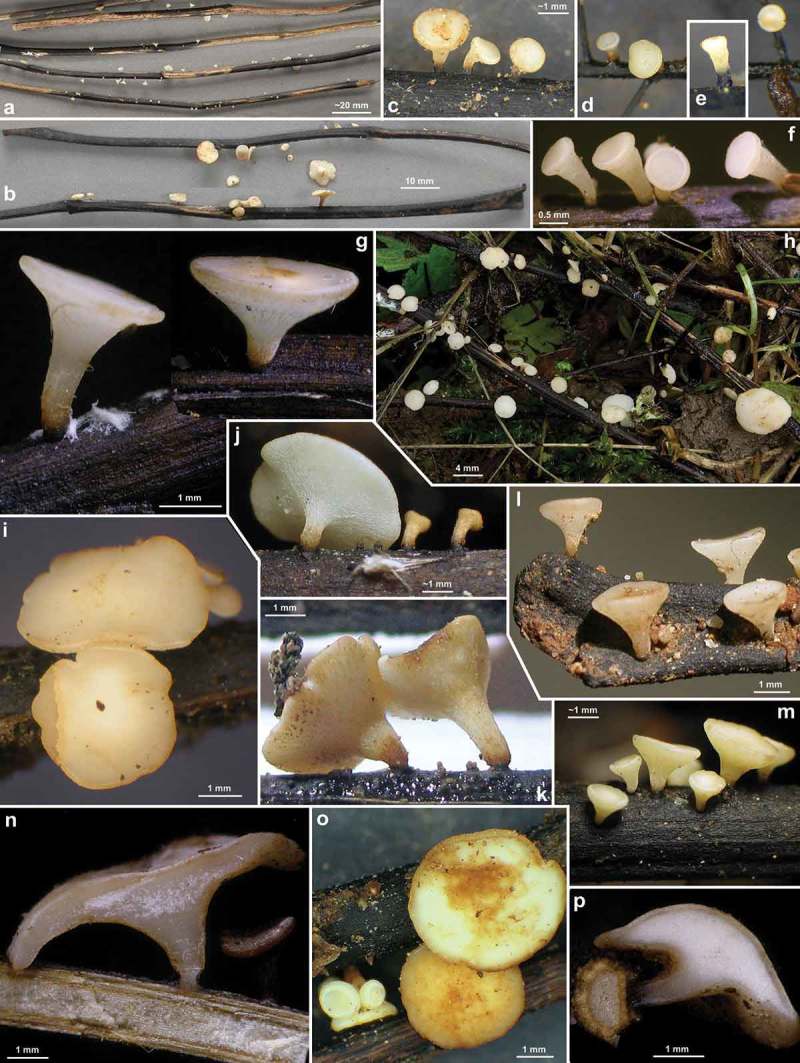
*Hymenoscyphus fraxineus* (Europe). Apothecia emerging from black pseudosclerotia in rachises and veins of *F. excelsior* (n, p: in median/longitudinal/cross section). – All in fresh state. – a–b. H.B. 9700 (DE-BW, Tübingen); c, e. 23.VI.2011 (DE-BW, Heidelberg, Ziegelhausen); d. 28.V.2011 (ibid.), f. 17.VI.2011 (Tübingen); g. H.B. 9560 (DE-BW, Reutlingen); h. 30.VI.2012 (BE-LUX, Arlon, phot. G. Marson); i. 23.VI.2012 (Heidelberg, Lobbach); j, m. 18.VII.2011 (DE-TH, Sonneberg, phot. I. Wagner); k. 15.VII.2011 (Ziegelhausen); l, n, p. H.B. 9698 (Tübingen); o. 10.VII.2011 (Heidelberg, Boxberg).

**Figure 8.**
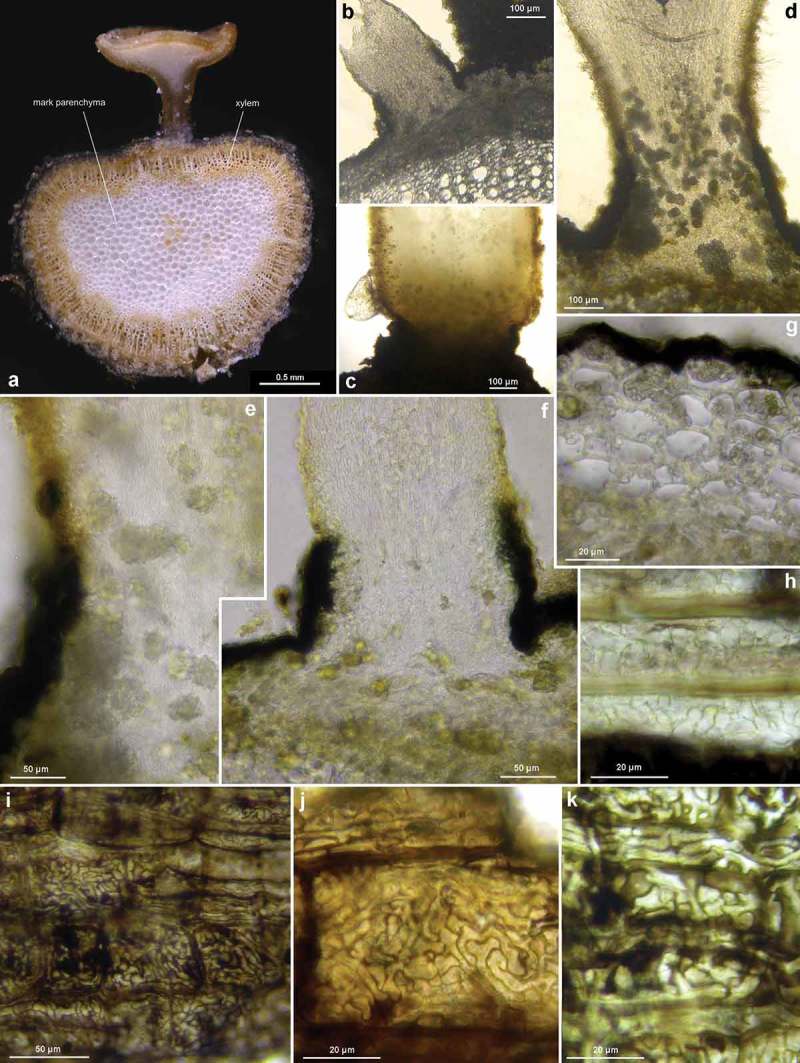
*Hymenoscyphus fraxineus* (Europe): a. apothecium in median and rachis in cross section (the black demarcation line covers the outer face of the sclerenchyma), b–f. apothecial stipe in median section (with internal crystals), g. rachis in cross section (with external demarcation line, cells of sclerenchyma containing hyphae), h–k. external view on pseudosclerotial plate (tangential section of rachis surface); cells of cortical parenchyma densely filled with hyaline or ± brown hyphae (textura epidermoidea). – All in living state. – a. H.B. 9596 (DE-BW, Tübingen), b, f–g. H.B. 9589 (DE-BY, Amberg), c–e. H.B. 9593 (DE-SN, Chemnitz), h–k. H.B. 9588 (DE-BY, Hirschau).

**Figure 9.**
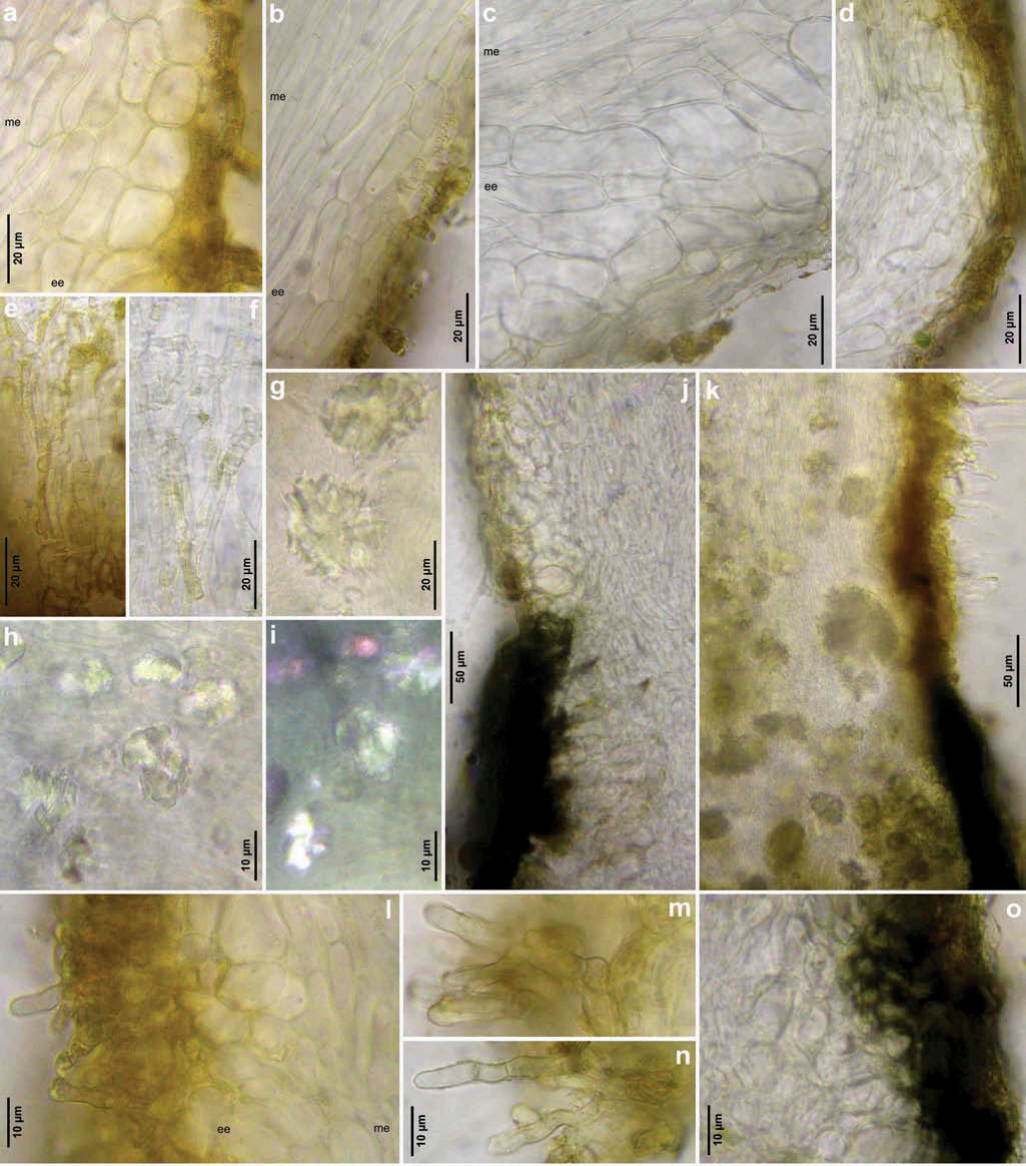
*Hymenoscyphus fraxineus* (Europe): a–b, j–o. median section of stipe, showing golden-yellow external exudate and black basal stroma (ee = ectal excipulum, me = medullary excipulum); c–d. median section of receptacle at flanks (c) and margin (d); e–f. surface view of stipe; g–i, k. crystals in medullary excipulum near stipe base; k–n. hairs at stipe base. – All in living state (i: under polarized light). – a, g, k, l–n. H.B. 9593 (DE-SN, Chemnitz); b. H.B. 9698 (DE-BW, Tübingen); c, h–i. H.B. 9700 (Tübingen); d. H.B. 9571 (CH-LU, Hitzkirch); e–f. H.B. 9583 (Tübingen); j, o. H.B. 9589 (DE-BY, Amberg).

**Figure 10.**
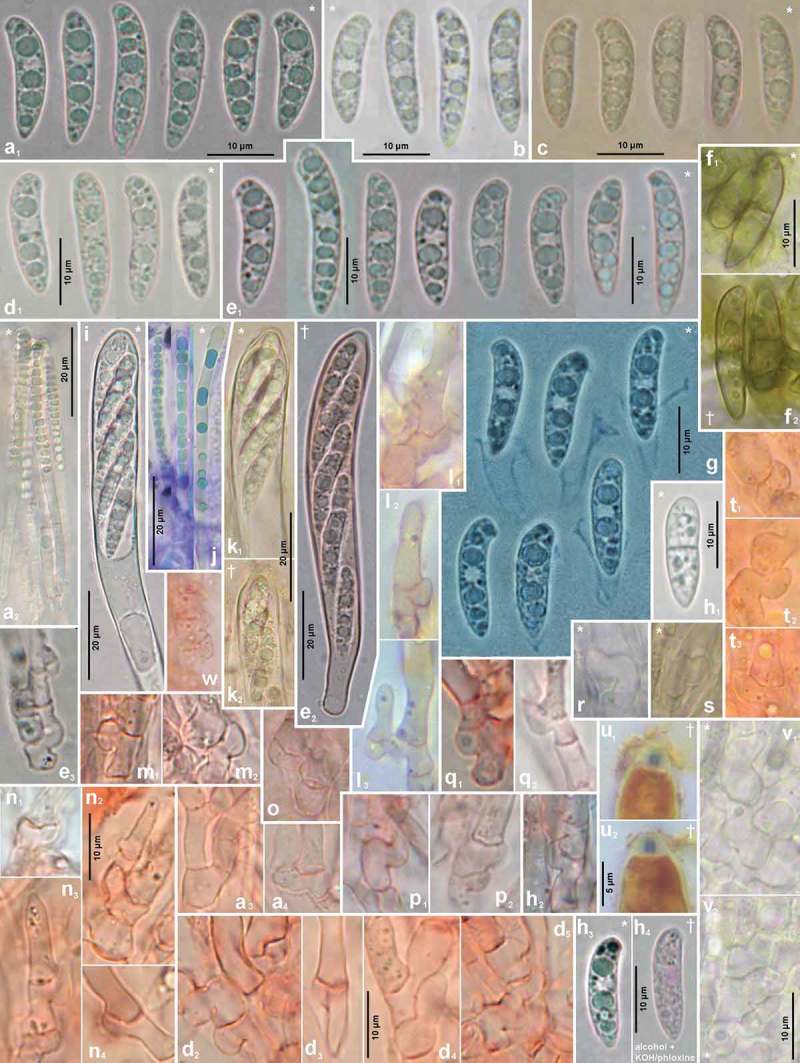
*Hymenoscyphus fraxineus* (Europe). a_1_, b, c, d_1_, e_1_, g, h_3._ ascospores (freshly ejected, containing LBs); h_4_. do. (LBs deteriorated); a_2_, j. paraphyses (containing VBs); e_2_, i, k. mature asci; f, h_1_. overmature ascospores (septate, partly with olive-brown wall); u. ascus apices with euamyloid apical ring; remaining figures: ascogenous hyphae with crozier formation. – Living state: a_1_–e_1_, a_2_, f_1,_ g (in Waterman blue-black ink), h_1_, h_3_, i, j (in CRB), k_1_, r–s, v; dead state: k_2_ (in H_2_O), a_3_–a_4_, d_2_–d_4,_ h_2_ (in CR); e_2_–e_3_, l–n, p–q (in CR_SDS_), f_2_ (in KOH), o, t (in KOH+CR), h_4_ (in ethanol + KOH + phloxine), u_1_–u_2_ (in IKI). – a. 28.V.2011 (DE-BW, Heidelberg, Ziegelhausen), b. H.B. 9572 (CH-LU, Emmenbrücke), c. H.B. 8220 (DE-ST, Chemnitz), d. 31.VII.2011 (Heidelberg, Schönbrunn), e. 10.VII.2011 (Heidelberg, Boxberg), f. H.B. 9593 (Chemnitz), g. 14.VII.2012 (Heidelberg), h. 23.VII.2011 (DE-BW, Speyer, Böhl-Iggelheim), i. 15.VII.2011 (Ziegelhausen), j. H.B. 9599 (DE-BW, Tübingen), k. H.B. 8509 (DK-Sjaelland, Sorø), l. 18.VII.2011 (DE-TH, Sonneberg), m. 2.VII.2011 (Heidelberg, Handschuhsheim), n. 5.VII.2011 (Ziegelhausen), o. ZT 3298 (CH-BE, Belp), p. 23.VI.2011 (Ziegelhausen), q. 23.VI.2012 (Heidelberg, Lobbach), r. H.B. 9698 (Tübingen), s. H.B. 9700 (Tübingen), t. ZT 3297 (CH-BE, Burgdorf), u. 8.VII.2011 (Sonneberg), v. H.B. 9583 (Tübingen). – Phot. l_1–3_, u_1–2_: I. Wagner.

**Figure 11.**
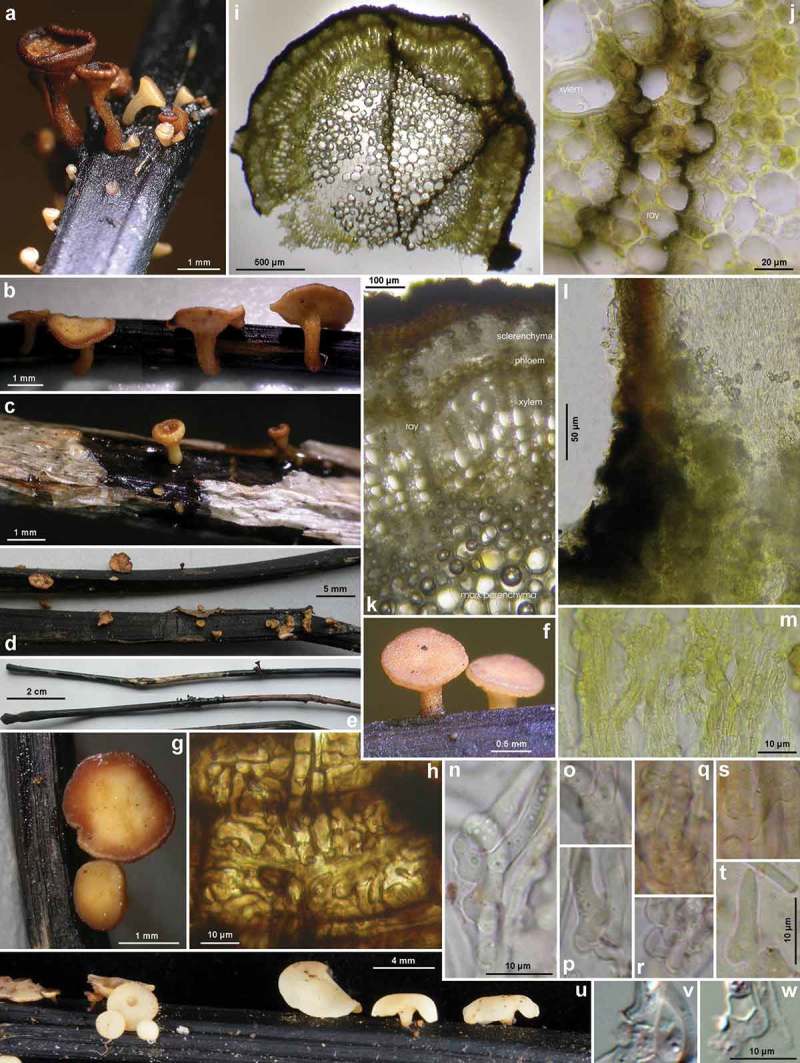
*Hymenoscyphus fraxineus* (Japan). a–g, u. Apothecia emerging from black pseudosclerotia in rachises of *Fraxinus mandshurica* (u: fresh; a–g: rehydrated); i–k. pseudosclerotium in cross section (i–j: several stromata in one rachis, delimited by black demarcation lines that occur in the xylem along radial rays and through the mark parenchyma); l. median section of stipe base (with black basal surface and internal crystals); m. surface view on ectal excipulum near margin (with yellow exudate); n–t, v–w. ascogenous hyphae with croziers. – Dead state (h–k, m in H2O; l in KOH; n–t KOH+CR; v–w in phloxine B). – Nagano: a, e, k. TNS-F-40074 (Sugadaira); b, g. TNS-F-12761 (ibid.); f, h. TNS-F-17817 (ibid.); v. TNS-F-12503 (Yuzawa); Hokkaido: c, m, s–t. TNS-F-52060 (Tomakomai); d, l, n–r, u. TNS-F-40043 (ibid.); i, j, w. TNS-F-52061 (Sapporo). – Phot. u: YJ Zhao.

≡ *Chalara fraxinea* T. Kowalski, Forest Pathology 36, p. 265 (2006) [anamorph]
*= Hymenoscyphus pseudoalbidus* Queloz, Grünig, Berndt, T. Kowalski, T.N. Sieber & Holdenr., Forest Pathology 41, p. 140 (2010) [teleomorph]

##### Etymology

*Fraxineus*: after the substrate, rachises of *Fraxinus; pseudoalbidus*: because of the morphological similarity with *Hy. albidus*.

##### Holotype of anamorph

Poland, Świętokrzyskie Voivodeship, Włoszczowa, rachises of *F. excelsior*, 6.XII.2000, T. Kowalski (Z + ZT, HMIPC 17039, living culture CBS 122504; sq.: FJ597975; d.v.).

##### Holotype of teleomorph

Switzerland, Aargau, ENE of Lenzburg, E of Othmarsingen, rachises of *F. excelsior*, 22.VII.2009, V. Queloz (ZT 2022; sq.: GU586904; Figure 6).

##### Apothecia

Moist (0.7–)1–5(–8.5) mm diam., receptacle (0.25–)0.3–0.7(–1) mm thick (at margin 0.15–0.45 mm), non-translucent, round, sometimes irregularly lobate when large, non-gelatinous; disc whitish, medium concave to flat, finally slightly convex, margin distinct, not protruding, smooth, exterior and stipe concolorous, often with fine radial pale ochraceous fibres; stipe obconical, (0.6–)1–1.5(–2) mm long, 0.15–0.4(–0.5) mm thick at the very base 0.25–0.7 mm above, 0.4–1.2 mm below receptacle, base always externally blackened, forming a ring-like zone 90–300 µm high, sharply segregated from whitish stipe above (partly by a constriction), ± erumpent from sclerenchyma-phloem layer; receptacle turning rusty brown when bruised, dry light cream, senescent ochraceous to dark brown. **Asci** *(97–)110–130(–135) × (10–)10.5–12(–12.7) µm {5}, †(80–)90–110(–116) × ((8–))(8.5–)9–10(–11)((–12)) µm {4}, 8-spored, spores *obliquely biseriate, pars sporifera *40–54 µm long, living mature asci protruding 10–25 µm beyond paraphyses; **apex** (†) conico-truncate, dome *1–1.2 µm thick, †2–3 → 1.5–1.8 µm, apical ring occupying lower 1/3–9/10 of dome, strongly blue (bb) in IKI, *Hymenoscyphus*-type {13}; **base** arising from croziers {64}. **Ascospores** *((13.5–))(15–)16–21(–22)((–24.8)) × ((3.5–))(3.7–)4–4.5(–5)((–5.5)) µm {22}, †(15–)17–19(–21.5) × (3.5–)3.7–4(–4.3) µm {2}, slightly to strongly scutuloid, cylindrical or very slightly constricted in middle part, apex rounded to obtuse, with a slight to strong lateral protrusion, base medium to strongly attenuated, straight to slightly inequilateral or medium curved, especially at the apex, containing (1–)2–3 LBs [2–3.5(–4) µm diam.] and many small ones in each half {>26}, lipid content 4.5–5, LBs fusing to one large globose drop in each half when rehydrated and still alive; sometimes a very delicate sheath was seen that slips off the spore, without setulae; overmature spores †16–21 × 4–5.7 µm {1}, 1(–2)-septate {3}, hyaline or with a smooth to rough, pale to light olivaceous-brown wall {2}. **Paraphyses** apically uninflated, terminal cell *(47–)50–70(–85) × (3–)3–4.5(–5) µm {2}, lower cells *(7–)10–20(–25) × (2.3–)2.5–3.5(–4) µm {2}; rarely to frequently branched at septa, anastomosing in lower part. **Medullary excipulum** hyaline, of a ± dense textura intricata, individual cells *(25–)28–50(–75) × 6–10(–15) µm {2}, covered by thin gel, indistinctly delimited from ectal excipulum by an up to ~100 µm thick layer of textura porrecta, in stipe sharply delimited. **Ectal excipulum** hyaline, at flanks of (*) very slightly († slightly) gelatinized textura prismatica(-porrecta), 40–80 µm thick, cells *(12–)20–50(–70) × (6–)9–20(–30) µm {5}, oriented at a (0–)10–30(–40)° angle to the surface; 10–20 µm thick at margin, oriented at 0–10°; in stipe 25–35 µm thick, of (†) slightly to medium gelatinized t. prismatica-angularis(-globulosa) to t. prismatica-porrecta oriented at 0–10°, cells *(10–)15–28(–35) × (6–)8–14(–19) µm {4}; whole exterior of receptacle and stipe covered by a 10–15 µm thick layer of cortical hyphae forming a loose to dense network in surface view, individual cells *(10–)12–30(–40) × (2.5–)4–6(–8) µm, usually forming projecting or appressed, often irregularly flexuous or curled, **hair-like protrusions** *10–30(–70) × 3.5–5(–6) µm, 1–3(–4)-celled, especially near base of stipe. **Crystals** absent in complete tissue, except for medullary tissue in lower region of stipe at a length of ~100–400(–1000) µm, sparse to abundant, ±rhomboid, usually forming druses 10–30(–80) µm diam. **VBs** in paraphyses strongly refractive {>15}, hyaline, globose to angular (multiguttulate), later very elongate, occupying upper (32–)40–70(–75) µm; cortical hyphae and hairs on receptacle rarely or often containing globose, low- to medium-, sometimes high-refractive globose VBs. **Exudate** pale to bright yellowish-ochraceous, smooth to rough, covering cortical hyphae of entire receptacle and especially stipe. **IKI**: complete tissue inamyloid, VBs in paraphyses light to bright yellow- to red-brown; **CRB**: spore wall unstained, gel in ectal and medullary excipulum deep lilac, VBs in paraphyses slowly staining bright turquoise-blue.

##### Habitat

*Aceri-Fraxinetum, Pruno-Fraxinetum*, etc., on fallen previous year’s blackened rachises {51} or sometimes primary and secondary veins {4}, rarely on a 5.5 mm thick decorticated blackened twig {1}, of *F. excelsior* {53}, *F. excelsior* f. *pendula* {1}, *F. mandshurica* {9}, *Fraxinus rhynchophylla* {9}, lying on ground. **Assoc.**: *Pyrenopeziza petiolaris* {1}. **Desiccation tolerance**: intolerant for most elements (ascogenous hyphae survived a few hours on the dry slide), except for the ascospores, many of which were either viable after 6 months in the herbarium, or they were all dead after 4 months. **Phenology**: (V–)VI–IX. **Altitude**: 30–1600 m (Europe), 23–1325 m (E-Asia).

##### Specimens included

On rachises *F. excelsior*, if not otherwise stated, in Japan on rachises of *F. mandshurica*, in South Korea on rachises of *F. rhynchophylla* or *F. mandshurica*; list incomplete concerning records not examined by us.

**Poland**: **Świętokrzyskie Voivodeship**, Włoszczowa, 6.XII.2000 T. Kowalski (conidial isolate, **holotype** of *Ch. fraxinea*, in Z + ZT, living culture HMIPC 17039 in CBS 122504, sq.: FJ597975). – **Lesser Poland Voivodeship**, 18 km NNE of Kraków, 0.6 km S of Wesoła, 290 m, 24.VIII.2008, T. Kowalski (HMIPC 20.08, H.B. 9013; HMIPC 19.08, sq.: FJ597977). – **Denmark**: **Sjaelland**, S of Sorø, 4 km S of Frederiksberg, Suserup Skov, 30 m, 25.V.2007, H.O. Baral (H.B. 8509ø). – **Belgium**: **Luxembourg**, 2.7 km NNE of Arlon, NNE of Frassem, 368 m, 30.VI.2012, G. Marson (G.M. 2012–06-30 #1, d.v.). – **Luxembourg**: **Oesling**, 7 km W of Wiltz, Doncols, rue de village 31, 465 m, on rachises of *F. excelsior* f. *pendula*, 24.VIII.2012, M.T. Tholl (ø, d.v.). – **Gutland**, 5 km SW of Luxembourg, NE of Leudelange, Bois de Cessange, 290 m, 16.VI.2012, G. Marson (ø, d.v.). – **Germany**: **Mecklenburg-Vorpommern**, Lübeck, SW of Rehna, 3 km ENE of Dechow, Staatsforst Rehna, S of forester’s lodge, 63 m, 17.VI.2007, T. Richter (H.B. 8551). – **Nordrhein-Westfalen**, 10 km NE of Gelsenkirchen, E of Herten, Waldfriedhof, 55 m, 27.VII.2011, F. Kasparek (ø, d.v.). – 6 km SE of Gescher, S of Kuhlenvenn, 75 m, 15.VIII.2011, K. Siepe (ø, n.v.). – **Sachsen**, 9 km ESE of Chemnitz, W of Erdmannsdorf, Edelmannsbachtal, 375 m, 23.VI.2010, B. Mühler (ø). – 4.5 km NE of Chemnitz, Zeisigwald, 330 m, 10.VII.2006, B. Mühler (H.B. 8220, sq.). – ibid., 370 m, 30.VIII.2011, B. Mühler (H.B. 9593). – **Thüringen**, 5 km ESE of Sonneberg, Föritz, Gasthof Steiner, 412 m, 18.VII.2011, I. Wagner (ø, d.v.). – 1.4 km NW of Föritz, 411 m, 14.VIII.2013, I. Wagner (ø, d.v.). – 2.3 km SSE of Sonneberg, E of Oberlind, Agroprodukt, 365 m, partly on veins, 8.VII.2011, I. Wagner (ø, d.v.). – 9 km NE of Gera, 2 km WNW of Köstritz, W of Gleina, 230 m, 24.VI.2011, I. Wagner (ø, d.v.). – **Baden-Württemberg**, 7 km E of **Heidelberg**, ESE of Ziegelhausen, Bärenbach, 195 m, 28.V.2011, M. Bemmann (ø). – ibid., 2.VI.2011, M. Bemmann (ø). – ibid., 23.VI.2011, M. Bemmann (M.B. 1/2011). – ibid., 175 m, 5.VII.2011, M. Bemmann (M.B. 3/2011). – 3.5 km ENE of Heidelberg, W of Ziegelhausen, Haarlaß, 114 m, 15.VII.2011, M. Bemmann (ø). – 3.5 km NE of Heidelberg, E of Handschuhsheim, Siebenmühlental, above Turnerbrunnen, 222 m, 2.VII.2011, M. Bemmann (M.B. 2/2011). – 3 km SE of Heidelberg, Boxberg, 280 m, 10.VII.2011, M. Bemmann (M.B. 04/2011). – 14.5 km ESE of Heidelberg, NW of Lobbach-Waldwimmersbach, 240 m, 23.VI.2012, M. Bemmann (ø). – 3.5 km E of Heidelberg, Königstuhl, Landesssternwarte, 572 m, 14.VII.2012, M. Bemmann (ø). – 7.5 km WNW of Speyer, 3 km SE of Böhl-Iggelheim, 116 m, 23.VII.2011, M. Bemmann (M.B. 05/2011). – 7 km SSW of Eberbach, ESE of Schönbrunn, Todtenbronnen, 362 m, 31.VII.2011, M. Bemmann (M.B. 06/2011). – 7.3 km NE of Karlsruhe, Füllbruch, 122 m, 5.VI.2011, D. Bandini (KR-M-0027298). – 6 km NE of **Tübingen**, E of Pfrondorf, Tiefenbach, 410 m, partly on veins, 17.VI.2011, H.O. Baral (ø). – ibid., 10.VII.2012 (H.B. 9700ø). – ibid., 29.VIII.2012 (H.B. 9721ø). – ibid., 385 m, 31.VII.2011 (H.B. 9583). – ibid., partly on veins, 24.IX.2011 (H.B. 9602ø). – ibid., 2.VII.2012 (H.B. 9698ø). – ibid., 27.VIII.2013 (H.B. 9836ø). – S of Pfrondorf, Haldenbach, 385 m, 7.IX.2011, H.O. Baral (H.B. 9596ø). – 1.7 km N of Pfrondorf, Zeitungseiche, 460 m, 9.IX.2011, H.O. Baral (H.B. 9599ø). – Reutlingen, Lederstraße, Echaz, 390 m, 25.VI.2011, H.O. Baral (H.B. 9560ø). – **Bayern**, 0.5 km SSE of Amberg, Vils, 370 m, 17.VIII.2011, H.O. Baral (H.B. 9589ø). – 1 km W of Hirschau, Moosweiher, 415 m, 19.VIII.2011, H.O. Baral (H.B. 9588ø). – 10.5 km SW of Starnberg, 2 km NW of Tutzing, Deixlfurter See, 700 m, 13.VIII.2006, B. Fellmann (ø, #). – 21 km S of München, NW of Eulenschwang, 685 m, 19.VII.2011, B. Fellmann (ø). ― **Austria** **, Niederösterreich**, 8 km NW of Wiener Neustadt, SW of Wöllersdorf-Marchgraben, 380 m, 18.VII.2006, G. Koller (G.K., H.B. 9893ø). – **Burgenland**, 12 km SW of Mariazell, 1.5 km ESE of Dürradmer, 865 m, 30 VII.2006, G. Koller (G.K.). – **Switzerland**: **Graubünden**, 4.5 km ENE of Landquart, Crupspitz, 1120 m, 14.VIII.2014, H.O. Baral (H.B. 9907ø). – **Solothurn**, 1.7 km N of Solothurn, SE of Brüggemoos, Verenaschlucht, 510 m, 18.VII.2009, P. Zaffarano (ZT 3292, sq.: GU586911, 586912, 586913). – **Jura**, 1.1 km NW of Porrentruy, WNW of Waldegg, 508 m, 5.VII.2009, V. Queloz (ZT 3301, sq.: GU586907, 586908, 586909). – **Fribourg**, 8.2 km NNW of Payerne, NE of Chevroux, la Grande Cariçaie, 458 m, 27.V.2011, R. Dougoud & F. Ayer (ZT, ex R.D. 32.16.094.11, d.v.). – **Bern**, 5.5 km W of Burgdorf, NE of Hindelbank-Neufeld, Hurst, 530 m, 17.VII.2009, B. Senn-Irlet (ZT 3297, sq.: GU586906). – 3 km ESE of Belp, Leen, Belpau, 530 m, 18.VII.2009, B. Senn-Irlet (ZT 3298, sq. GU 586905). – **Aargau**, 4.5 km ENE of Lenzburg, E of Othmarsingen, 500 m, 22.VII.2009, V. Queloz (ZT 2022, **holotype** of *Hy. pseudoalbidus*, H.B. 9453ø, sq.: GU586904). – **Zug**, 3 km SSW of Unterägeri, E of Ochsenfeissi, Haslen, 835 m, 23.VI.2011, U. Graf (NMLU). – **Luzern**, 16 km NNW of Luzern, 3.3 km SW of Hitzkirch, Erlosen am Baldeggersee, Gmeinwald, 580 m, partly on a twig, 13.VII.2011, U. Graf (H.B. 9571). – 2 km NW of Luzern, SE of Emmenbrücke, at river Reuss, 435 m, 14.VII.2011 (H.B. 9572ø). – 2.8 km NE of Luzern, 0.4 km N of Dietschiberg, Hombrig, 585 m, 2.VII.2011, U. Graf (ø, n.v.). – **France**: **Champagne-Ardenne, Haute-Marne**, 50 km NNE of Dijon, E of Villiers-lès-Aprey, Fontaine aux Dames, 380 m, 17.VI.2012, A. Gardiennet (A.G. 12080, d.v.). – **Japan**: **Hokkaido**, 12 km WSW of Tomakomai-shi, Nishikionuma Park, 23 m, 12.IX.2011, Y.J. Zhao (TNS-F-40043, H.B. 9649ø, sq.: AB705219). – ~5 km N of Tomakomai-shi, Experimental Forest of Hokkaido University, VII.1990, T. Hosoya (TNS-F-52060, H.B. 9652ø). – 18.5 km WSW of Chitose-shi, Shikotsu lake, Hukkonomori, 240 m, 14.IX.2011, Y.J. Zhao (TNS-F-40051, H.B. 9650ø, sq.: AB705220). – 50 km ESE of Sapporo, ~15 km S of Yubari-shi, Taki-no-ue Park, ~200 m, VII.1990, T. Hosoya (TNS-F-52061, H.B. 9657ø). – **Honshu, Nagano**, Chiisagata-gun, Sanada-machi, 16.5 km NE of Ueda, SE of Sugadaira, Sugadaira Montane Center, Arboretum, 1325 m, VIII.1992, T. Hosoya (TNS-F-52062, H.B. 9651ø). – ibid., 7.IX.2005, T. Shirouzu (TNS-F-17817, H.B. 9734ø, sq.: AB705224). – ibid., 11.IX.2006, T. Hosoya (TNS-F-12761, H.B. 9659ø, sq.: AB705218). – ibid., 28.IX.2011, Y.J. Zhao (TNS-F-40074, H.B. 9658ø, sq.). – 43 km SE of Joetsu, 20 km WSW of Yuzawa (Niigata), Daimyojin, 990 m, 10.IX.2006, T. Hosoya (TNS-F-12503, d.v., sq.: AB705221). – **South Korea**: 26 km NNW of Pyeongchang, 28 km ENE of Hoengseong, Mt. Taegi, 1043 m, rachises of *F. rhynchophylla*, 14.VIII.2008, J.G. Han (KUS-F52255, d.v., sq.: KF830850). – 9 km WNW of Pyeongchang, 29 KM ESE of Hoengseong, Supchaewon, 1100 m, veins of *F. mandshurica*, 23.IX.2008, J.G. Han (KUS-F52355, d.v., sq.: KF830851). – Further eight records made on *F. rhynchophylla* during 2007–2010, 14.VII.–27.VIII. – **Russia (far east)**: **Primorsky**, 30 km E of Wladiwostok, 2.7 km E of Primosrky, Kedrovaya Pad, 60 m, rachises of *F. mandshurica*, 17.VIII.2005, E. Popov (LE 294377, n.v.). – **China**: **Jilin**, 64 km E of Jilin, 8 km N of Jiaohe, Lafashan,, 290 m, rachises of *F. mandshurica*, 22.VII.2012, T. Bau, W.Y. Zhuang, H.D. Zheng, Z.Q. Zeng, Z.X. Zhu & F. Ren (HMAS 264152, n.v.). – 93 km ENE of Jilin, 39 km NE of Jiaohe, Qianjin, 395 m, rachises and veins of *F. mandshurica*, 23./24.VII.2012, T. Bau et al. (HMAS 264174, 266596, 266580, 266581, d.v.). – 225 km SE of Jilin, 34 km S of Dunha, 15 km NNE of Changbaishan, 1310 m, rachises of *F. mandshurica*, 27.VII.2012, T. Bau et al. (HMAS 266582, 266583, n.v.) [coordinates and altitude in Zheng and Zhuang (2014) incorrect for two sites].

##### Characterization

*Hy. fraxineus* differs from *Hy. albidus* mainly in the presence of croziers. Further features comprise a tendency to apothecia larger (up to 8 mm diam.), larger pseudosclerotia (often occupying the almost entire rachis), slightly larger ascospores, an earlier start of fructification, and the presence of an anamorph in pure culture.

##### History about the detection of *Hymenoscyphus fraxineus*

Judging from the list of specimens examined by Queloz et al. (2011), *Hy. fraxineus* was genetically recorded in Europe since 2000, with two exceptions dating back to 1978 and 1987 which later turned out to concern *Hy. albidus* (see below). Although the ash dieback disease was first observed in Northern Poland in 1992, the oldest isolate of the anamorph state dates from 6.XII.2000, which represents the type culture of *Ch. fraxinea* (Kowalski 2006). Apothecia of *Hy. fraxineus* were first discovered in 2006 and 2007 in Germany (Sachsen and Mecklenburg-Vorpommern, see Table 2), in 2008 in Poland (Kowalski & Holdenrieder 2009), and from June 2009 onwards in Switzerland by Queloz et al. (2011). Since then, *Hy. fraxineus* became an extremely frequent species that shows mass fructifications throughout Central Europe every year, forming 370.000 up to 13.350.000 apothecia per hectare at a time (Kowalski et al. 2013), and totally replacing *Hy. albidus* in these regions. During invasion of Switzerland, *Hy. fraxineus* did not surpass an altitude of 850 m while *Hy. albidus* was still present at higher altitudes (up to 1690 m), according to data in Queloz et al. (2011). However, there is evidence that both species exhibit a similar tolerance to low temperatures. Apothecia of *Hy. fraxineus* were presently recorded in the Alps at 1410 m and in Japan at 1325 m as a maximum, and disease symptoms were seen in the Alps up to 1600 m.

When randomly checking fresh collections of *Hy. albidus* (sensu lato), the first author observed differences in ascus development among the studied samples: apothecia found during 1988–1991 in the south of Germany (Tübingen) and in Liechtenstein possessed asci arising from simple septa, whereas in those found during 2006–2010 in Sachsen (Chemnitz), Mecklenburg (Rehna), and Denmark (Sjælland) the asci consistently arose from croziers. Because there were no other deviating features to be found in the 2006–2010 specimens, the observed deviation was first thought to represent variation within a single species. When ash disease invaded Europe and the *Hy. albidus* sexual state was identified as the causal agent, the first author became suspicious that the two variants of *Hy. albidus* that he had observed, with differing types of ascus development, were the two ‘cryptic’ species reported by molecular methods.

In the present study, 13 sequenced specimens from ZT and 1 from HMIPC, which were identified by Queloz et al. (2011) based on their rDNA sequences, were re-examined for the ascus base. Further four specimens were sent to V. Queloz by the first author to be sequenced. The result confirms the above hypothesis: *Hy. fraxineus* can be distinguished from *Hy. albidus* by the presence of croziers: six out of seven collections identified by molecular methods as *Hy. pseudoalbidus* (=*Hy. fraxineus*) turned out to have croziers, whereas seven collections identified as *Hy. albidus* were found to have simple-septate ascus bases (Tables 1 and 2, HMIPC, ZT).

**Table 1.** Records of *Hymenoscyphus albidus* for which the ascus base was tested {29} and partly also the ITS sequence obtained {12}.

Voucher	Location	alt.	cr.	test	ps.	DNA	diam.	coll. date
S.Å.H. 15290	S-Skåne, Helsingborg	42	–	1^a^		–	–	24.VIII.1994
B.D. 94/092	BE-VOV Sinaai	5	–	1^b^		–	0.8–3.5	16.VII.1994
B.D. 94/108, H.B. 9847	BE-VOV Brugge	2	–	1	m	–	1–1.5	24.VIII.1994
B.D. 89/076	BE-WLX Virton	250	–	1^b^		–	1–2.5	13.VIII.1989
LUX 047702, H.B. 9611ø	BE-WLX Orval	~200	–	1	m	–	~0.7–1.5	~5.IX.1990
S.H. 444, H.B. 9854	NL-GL Nijmegen	8	–	1/1^c^	i	–	1–1.8	7.VII.2007
LUX 047701, H.B. 9612ø	LU-Echternach	~200	–	1	i/m	–	–	14.VII.1985
H.B. ø	LU-Mertert (on ‘*Acer*’)	155	–	1		–	–	24.IX.1990
LUX 047699, H.B. 9619ø	LU-Kehlen	370	–	1	i	–	~0.7–2	17.VII.1988
LUX 047700, H.B. 9620ø	LU-Manternach	~200	–	2	i/m	–	~0.7–2	15.VIII.1985
H.B. 3841	DE-RP Pirmasens	280	–	2	i/m	alb.^+^	0.8–2.7	~3.IX.1989
H.B. ø	DE-BW Tübingen	400	–	1		–	–	14.IX.1991
H.B. 1950	DE-BW Stockach	550	–	2	i/m	alb.^+^	0.9–1.8	20.VIII.1976
H.B. 5834	LI-Bendern	445	–	2	i/m	alb.^+^	1.5–4	5.VII.1997
ZT (R.D. 32.28.094.11ø)	CH-FR Boulogne	820	–	2^d^		–	–	14.VIII.2011
ZT (R.D. 32.29.094.11ø)	CH-FR Mothey	800	–	1^d^		–	–	14.VIII.2011
ZT 3300	CH-BE Eymatt	508	–	2		alb.	1–2.5	5.VIII.2009
ZT 3299	CH-BE Eymatt	530	–	2		alb.	1–2	5.VIII.2009
ZT, H.B. 9454ø	CH-LU Aesch	530	–	2	i/m	alb.†	1–2.5	4.IX.1978
ZT (ex NMLU)	CH-ZG Unterägeri	830	–	1		alb.	1–2	21.VII.2007
ZT 3296	CH-TI Quinto	990	–	2		alb.	1–1.5	26.VII.2009
ZT 3295	CH-TI Aquarossa	597	–	1		alb.	1–2	28.VII.2009
ZT 3294	CH-TI Lavorgo	616	–	1		alb.	1–1.7	27.VII.2009
Desm. 1604, 2004 (type)	FR-BN Caen	65	–	2^e^	i/m	alb.*	1–2	summer ca. 1850
ZT 3293, R.D. 22.11.094.95ø	FR-BN Bellême	190	–	1		alb.	0.8–2.5	28.IX.1995
M.H. 220607ø	FR-PC Niort, Granzay	25	–	1^f^	(i)/m	–	–	16.VI.2007
H.B. 9699, M.H. 10712	FR-PC Niort, Granzay	25	–	1	i/m	–	1.3–3.8	2.VII.2012
ARAN-Fungi 000082	ES-VA Gipuzkoa, Aia	295	–	1^g^	i/m	–	1–3	25.VII.2014
M.B. 10/2014	ES-VA Atxondogoikoa	600	–	1	m	–	1–3	18.VIII.2014

Notes: Explanations: alt. = altitude [m]; cr. = croziers; test = number of apothecia tested for croziers, examined by ^a^S.Å. Hanson & T. Laessøe, ^b^B. Declercq, ^c^S. Helleman, ^d^R. Dougoud, ^e^White (1944), ^f^M. Hairaud, ^g^I. Olariaga; ps. = pseudosclerotium (i = isolated patches, m = major part of rachis blackened); DNA = molecular identification: alb. = identified as *Hy. albidus* by Queloz et al. (2011); alb.^+^ = confirmed as *Hy. albidus* by V. Queloz (personal communication); alb* = sequenced by Husson et al. (2011); alb.† = at first misidentified as *Hy. pseudoalbidus* by Queloz et al. (2011), but later resequenced and corrected in GenBank by Queloz et al. (2012); diam. = apothecial diameter in fresh state [mm].

**Table 2.** Records of *Hymenoscyphus fraxineus* for which the ascus base was tested {73} and partly also the ITS sequence obtained {16}.

Voucher	Location	alt.	cr.	Test	ps.	DNA	diam.	coll. date
HMIPC 20.08, H.B.9013	PL-Kraków, Wesoła	290	+	1	m	frx.	1–2.5	24.VIII.2008
BILAS 25151	LT-Kėdainiai, Kėdainiai	63	+	1^a^	m	–	~1–4	3.IX.2001
BILAS 33994	LT-Kėdainiai, Kėdainiai	50	+	2^a^	m	–	~1–4	3.X.2002
BILAS 34422	LT-Kėdainiai, Kėdainiai	58	+	1^a^	m	–	~1–4	21.IX.1999
BILAS 34425	LT-Kėdainiai, Kėdainiai	63	+	1^a^	m	–	~1–4	2.X.2002
BILAS 34426	LT-Kėdainiai, Kėdainiai	52	+	1^a^	m	–	~1–4	21.IX.1999
WI 4714	LT-Vilnius, Vilnius	130	+	1^a^	m	–	~1–4	25.VI.2001
H.B. 8509ø	DK-Sjaelland, Suserup	30	+	1^a^		–	–	25.V.2007
G.M. 2012–06-30 #1	BE-WLX Arlon, Frassem	368	+	1^b^	i/m	–	1–8	30.VI.2012
ø	LU-Doncols	465	+	1^c^		–	3–5	24.VIII.2012
ø	LU-Leudelange	290	+	1^b^		–	0.5–5	16.VI.2012
H.B. 8551	DE-MV Rehna, Dechow	63	+	1	m	–	1.5–5	17.VI.2007
ø	DE-NW Gelsenkirchen, Herten	55	+	1^d^	m	–	–	27.VII.2011
ø	DE-NW Gescher	75	+	1^e^		–	2–4	15.VIII.2011
ø	DE-SN Chemnitz, Erdmannsdorf	375	+	1	m	–	–	23.VI.2010
H.B. 8220	DE-SN Chemnitz, Zeisigwald	330	+	2	m.	frx.	1–5	10.VII.2006
H.B. 9593	DE-SN Chemnitz, Zeisigwald	370	+	2	m	–	2–6	30.VIII.2011
ø	DE-TH Köstritz, Gleina	230	+	~1^f^	m	–	–	24.VI.2011
ø	DE-TH Sonneberg, Unterlind	350	+	~1^f^	m	–	–	8.VII.2011
ø	DE-TH Sonneberg, Föritz	412	+	~4^f^	m	–	–	18.VII.2011
ø	DE-TH Sonneberg, Föritz	411	+	~1^f^	m	–	0.8–5	14.VIII.2013
KR-M-0027298	DE-BW Karlsruhe, Füllbruch	120	+	1	i	–	_	5.VI.2011
ø	DE-BW Heidelberg, Bärenbach	195	+	1	m	–	–	28.V.2011
ø	DE-BW Heidelberg, Bärenbach	195	+	1	m	–	2–5	2.VI.2011
M.B. 1/2011	DE-BW Heidelberg, Bärenbach	195	+	3	m	–	1–4	23.VI.2011
M.B. 3/2011	DE-BW Heidelberg, Bärenbach	175	+	1	m	–	2–7.5	5.VII.2011
ø	DE-BW Heidelberg, Haarlaß	114	+	1	m	–	1–5	15.VII.2011
M.B. 2/2011	DE-BW Heidelberg, Handschuhsh.	222	+	2	m	–	1.5–5	2.VII.2011
M.B. 04/2011	DE-BW Heidelberg, Boxberg	280	+	1	m	–	1.5–4.5	10.VII.2011
M.B. 05/2011	DE-BW Speyer, Böhl-Iggelheim	116	+	1	m	–	2–6.6	23.VII.2011
M.B. 06/2011	DE-BW Eberbach	362	+	2	i/m	–	2–8.5	31.VII.2011
ø	DE-BW Heidelberg, Lobbach	240	+	1	m	–	1–4.5	23.VI.2012
ø	DE-BW Heidelberg, Königstuhl	572	+	1		–	–7	14.VII.2012
ø	DE-BW Tübingen, Tiefenbach	410	+	2	i/m	–	0.7–1.8	17.VI.2011
H.B. 9700ø	DE-BW Tübingen, Tiefenbach	410	+	4	i/m	–	1–6.5	10.VII.2012
H.B. 9721ø	DE-BW Tübingen, Tiefenbach	410	+	1	i/m	–	0.7–2	29.VIII.2012
H.B. 9583	DE-BW Tübingen, Tiefenbach	385	+	3	m	–	1.3–5.5	31.VII.2011
H.B. 9602ø	DE-BW Tübingen, Tiefenbach	385	+	2	i/m	–	0.4–1.6	24.IX.2011
H.B. 9698ø	DE-BW Tübingen, Tiefenbach	380	+	3	m	–	1–8	2.VII.2012
H.B. 9836ø	DE-BW Tübingen, Tiefenbach	380	+	1	m	–	1–4.5	27.VIII.2013
H.B. 9596ø	DE-BW Tübingen, Haldenbach	385	+	4	i/m	–	1–1.5	7.IX.2011
H.B. 9599ø	DE-BW Tübingen, Zeitungseiche	460	+	1		–	1–1.5	9.IX.2011
H.B. 9560ø	DE-BW Reutlingen	390	+	2	m	–	1–3	25.VI.2011
H.B. 9589ø	DE-BY Amberg	370	+	1		–	1.5–1.7	17.VIII.2011
H.B. 9588	DE-BY Hirschau	415	+	3		–	1–3	19.VIII.2011
ø	DE-BY München, Eulenschwang	685	+	3	m	–	0.8–4.2	19.VII.2011
G.K., H.B. 9893ø	AT-NÖ W.-Neustadt, Wöllersdorf	380	+	1	m	–	1.5–3	18.VII.2006
G.K.	AT-BL Mariazell, Dürradmer	865	+	1	m	–	1.5–3	30.VII.2006
H.B. 9907ø	CH-GB Landquart	1120	+	1	m	–	1–2	14.VIII.2014
ZT 3292	CH-SO Brüggemoos	510	+	2		frx.	1–2.5	18.VII.2009
ZT 3301	CH-JU Porrentruy	508	+	1	m	frx.	1–4	5.VII.2009
ZT (R.D. 32.16.094.11ø)	CH-FR Payerne	458	+	1^g^		–	–	27.V.2011
ZT 3297	CH-BE Burgdorf	530	+	2		frx.	2–4	17.VII.2009
ZT 3298	CH-BE Belp	530	+	2		frx.	1.5–4.5	18.VII.2009
ZT 2022 type, H.B.9453ø	CH-AG Lenzburg	500	+	2	m	frx.	1–5.5	22.VII.2009
NMLU	CH-ZG Unterägeri	835	+	1^h^		–	–	23.VI.2011
H.B. 9571	CH-LU Hitzkirch	580	+	3	m	–	3–6	13.VII.2011
H.B. 9572ø	CH-LU Emmenbrücke	435	+	2		–	2–7	14.VII.2011
ø	CH-LU Dietschiberg	585	+	1		–	–	2.VII.2011
ø	FR-CA Dijon	380	+	1^i^	m	–	–	17.VI.2012
HMAS 264174	CN-Jilin Qianjin	395	+	1 ^l^	m	frx.	0.8–2	23.VII.2012
LE 294377	RU-Wladiwostok, Primorsky	60	+	1 ^m^		–	–	17.VIII.2005
TNS-F-40043, H.B.9649ø	JP-Hokkaido, Tomakomai-shi	23	+	1	m	frx.	1–4	12.IX.2011
TNS-F-52060, H.B.9652ø	JP-Hokkaido, Tomakomai-shi	23	+	1	(m)	–	0.9	VII.1990
TNS-F-40051, H.B.9650ø	JP-Hokkaido, Chitose-shi	240	+	1	(m)	frx.	1.5–2.2	14.IX.2011
TNS-F-52061, H.B.9657ø	JP-Hokkaido, Yubari-shi	~200	+	1/1^j^	i	–	1	VII.1990
TNS-F-52062, H.B.9651ø	JP-Nagano, Sugadaira	1325	+	1	i/m	–	0.8–1.8	VIII.1992
TNS-F-17817, H.B.9734ø	JP-Nagano, Sugadaira	1325	+	1	m	frx.	1–2.7	7.IX.2005
TNS-F-12761, H.B.9659ø	JP-Nagano, Sugadaira	1325	+	1	i/m	frx.	1.1–2.3	11.IX.2006
TNS-F-40074, H.B.9658ø	JP-Nagano, Sugadaira	1325	+	1	i/m	frx.	0.5–2.3	28.IX.2011
TNS-F-12503	JP-Nagano, Yuzawa	990	+	1^j^		frx.	–	10.IX.2006
KOH-F-52255	KR-Pyeongchang, Mt. Taegi	1043	+	1^k^		frx.	?–2	14.VIII.2008
KUS-F-52355	KR-Pyeongchang, Supchaewon	1100	?	–	m	frx.	?–2	23.IX.2008

Notes: Explanation: alt. = altitude [m]; cr. = croziers; test = number of apothecia tested for croziers, examined by ^a^E. Kutorga, ^b^G. Marson, ^c^M.T. Tholl,^d^F. Kasparek, ^e^K. Siepe, ^f^I. Wagner, ^g^R. Dougoud, ^h^U. Graf, ^i^A. Gardiennet, ^j^Zhao et al. (2013), ^k^J.G. Han, ^l^ Zheng and Zhuang (2014), ^m^ E. Popov; ps. = pseudosclerotium (i = isolated patches, m = major part of rachis or vein blackened); DNA = molecular identification (frx. = identified as *Hy. fraxineus* [*Hy. pseudoalbidus*] by Queloz et al. 2011 [PL, CH], Zhao et al. 2013 [JP], or J.G. Han [KR]; frx.^+^ = confirmed as *Hy. fraxineus* by V. Queloz personal communication); diam. = apothecial diameter in fresh state [mm].

However, the result for one of those specimens identified by molecular data as *Hy. pseudoalbidus* (Luzern, Aesch, collected in 1978, NMLU 0409–78) behaved contradictory: the asci were found to be simple-septate. Since also the year of collection argues against *Hy. fraxineus*, the sequence taken from this sample was thought to originate from DNA of another specimen. The same suspicion arose concerning a collection made in 1987 (Zürich, Sihlwald, ZT 87–236) which has not been re-examined for croziers. Due to their age, the asserted identity of these two samples confused also other workers, e.g., Husson et al. (2011). Because of this and the observed discrepancy concerning the ascus base in the specimen from Aesch, both were resequenced for ITS and calmodulin by V. Queloz in 2012 and, indeed, turned out to represent *Hy. albidus* (Queloz et al. 2012). Their sequences were subsequently replaced in GenBank, and their identity corrected.

Different theories have been proposed concerning the origin of the causal agent of ash dieback in Europe. These include a virulent mutant, either of the native *Hy. albidus* (Kowalski and Holdenrieder 2009, p. 307) or of the introduced *Hy. fraxineus* (Queloz et al. 2011, p. 141), but even the invasion of *Hy. fraxineus* from outside Europe without associated mutation (Queloz et al. 2011, p. 141). The latter authors and Bengtsson et al. (2012) were the first who assumed an Asian origin for the pathogen. When searching for reports from outside Europe, the only reliable record on *Fraxinus* rachises that came to our notice was that from Japan by Hosoya et al. (1993), who reported the species under the name *Lambertellinia albida*, with three specimens on rachises of *Fraxinus mandshurica* var. *japonica* collected during 1990–1992 in Hokkaido and Nagano Prefectures. Re-examination of five specimens from the same prefectures collected during 2005–2011, kindly supplied by T. Hosoya and referred to *Hy. fraxineus* (as *Hy. pseudoalbidus*) by Zhao et al. (2013) based on their DNA sequence data, proved indeed to have asci arising from croziers (Table 2, TNS-F; see also Zhao et al. 2013, fig. 4). Likewise, several specimens on rachises of *F. rhynchophylla* and *F. mandshurica* from South Korea showed croziers at the ascus base, and a sequence that sits in the *Hy. fraxineus* clade (Table 2, KUS, J.G. Han personal communication).

These results, which suggest an Eastern Asian origin of the European ash dieback disease, underline the importance of detailed morphological studies in combination with molecular work. In the present case this concerns an unequivocal character state of the ascus base within the genus *Hymenoscyphus*, which is either + or – , though the elucidation of this character requires a certain skill and mounting technique. The species complex *Hy. albidus*/*Hy. fraxineus* represents one of many examples of subtle, currently neglected microscopic differences between closely related species within the ascomycetes. The present example is extraordinary because of the great economic importance of one of the two involved species.

The first report of dieback of young shoots of ash within Europe concerns regions in Northwestern Poland and dates from the year 1992 (Kowalski & Czekaj 2010). Other countries announced the disease in subsequent years (see, e.g., Timmermann et al. 2011, fig. 1; Husson et al. 2011). During the past decades *Hy. albidus* s.l. was not continuously collected and examined by the first author under the microscope, for two reasons: (1) the taxon was apparently not frequent at that time and (2) prior to the first record of croziers in 2006 it seemed to be a species that is already macroscopically well characterized by its occurrence on blackened rachises of ash. Hence, herbarium specimens were rarely kept, and a gapless elucidation of the first appearance of croziers in the ascocarps of this species aggregate cannot be accomplished.

Molecular studies performed on the two taxa by various authors strongly suggest that the causal agent of the disease is always *Hy. fraxineus*, never *Hy. albidus*. Our comprehensive microscopic examination of specimens collected since 2011 in Central Europe revealed exclusively apothecia in which the asci arise from croziers. Together with the consistent occurrence under trees that showed severe symptoms of the disease, it can be concluded that all these records represent *Hy. fraxineus*. In colline regions of Central Europe, this species starts fruiting already in May–June, distinctly earlier than *Hy. albidus* which started fruiting in July in that region. Undoubtedly, *Hy. fraxineus* has totally replaced *Hy. albidus* there, perhaps due to its high pathogenicity to the European ash and the advantage of early fruiting.

##### Earlier reports of ash dieback

The term ‘ash dieback’ is not a recent construct that emerged with the appearance of the disease caused by *Ch. fraxinea* and thus named in French more precisely ‘Chalarose du Frêne’. There were other events before with similar features as dieback of young shoots in the crown of the trees but with different causes. In the 1960s and 1970s such occurrences were reported from the US (Croxton 1966; Hibben and Silverborg 1978) and in the 1980s (Pawsey 1983) and even in the late 1990s (Wiltshire et al. 1996) from England. But they were ascribed to climatical conditions rather than to fungal infections.

##### Nomenclature

According to the new nomenclatural rules (Art. 59, ICN Melbourne, McNeill et al. 2012), the name *Hymenoscyphus pseudoalbidus* needs to be changed, because the specific epithet of *Ch. fraxinea* T. Kowalski 2006 is older than that of *Hy. pseudoalbidus* Queloz et al. 2011, while the generic name *Hymenoscyphus* Gray 1821 predates that of *Chalara* (Corda) Rabenh. 1844. Gams et al. (2012, p. 495) recommended to generally accept the epithet which is in the prioritized genus: ‘When a binomial in a prioritized genus has a younger epithet than the corresponding name in the suppressed genus, only priority of extant names in the prioritized genus should count.’ However, this proposal was later not accepted. Since molecular data on *Hy. albidus* and *Hy. pseudoalbidus* support that these species are closely related to the type species of *Hymenoscyphus* (Queloz et al. 2011), and because *Ch. fraxinea* yielded in online search engines about 5 times as many hits as *Hy. pseudoalbidus* (52.200 vs. 10.100, Google Web Search, 22.V.2014), Baral et al. (2014) proposed the new combination *Hymenoscyphus fraxineus*.

Molecular studies have shown that the genus *Chalara* in its large sense concerns anamorphs of very different ascomycetes (Cai et al. 2009): most of the investigated species were found to belong in the Helotiales, but a few are closely related to Xylariales. Species of *Chalara* s.l. connected to *Ceratocystis* Ellis & Halst. (Microascales) were transferred to the genus *Thielaviopsis* Went already by Paulin-Mahady et al. (2002).

The type species of *Chalara, Ch. fusidioides* (Corda) Rabenh., was originally described from Czechia on bark of *Pinus*, but later records were made on very different substrates such as *Fragaria* (leaves), *Podocarpus, Vitis*, and *Mycosphaerella* (perithecia), according to a redescription by Nag Raj and Kendrick (1975, p. 119, fig. 30F), who presented an illustration of the holotype. A single sequence under the name *Ch. fusidioides* exists in GenBank (18S rDNA, from *Picea*, no source given). When best-matched sequences are searched using BLAST in GenBank (http://blast.ncbi.nlm.nih.gov/Blast.cgi), only helotialean taxa are yielded, for instance members of the genera *Hyaloscypha* Boud. or *Bulgaria* Fr. but no true *Hymenoscyphus* under the first 100 hits. In the phylogenetic analysis by Réblová et al. (2011), *Ch. fusidioides* clusters in a clade with *Calycina citrina* (as *Bisporella citrina*). Whether this *Chalara* strain was correctly identified remains uncertain, as no morphological documentation was available to us. Within the relationship of *Calycina*, chalara-like anamorphs are known, e.g., in *Calycina claroflava* (Grev.) Baral et al. (Johnston 1988, as *Bisporella discedens*, anamorph *Bloxamia* Berk. & Broome), *Calycellina ulmariae* (Lasch) Korf (Baral 1989, pl. 1 fig. I, anamorph *Chalara* aff. *fusidioides*), *Calycellina carolinensis* Nag Raj & W.B. Kendr. (Nag Raj & Kendrick 1975, anamorph *Chaetochalara aspera* Piroz. & Hodges) and *Phaeoscypha cladii* (Nag Raj & W.B. Kendr.) Spooner (Nag Raj & Kendrick 1975, as *Hyaloscypha cladii*, anamorph *Chaetochalara cladii* B. Sutton & Piroz.).

Since the identity of type material of old teleomorph names is often easier to settle compared to anamorph names on the basis of a morphological reinvestigation, teleomorph genera should be given preference in regard to nomenclatural priority in those groups where teleomorphs exhibit clearer specific characteristics than anamorphs. As a consequence, we here consider *Chalara* as a form genus of anamorphs. Although the type of *Chalara* resembles *Ch. fraxinea* morphologically, the presently available molecular data do not support a close relationship with a phylogenetic group presented in an analysis in Baral et al. (2013), which can be taken as a representation of the family Helotiaceae in a restricted sense, comprising the genera *Hymenoscyphus, Phaeohelotium* Kanouse, *Cudoniella* Sacc., and *Cyathicula* De Not.

#### ***Hymenoscyphus honshuanus*** Baral, **nom. nov.** – **Figure 12** – MycoBank MB 809090

**Figure 12.**
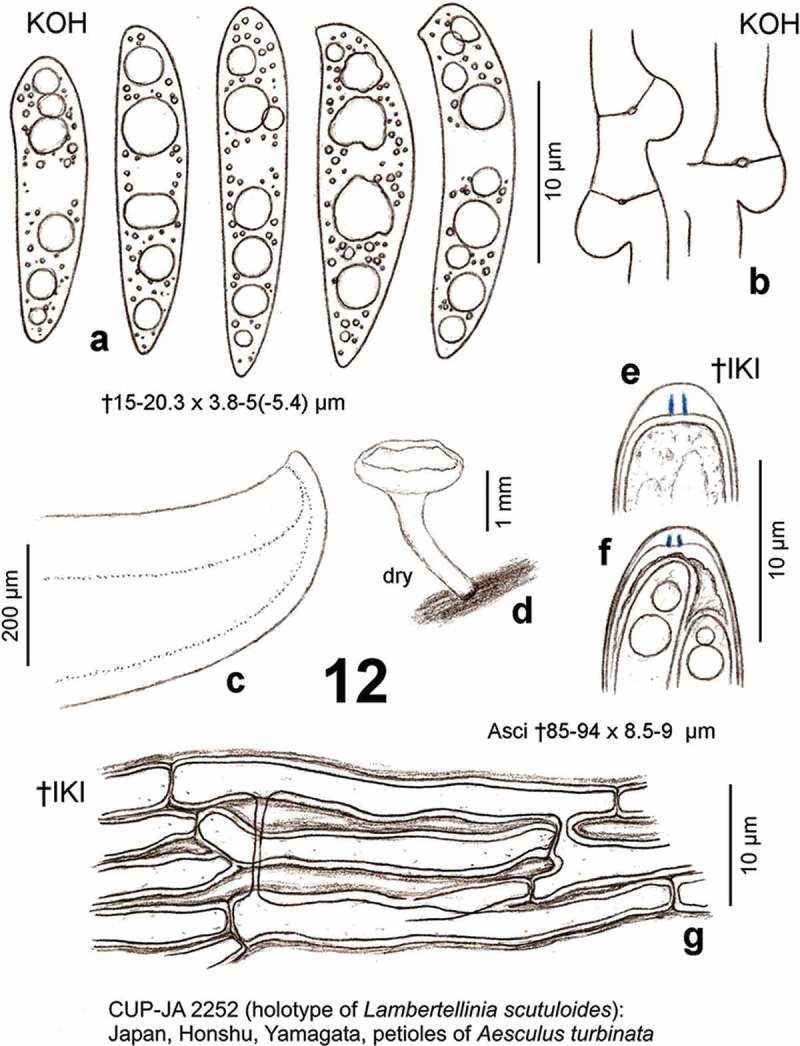
*Hymenoscyphus honshuanus* (holotype of *Lambertellinia scutuloides*): a. ascospores (partly from inside asci, containing LBs); b. ascogenous hyphae with crozier formation; c. median section of receptacle; d. apothecium; e–f. ascus apices with euamyloid apical ring (*Hymenoscyphus*-type); g. ectal excipulum at flanks (median section), with gelatinized walls. – All in dead state.

Basionym: *Lambertellinia scutuloides* Korf & Lizoň, Mycotaxon 50, p. 168 (1994) [non *Hymenoscyphus scutuloides* Hengstm., Persoonia 16, p. 199 (1996)]
≡ *Lambertellinia scutuloides* Korf & Lizoň, Inoculum, Newsletter of the Mycological Society of America 44(2), p. 43 (1993); nom. inval., Art. 39.1 (Melbourne)

##### Etymology

*Honshuanus*: named after the geographical origin; *scutuloides*: according to the scutuloid ascospores.

##### Holotype

Japan, Honshu, Yamagata, Oguni-machi, Tsugawa, petioles of *Aesculus turbinata*, 28.VIII.1961, D. Shimizu (CUP-JA 2252; Figure 12).

##### Apothecia

Rehydrated ~2–3 mm diam., disc light orange- to golden-yellow, concave to flat, exterior pale yellow; stipe 1–4 × 0.2–1 mm, concolorous with receptacle, sometimes brownish at the base, usually with a black ring of stromatal tissue at the base; dry entirely ochraceous. **Asci** †85–94 × 8.5–9 µm, 8-spored, spores †biseriate or irregularly uniseriate; **apex** (†) conical, dome †1.7 → 1 µm thick, apical ring occupying the lower 2/3 of dome, strongly blue in IKI (bb), *Hymenoscyphus*-type; **base** arising from croziers {two apothecia tested}. **Ascospores** †15–20.3 × 3.8–5(–5.4) µm, mostly distinctly scutuloid, cylindric–fusoid, apex obtuse, with a slight to strong lateral protrusion, base medium attenuated, often subacute, straight to slightly inequilateral or curved, containing ~2–3(–5) medium- to large-sized LBs (1.3–)1.7–3 µm diam. and many small ones in each half, lipid content 4.5–5; sheath not observed, setulae absent; spore wall (probably overmature) partly turning pale to light ochre-brown with age, remaining smooth, occasionally 1-septate. **Paraphyses** apically uninflated, †2–2.8 µm wide. **Ectal excipulum** hyaline, at lower flanks of textura porrecta oriented at a low angle to the surface, cells broadly brick-shaped, †~60–75 × 5–10 µm, with seemingly strongly gelatinized walls (but in KOH only slightly so), externally covered by narrow, straw- to golden-yellow hyphae. **Anamorph**: unknown.

##### Habitat

On blackened, previous year’s petioles of *Aesculus turbinata* {1}. **Desiccation tolerance**: unknown. **Altitude**: ‘500–800 m’.

##### Characterization

*Hy. honshuanus* is not easily separable from *Hy. fraxineus* by its micromorphology, but the deviating substrate and the apothecia with a yellowish disc and longer stipes appear to be diagnostic. As already mentioned, the only difference between *Hy. honshuanus* and *Hy. fraxineus* worth mentioning seems to be the longer apothecial stipe and a more yellowish disc in the former species, apart from the deviating substrate. The excipular cells are a bit longer and narrower than in *Hy. fraxineus*. The lower width is to some extent due to the shrinking effect in the dead state, however. Moreover, the width of the ectal excipular cells in *Hy. fraxineus* varies rather strongly. Regrettably, a section of the blackened petioles and the stipe base of *Hy. honshuanus* was not studied, neither by Korf and Lizoň nor in the present study; therefore, the occurrence of a demarcation line beneath the sclerenchyma as seen in *Hy. aesculi* remains unclear, and also the presence of crystals in the stipe base, being characteristic of *Hy. fraxineus* and *Hy. albidus*, remains to be examined.

##### Nomenclature

*Hy. honshuanus* was originally described by Korf and Lizoň (1994) under the name *Lambertellinia scutuloides* from Japan on blackened petioles of *Aesculus turbinata* and is known with certainty only from the type collection. A transfer of *Lambertellinia scutuloides* to *Hymenoscyphus* is blocked by the different caulicolous taxon *Hy. scutuloides* Hengstm.; therefore, a new name is here proposed. The species was presented already by Korf and Lizoň (1993) on a poster at the Annual Meeting of the Mycological Society of America, with reference to an earlier volume of Mycotaxon (47, 1993), but it did not actually appear there.

##### Type study and circumscription

According to the present redescription of the holotype, which was briefly mentioned also in Galán and Baral (1997), the taxon deviates from *H. aesculi* and *H. albidus* in the presence of croziers and some other features (see also under those species). While the ascospores of *H. aesculi* lose their characteristic central swelling in the dead state, it is unknown whether living spores of *H. honshuanus* might also show such swelling.

Korf and Lizoň (1994) thought that *Lambertellinia scutuloides* resembles the genus *Lambertella* Höhn. in its black substratal stroma and the ascospores getting finally light brown, but the authors placed it in a new genus because of its long-celled excipular structure, the assumed connection to an *Idriella* anamorph, and probably also because of the deviating scutuloid spore shape, to which the original species epithet refers. Regrettably, no microscopic features were illustrated in the protologue, except for a photograph showing a section of the excipulum.

Korf and Lizoň believed that collections on leaves of *Acer rubrum* from North America and *Berberis vulgaris* from Germany are conspecific, though they examined only that on *Berberis*. The collection on *Acer rubrum* was treated by Kimbrough and Atkinson (1972) under the name *Hymenoscyphus caudatus*. Unlike *Hy. fraxineus*, a strain from this collection formed an *Idriella* anamorph in pure culture, with slightly falcate conidia measuring ?15 × 2 µm (evaluated from photos), formed in sporodochia in a sympoduloconidial type of development. The original material was not available to Korf and Lizoň, who were sure about its conspecificity with *Lambertellinia scutuloides* because of the reported brown colouration of the 1–2-septate ascospores when ‘mature’. However, Kimbrough and Atkinson did not observe stromatic tissues around the inhabited midribs and larger veins, although they saw the stipe base to arise from darkened host tissue. Regrettably, Kimbrough and Atkinson did not report the presence or absence of croziers, though they stated that the features of their fungus were essentially the same as in *Hy. caudatus* as described by White (1943) and others.

Korf and Lizoň (1994) characterized the ectal excipulum of *Lambertellinia scutuloides* as a hyaline textura porrecta consisting of gelatinized hyphae 3–6.5 µm wide, externally covered by a thin layer of straw- or golden-yellow pigmented hyphae 0.5–1.5 µm wide, and internally delimited by a light yellow layer. The authors considered this construction of the ectal excipulum as different from typical members of *Hymenoscyphus*. However, the present re-examination of the excipular texture (Figure 12(g)) revealed no substantial differences from, e.g., *Hy. albidus, Hy. fraxineus*, and *Hy. aesculi*.

The description given above is based on the present re-examination of the holotype, but it includes also some data of the protologue, mainly in regard to macroscopical features. The petioles from which the apothecia of *Hy. honshuanus* arise show a dark blackish-grey colour. Korf and Lizoň referred to it as a ‘black substratal stroma’. Similar as in the other species treated in the present paper, a black ring was often seen at the outermost base of the stipe (Figure 12(d)). Contrary to Korf and Lizoň, who stated the asci to be simple-septate, the present examination of two apothecia showed that they arise from croziers (Figure 12(b)). Since the authors did not illustrate the feature, their report of absent croziers remains mysterious. Apart from the abundant hyaline ascospores, only a single spore with a pale ochre-brown wall was seen in the present examination. It must be stated, however, that the asci, spores and paraphyses were distinctly wider than indicated by Korf and Lizoň, who measured them as follows: asci (82–)84.5–100(–116) × (5.6–)6.6–7.5(–8.3) µm, spores (13.8–)15.4–16.9(–18.5) × (2.3–)3.1–3.5(–3.8) µm, paraphyses 1.5 µm wide. The protologue appears to be based exclusively on the Japanese type specimen. Data of the specimen from Germany (on *Berberis*) were not incorporated, which is obvious from the mentioned larger spore size in the *Berberis* specimen (Korf & Lizoň 1994, p. 171).

We assume that *Hy. honshuanus* was incorrectly described by Korf and Lizoň (1994) concerning the ascus base, though it cannot be excluded that the holotype collection represents a mixtum of two species with deviating ascus base characters as well as ascus and spore measurements. In any case, the present data indicate that the here described specimen is not conspecific with either *Hy. aesculi* or *Hy. caudatus. Hy. caudatus* was redescribed by White (1943, p. 151, fig. 8), who observed the absence of croziers in most (though not all) of the many examined specimens, including the holotype. Regrettably, White did not specify the aberrant material in which he saw croziers.

Because of the striking morphological similarity with *Hy. fraxineus*, it would be interesting to determine the ITS sequence of *Hy. honshuanus*. However, this approach requires careful examination of single apothecia for the presence of croziers, in order to avoid confusion in the case of a mixtum. Actually, a further Japanese record (on petioles of *Aesculus* sp. kindly sent by T. Hosoya, TNS-F-12758) differs from *H. honshuanus* in the ascus base, indicating that two different species exist in Japan: the asci arise here from simple septa (Figure 15), and the species is, therefore, included in *Hy. aesculi*.

With the present data, Korf and Lizoň’s conclusion that *Lambertellinia scutuloides* has an *Idriella* anamorph needs to be reinvestigated. Experiments with cultures of the common and plurivorous *Hy. caudatus*, but also *Hy. vacini*, a taxon specific to leaves of *Acer*, kept under varying conditions as proposed by Kimbrough and Atkinson (1972), could clarify whether or not any of these species are connected to an *Idriella* anamorph.

The German specimen on *Berberis* (Sydow, Mycotheca Marchica no. 1576, 1887) remains of unclear identity. The apothecia are said to arise from stromatized veins and petioles, but the presence or absence of croziers was not stated by Korf and Lizoň. When dry, the apothecia were ‘dark brown (almost blackish)’. The spores measured 16.2–19.8 × 3.6–4.8 µm and were ‘cylindric-clavate, scutuloid, deep brown to hyaline’. The exsiccatum was issued under the name *Helotium berberidis* Syd., but without a diagnosis on the label (Korf & Lizoň 1994).

*Hy. honshuanus* is known with certainty only from the type collection on *Aesculus turbinata.* Reports under the names *He. robergei* or *Hymenoscyphus albidus* on fallen petioles of *Aesculus indica* in Northern India and Nepal might well concern *Hy. honshuanus* (or *Hy. aesculi*), but the ascus base was not studied for the presence of croziers (see discussion under *Hy. albidus*).

The type locality of *Hy. honshuanus* is not exactly known, and the collector, Daisuke Shimizu, who was a zoologist specialized in vegetable wasps, passed away in 1998. The small town Oguni(-machi) belongs in the Nishiokitama district and lies 55 km WSW of Yamagata. The village Tsugawa could not be located (it was included in Oguni in 1960), but its altitude was found to be 294 m (T. Hosoya personal communication). Oguni has an altitude of about 140 m; therefore, the type locality must be in one of the adjacent mountain ranges.

##### Specimens included

**Japan**: **Honshu, Yamagata**, Oguni-machi, Tsugawa, on petioles of *Aesculus turbinata*, 500–800 m, 28.VIII.1961, D. Shimizu (CUP-JA 2252, **holotype** of *Lambertellinia scutuloides*, vid. 14.IV.1997).

#### ***Hymenoscyphus aesculi*** (Velen.) Baral & E. Rubio, **comb. nov.** – **Figures 13**–**19** – MycoBank MB 809091

**Figures 13–15.**
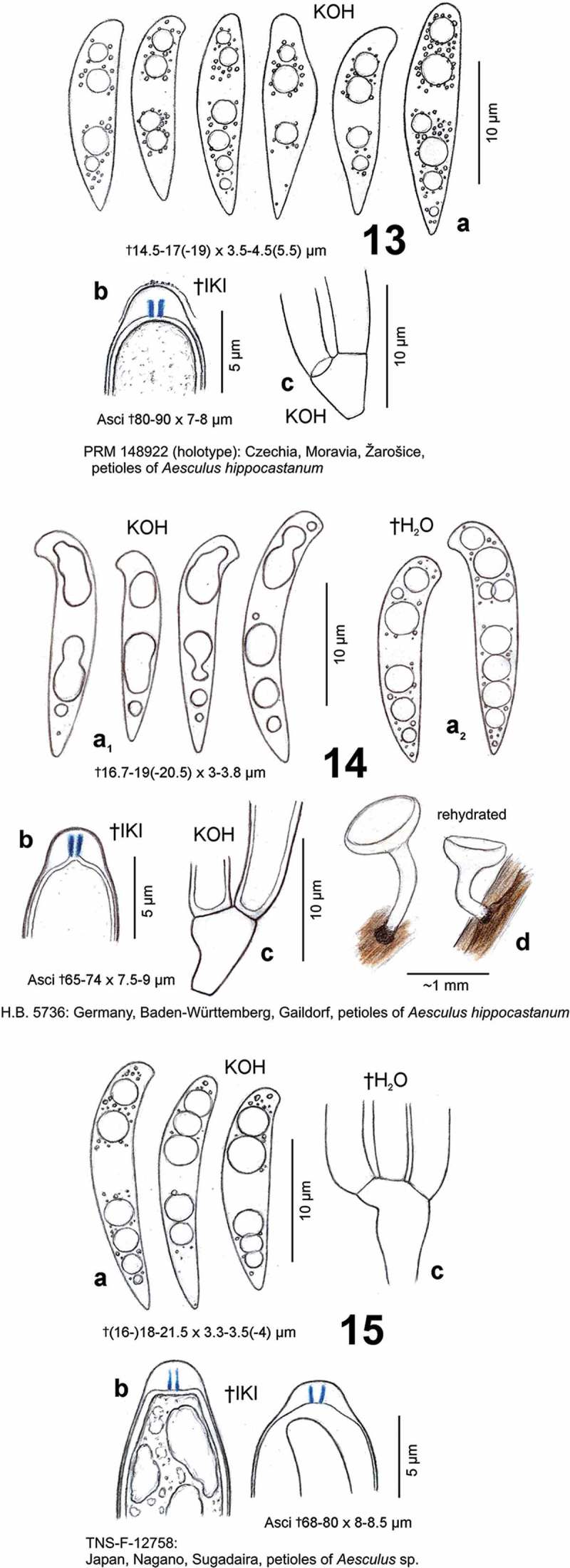
*Hymenoscyphus aesculi*. a. ascospores (partly from inside asci, containing LBs); b. ascus apices with euamyloid apical ring (*Hymenoscyphus*-type); c. ascus bases arising from simple septa; d. apothecia. – All in dead state.

**Figure 16.**
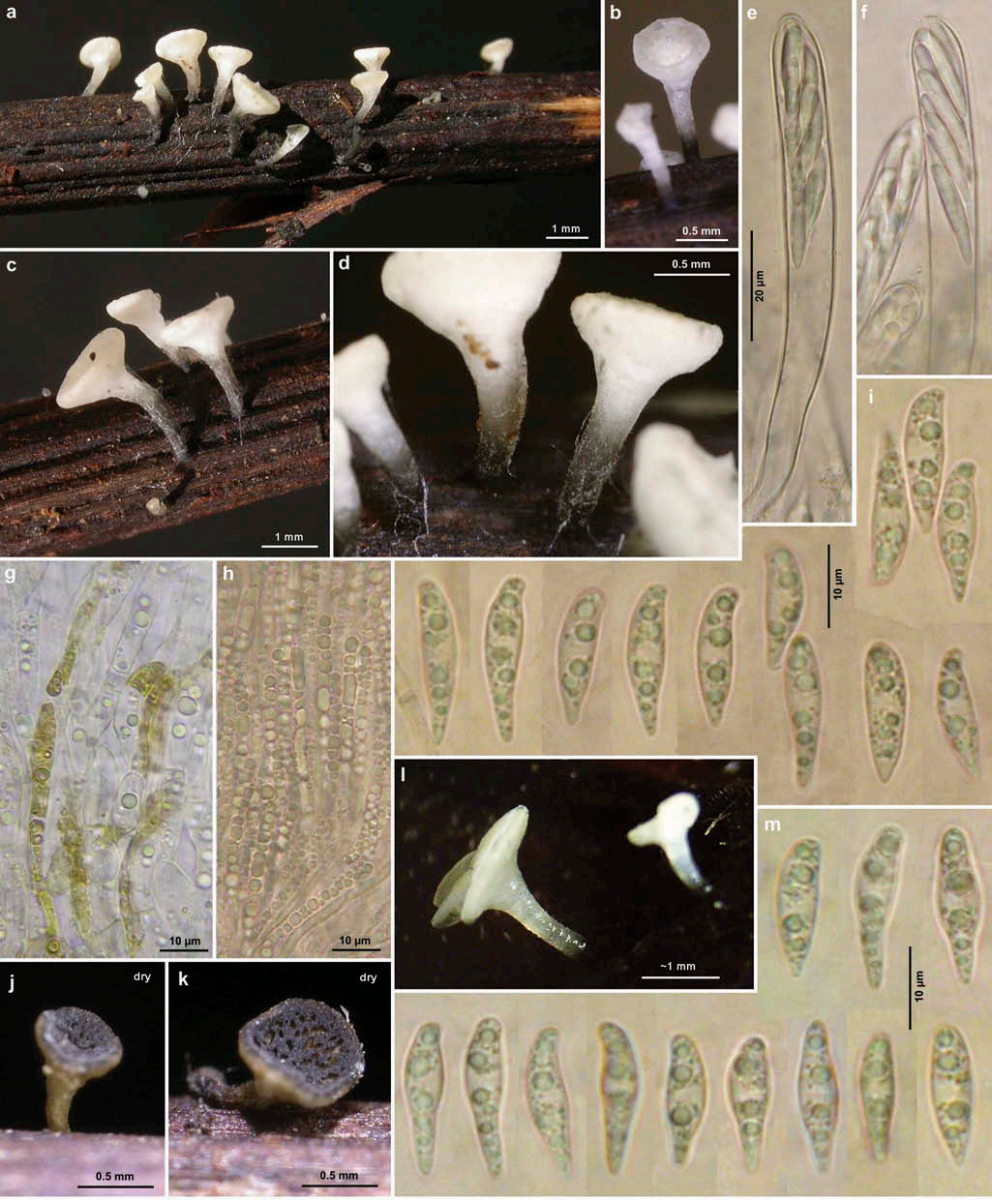
*Hymenoscyphus aesculi*: a–d, l. fresh apothecia; j–k. dry apothecia (phot. over 3 years after collection); g. cortical ectal excipulum of receptacle in surface view (VBs in living cells, distorted brass-yellow cytoplasm of scattered external hyphae); h. paraphyses in squash mount (containing VBs); e–f. turgescent mature asci; i, m. ascospores (freshly ejected, containing LBs). – All in living state (except for j–k, brass-yellow cells in g). – a–f, h–i. H.B. 8914 (DE-NI, Hamburg); g. H.B. 9843 (DE-BW, Tübingen); l–m. H.B. 9417 (DE-SN, Chemnitz). – Phot. l: B. Mühler.

**Figure 17.**
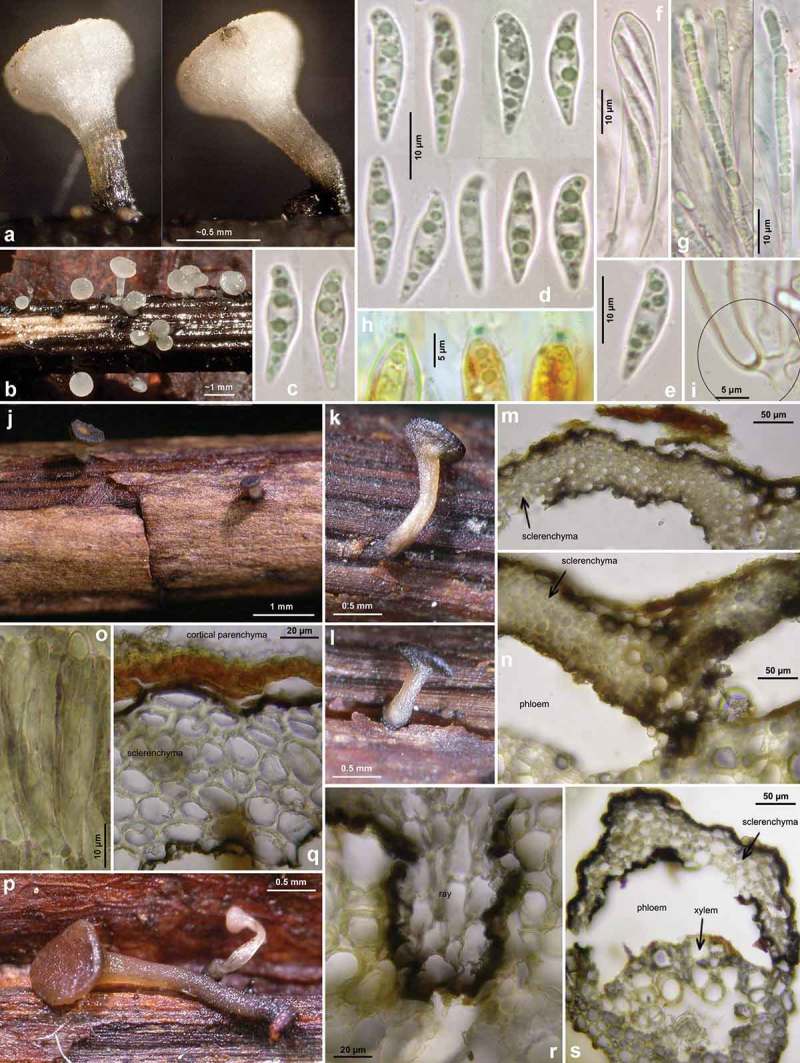
*Hymenoscyphus aesculi*: a–b. fresh apothecia; j–l, p. rehydrated apothecia (colour was originally white except for base of stipe); f–g, o. asci and paraphyses(o: with olive secondary pigment especially in paraphyses); h. ascus apices with euamyloid apical ring; i: simple-septate ascus base; c–e. ascospores; m–n, q–s. cross section through cortical region of petioles, with black demarcation line surrounding the sclerenchyma on outer and inner face (cortical parenchyma above and phloem beneath being entirely decomposed). – Living state: c–g; dead state: h (in IKI), o (in H_2_O). – a–i. E.R.D. 5685 (ES-Asturias, Somiedo); j–o. H.B. 5736 (DE-BW, Gaildorf); p–s. TNS-F-12758 (JP-Honshu, Nagano, Sugadaira). – Phot. a–i: E. Rubio.

**Figure 18.**
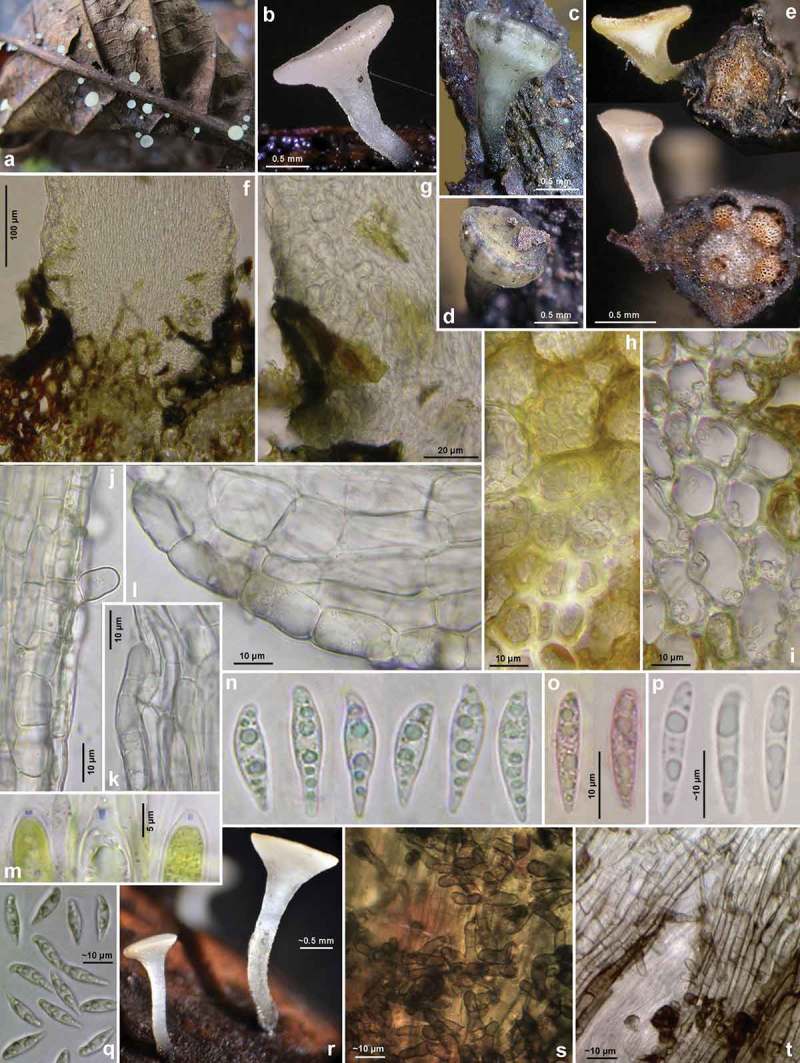
*Hymenoscyphus aesculi*: a–b, e, r. fresh apothecia (e: in median section, petioles in cross section); c–d. senescent apothecia (with dark bluish-olive patches on receptacle); f–g. stipe base in median section, showing erumpent growth and absence of crystals; h. cortical parenchyma and sclerenchyma below insertion of apothecial stipe, densely filled with intracellular hyphae; i. sclerenchyma forming a radial ray, loosely filled with hyphae; j–l. ectal excipulum at stipe and (l) at lower flanks in median section, showing VBs in cortical cells; m. ascus apices with euamyloid apical ring; n–q. ascospores (n–o, q: freshly ejected); s–t. surface view on stipe base, showing brown cortical cells and hairs. – Living state, except for m (in KOH+IKI), o (in KOH+CRB), p (in H_2_O?). – a. 31.VII.2011 (DE-HS, Darmstadt), b–p. H.B. 9701 (Darmstadt), q–t. C.Y. F/2194 (UK-Yorkshire, Halifax). – Phot. a, p: H. Lotz, q–t: C. Yeates.

**Figure 19.**
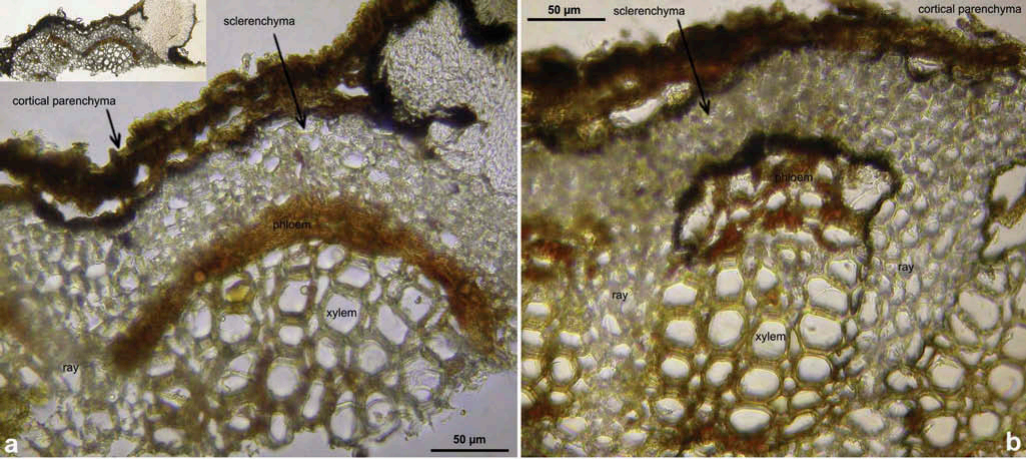
*Hymenoscyphus aesculi*: a–b. petiole in median section, showing black stroma and erumpent apothecial growth; pseudosclerotial plate occurring between cortical parenchyma and sclerenchyma but also between sclerenchyma and vascular bundle (phloem/xylem). – a–b. H.B. 9701 (DE-HS, Darmstadt).

Basionym: *Helotium aesculi* Velen., Opera Bot. Čech. 4, p. 120 (1947)
≡ *Lanzia aesculi* (Velen.) Svrček, Acta Mus. Nat. Pragae 40 B, p. 131 (1985, ‘1984’)
(?) = *Hymenoscyphus albidus* var. *aesculi* W. Phillips, Man. Brit. Discom. p. 138 (1887)
≡ *Phialea albida* var. *aesculi* (W. Phillips) Saccardo, Syll. Fung. 8, p. 254 (1889)
≡ *Helotium albidum* var. *aesculi* (W. Phillips) Massee, Brit. Fung.-Fl. 4, p. 260 (1895)

##### Etymology

Named after the inhabited substrate, leaves of *Aesculus hippocastanum*.

##### Holotype

Czechia, South Moravia, SE of Brno, Žarošice, petioles of *Aesculus hippocastanum*, IX.1942, V. Vacek (PRM 148922; Figure 13).

##### Apothecia

Moist (0.3–)0.6–1.5(–2) mm diam.; disc whitish(-greyish) or pale cream, slightly concave to flat, margin distinct, not protruding, smooth, exterior concolorous; stipe (0.25–)0.5–1.7(–2) × (0.08–)0.15–0.35(–0.42) mm, white, glassy-translucent or non-translucent, slightly pubescent, lower half gradually darker, near base black, erumpent from sclerenchyma-phloem layer; hymenium dry light to dark greyish- to blackish-brown, exterior pale olivaceous-grey. **Asci** *86–100(–104) × 9–10(–11.5) µm {4}, †(62–)65–75(–80) {4} or (67–)75–100(–116) {5} × (7–)8–9 {7} or (8.5–)9–9.5(–10) {2} µm, 8-spored, spores *obliquely biseriate, pars sporifera *44–54 µm long, *7–10 µm protruding beyond paraphyses; **apex** (*/†) conical, dome †1.5–2 → 1–1.3 µm thick, apical ring occupying lower 1/2–9/10 of dome, medium to strongly blue (bb) in IKI, apically gradually or abruptly inamyloid, *Hymenoscyphus*-type {11}; **base** arising from simple septa {15}, never with a subbasal protuberance. **Ascospores** *(14–)15–20(–22)((–24)) × (3.7–)4.2–5(–5.2) µm {12}, †(14.5–)16–19(–21.5) × (3–)3.3–4(–5.5) µm {4}, strongly scutuloid, (*) fusiform with inflated middle part {14}, (†) cylindric-fusoid, median inflation much less distinct, apex rounded to obtuse, with a slight to strong lateral protrusion, base attenuated, ± acute, straight, sometimes inequilateral or slightly, rarely medium curved; containing (1–)2–3(–4) **LBs** (1–)1.5–2.5(–2.8) µm diam. and several small ones in each half {15}, lipid content 4–5; with very delicate sheath that detaches from the wall when ejected, setulae absent; overmature eguttulate, aseptate, (†) pale to light brown (cytoplasm?). **Paraphyses** apically uninflated, terminal cell *50–75 × 3.5–4.5(–5) µm {1}, †25–55 × (2–)2.5–3.5(–4) µm {T}, lower cells *8–20 × 2–4(–6) µm {1}. **Medullary excipulum** of ± dense, vertically orientated textura porrecta or more loose t. intricata in central region (towards hymenium t. prismatica), individual cells *(16–)25–55(–100) × (6–)8–12(–15) µm {2}, medium sharply delimited from ectal excipulum by a 30–40 µm thick layer of textura porrecta, individual cells *60–100 × 5–10 µm {1}. **Ectal excipulum** hyaline, of (*) thin-walled († very slightly gelatinized) textura prismatica, at lower flanks 30–70 µm thick, cells *15–30(–50) × (8–)12–22 µm {3}, †11–23 × 12–20 µm {2}, brick-shaped, at margin 15–20 µm thick, cells *9–14 × 8–12.5 µm, externally covered by a scattered network of hyphae, individual cells *15–25 × 4.5–11 µm {2}; cortex of stipe near base with slightly gelatinized brick-shaped cells *7–23 x 5–9 µm {1}, †9–15 × 8–10 µm {T}, at very base medium gelatinized, sparsely to densely covered by short, hyaline to bright grey-brown hairs 10–20 × 3–7.5 µm {2}. **VBs** in paraphyses medium to strongly refractive {9}, hyaline, multiguttulate but soon angular to very elongate, occupying upper (25–)35–60(–83) µm {2}, staining yellowish in IKI, turning bright brass-yellow to olivaceous-brown in dead cells or in the herbarium; rarely with a few pale yellow-orange LBs up to 1 µm diam.; VBs in cortical hyphae of stipe and receptacle medium refractive, large and elongate, or strongly refractive, globose. **CRB**: spore wall unstained or faintly lilac, gel in ectal and medullary excipulum deep lilac, VBs bright turquoise. **Crystals** absent in entire apothecial tissue. **Exudate** on exterior of receptacle and stipe absent. **Anamorph**: unknown.

##### Habitat

On fallen previous year’s dark brown to blackish petioles {13}, also on darkened primary and secondary veins {8} (upper and especially lower face) of *Aesculus* sp. {1}, *A. hippocastanum* {21}. **Assoc.**: *Allophylaria nervicola* {1}, *Calycellina lachnobrachya* {1}, *Calycellina* ?*leucella* {1}, *Calycina discreta* {1}, *Hymenoscyphus caudatus* {1}, *Mollisina acerina* {1}. **Desiccation tolerance**: not examined. **Geology**: löss, limestone, etc. **Phenology**: (VII–)VIII–IX(–X). **Altitude**: 3–700 m (in Europe), 1325 m (in Japan).

##### Specimens included

All on *Aesculus hippocastanum*, except for Japan: *Aesculus* sp.

**England**: **Yorkshire**, 8.5 km SSE of Halifax, 1.8 km NW of Huddersfield, Edgerton area, 148 m, veins, 24.VIII.2012, C. Yeates (C.Y. F/2194, d.v.). – **Czechia**: **South Moravia**, 30 km SE of Brno, Žarošice, ~220 m, petioles, IX.1942, V. Vacek (PRM 148922, **holotype**, vid. 19.XII.2011). – **Belgium**: **West Vlaanderen**, 7.5 km ENE of Ostende, 2.5 km NE of Bredene, D’Heye, 3 m, petioles, 19.VIII.1994, B. Declercq (B.D. 94/105, n.v.). – 5 km ESE of Brugge, 1.8 km WSW of Sijsele, Rijckevelde, 12 m, petioles, 2.IX.2000, B. Declercq (B.D. 00/073, d.v.). – **Oost Vlaanderen**, 16 km NE of Gent, 3.8 km SSE of Wachtebeke, Puyenbroeck, 3 m, petioles, 14.VIII.1993, B. Declercq (B.D. 93/137, d.v.). – **Germany**: **Niedersachsen**, ~8 km E of Walsrode, ?1 km NE of Fallingbostel, Böhmetal, Geesthang, ~40 m, petioles and veins, 19.VIII.1990, B. Grauwinkel (d.v.). – 12 km S of Hamburg, Harburg, city park, 25 m, petioles, 27.VIII.2008, M. Vega (H.B. 8914). – **Sachsen**, 3.5 km NE of Chemnitz, Zeisigwald, 380 m, veins, 15.VIII.2010, B. Mühler (H.B. 9417ø). – **Hessen**, 8.7 km NE of Darmstadt, 1 km W of Messel, Mörsbacher Grund, 170 m, petioles and veins, 31.VII.2011, H. Lotz-Winter (d.v.). – ibid., petioles and veins, 10.VII.2012 (H.B. 9701). – **Baden-Württemberg**, 7.5 km ESE of Gaildorf, 2.5 km NE of Sulzbach-Laufen, Kohlenstraße, Brandwald, 490 m, petioles, 26.VIII.1996, W. Hena (H.B. 5736, vid. 25.III.1997). – 6 km W of Stuttgart, 2.3 km SSE of Solitude, Bärensträßle, 455 m, leaf, 5.IX.1975, H.O. Baral (H.B. 474ø). – 6 km WSW of Stuttgart, 2.5 km S of Solitude, Bärenkopf, 440 m, petioles and veins, 18.VII.1988, H.O. Baral (H.B. 3467). – 6.3 km NE of Tübingen, 1.8 km NNE of Pfrondorf, Rotes Tor, 475 m, veins, 22.X.1991, H.O. Baral (ø). – 2 km NW of Pfrondorf, Ziegelhäule, 465 m, petiole, 13.X.2013, H.O. Baral (ø). – 8 km NE of Radolfzell, 1 km SE of Bodman, S of castle, 430 m, leaf, 17.VIII.1976, H.O. Baral (ø). – **Bayern**, 13 km SE of Coburg, 1 km SW of Weidhausen, Rödertal, 310 m, petioles, 8. & 24.VIII.1987, H. Engel (PRM, H.E. 8433). – 10.5 km SW of Starnberg, 2 km NW of Tutzing, Deixlfurter See, 700 m, petioles, 13.VIII.2006, B. Fellmann (ø). – **Switzerland**: **Schaffhausen**, 2.5 km NE of Schaffhausen, 1.8 km W of Gennersbrunn, Rheinhardt (Solenberg), 480 m, petioles, ~20.VIII.1988, P. Blank (ø). – **Spain**: **Asturias,** 22.5 km NNE of Villablino, 5 km N of Somiedo, NW of Castro, La Malva, 560 m, petioles, 13.X.2012, E. Rubio (E.R.D. 5685, d.v.). – **Japan**: **Honshu, Nagano**, 19 km SE of Nagano, 1 km SE of Sugadaira, Arboretum, 1325 m, veins, 11.IX.2006, T. Hosoya (TNS-F-12758).

##### Characterization

*Hymenoscyphus aesculi* resembles *Hy. albidus* in many respects, including simple-septate ascus bases. Like *Hy. albidus*, it occurs wide-spread within Europe, where it appears to be specific to leaves of *Aesculus hippocastanum*. Yet, a single Japanese record indicates a wider distribution area for *Hy. aesculi*. The species differs from *Hy. albidus* in several characteristics: (1) the living ascospores consistently show a central swelling (bulge); therefore, they look fusiform rather than cylindrical to fusoid as in *Hy. albidus*; (2) the internal tissue of the stipe base is entirely devoid of crystals; (3) *Hy. albidus* generally shows a thin yellowish layer of exudate on the entire exterior, especially on the stipe, which is absent in *Hy. aesculi*; (4) senescent apothecia turn dark olivaceous-grey to blackish-brown whilst those of *Hy. albidus* turn cream to orange though sometimes also dark brown. These differences hold true also for a comparison with *Hy. fraxineus*, the latter differing from the other two in having croziers.

##### Type studies and circumscription

In the protologue of *Helotium aesculi*, Velenovský (1947) described the apothecia as white, 2–4 mm diam., later with a black stipe, the spores as 12–17 × 6–7 µm, with an indistinctly acute base and a series of guttules, and the asci as 130–150 × 6–8 µm. In comparison with the here presented description, these measurements fit only concerning spore length and ascus width, whereas apothecial size, spore width and ascus length are extraordinary large and perhaps erroneous, even when taking living elements into consideration.

Svrček (1989) did not describe the type material of *He. aesculi*, but he stated that it fully concurs with some fresh specimens studied by him. This is astonishing, because he measured the asci in one of his collections with 65–85 × 7–10 µm without discussing the discrepancy to the protologue. He also noted (p. 74) that ‘the original Velenovský diagnosis is not accurate if compared with V. Vacek’s [the finder of the holotype] handwritten notes’.

No such handwritten notes were seen when the holotype was re-examined in the present study. It revealed an ascus size (see Figure 13) compatible with Svrček’s above-mentioned measurement which likewise undoubtedly refers to dead asci. The contents of many paraphyses were bright olivaceous-brown, and in cross section of a petiole a distinct black stroma could be seen which surrounds the sclerenchyma on its outer as well as inner face.

Svrček (1989, pl. 2 fig. 4) illustrated a personal collection from near Praha, which he considered conspecific with the type of *Helotium aesculi*. Concerning his reasons to transfer the species to *Lanzia*, Svrček (1985) referred to a paper in press which, however, does not treat this species (see also under *Hy. vacini*). Probably he proposed the combination because of the blackish stroma, but in 1989 he mentioned merely the dark stipe base, not the substratal stroma. The drawn spores look as if they were in the living state, according to the regular lipid content. Svrček (1989, p. 20) described them as ‘fusiform, clavate, often strongly tapering below and curved, filled with many small and larger guttules’, 15–24.5 × (3.5–)4–5 µm, i.e., rather variable in length. The spores lack the central swelling, however, and the LBs are drawn distinctly smaller than in any of the here studied collections. Svrček examined also a further specimen from Southern Bohemia and one that H. Engel sent to him from Weidhausen near Coburg. The latter collection was also examined in the present study and found to have predominantly strongly fusiform spores with a central bulge and four rather large LBs, unlike the sketch by Engel and Hanff (1989, p. 40) that shows no central bulge and only rather small LBs.

In all collections of *Hy. aesculi* studied by the first author in the fresh state, the characteristic central swelling of the living spores was noted in a major part of the freshly ejected spores (see Figures 16(i), 16(m); 18(n)). A central swelling is also obvious in some or all spores of fresh collections drawn by B. Grauwinkel (personal communication) from Fallingbostel (Niedersachsen), B. Fellmann (personal communication) from Starnberg (Oberbayern), B. Declercq (personal communication) from Wachtebeke (Eastern Flanders), and Beyer (1998) from Bayreuth (Oberfranken). This swelling is further evident in almost every spore of a British record photographed by C. Yeates (personal communication, Figure 18(p)), and in one from Asturias by E. Rubio (personal communication, Figure 17(c)–(d)).

However, the peculiar spore shape of *Hy. aesculi* is state-dependent and well recognizable only in living spores. The loss of the spore bulge under treatment with KOH is illustrated on Figure 18(n)–(o). Apart from the holotype, two specimens of *Hy. aesculi* were studied in the dead state only (Figure 14–15), and these were first thought to deviate from typical *Hy. aesculi* in ascospores devoid of a central swelling. From the observed influence of spore turgor on spore shape; however, we assume that they also possessed a central swelling in the living state. In the holotype of *Hy. aesculi* (Figure 13), a few of the fusoid spores still showed the median swelling characteristic of the living spores. In conclusion, one of the most diagnostic markers to separate *Hy. aesculi* from *Hy. albidus* is partially or entirely obscured in herbarium material.

The medullary excipulum in the central part of the receptacle of *Hy. aesculi* was found to be composed of a rather dense, upwards oriented textura prismatica-porrecta, unlike *Hy. albidus* and *Hy. fraxineus* for which we have always observed a loose t. intricata. However, this feature was only tested in one collection (H.B. 9701) and needs further study.

All presently known European records of *Hy. aesculi* grew on leaves of *Aesculus hippocastanum*. The species fruits between July and October, predominantly in August. A distribution map under the name *Lanzia aesculi* for W-Germany is presented in Krieglsteiner (Krieglsteiner 1993, pl. 744), with 13 grid squares distributed in Niedersachsen, Bayern, and Baden-Württemberg (including Northern Switzerland). One of the two examined Japanese specimens on blackened *Aesculus* petioles (as *Lanzia* sp.) is here referred to *Hy. aesculi* (Figure 15, 17(p)–(s)) because of the absence of croziers and the lack of crystals in the stipe base; the other is the type of *Hy. honshuanus* (≡*Lambertellinia scutuloides*) and deviates from *Hy. aesculi* in having croziers.

##### Nomenclature, misidentification as *Hymenoscyphus albidus*

Authors appear to have previously merged *Hy. aesculi* under *Hy. albidus*. This is undoubtedly true for reports on petioles of *Aesculus* from Great Britain under that name (FRDBI). However, we here provide arguments that two different species of *Hymenoscyphus* occur on that substrate. They were formerly treated in the Sclerotiniaceae under the names *Lan. aesculi* (Velen.) Svrček (1985) and *Lambertellinia scutuloides* Korf and Lizoň (1994), but are transferred to *Hymenoscyphus* in the present paper based on their morphological similarity to *Hy. albidus* and their overall characteristics suggesting this genus. The latter is here given the new name *Hy. honshuanus*, and differs from *Hy. aesculi* in the presence of croziers, also in wider ascospores, yellowish apothecia that do not turn dark with age, longer stipes, and narrower ectal excipular cells.

Phillips (1887, p. 138) distinguished between typical *Hymenoscyphus albidus* (on petioles of *Fraxinus*) and *Hy. albidus* var. *aesculi* (on petioles of *Aesculus*), based on a collection from Shobdon Court, Herefordshire, on the basis of larger, more frequently clavate spores (20–23 × 4–5 µm). Phillips’ brief diagnosis does not mention a darkened substrate, but his remark ‘the stem is often brown at the base’ in *Hy. albidus* suggests that this character was also present in his specimen on *Aesculus*. Massee (1895, p. 260) and Saccardo (1889, p. 254) merely copied Phillips’ protologue. The type material has apparently never been re-examined. Judging from the clavate spores this might well be a synonym of Velenovský’s *He. aesculi*. A spore length around 23 µm was only very rarely seen in the present study, but falls in the wide range given by Svrček. Records on petioles of *Aesculus* under the name *Hymenoscyphus albidus* in the database of the British Mycological Society very probably also belong to *Hy. aesculi*. Also collections on leaves of *Aesculus indica* in Northern India and Nepal reported by Thind and Singh (1969) and others might belong to *Hy. aesculi* (or *Hy. honshuanus*), but the ascus base was not studied for the presence of croziers (see discussion under *Hy. albidus*).

*He. scutula* var. *aesculicarpa* Syd. was described from capsules of *Aesculus* and probably represents a member of the *Hymenoscyphus fructigenus*-complex. Dennis (1964, p. 64) examined the type material and also further British specimens on *Aesculus* capsules and petioles. He concluded that all of them belong in the scope of the collective species *He. scutula*, a herbicolous taxon which includes, in his opinion, also the foliicolous *Hy. caudatus*. Since Dennis did not mention any blackening of the substrate, the studied specimens on petioles seem to belong to *Hy. caudatus* rather than *Hy. aesculi*.

#### ***Hymenoscyphus vacini*** (Velen.) Baral & E. Weber, Bibliotheca Mycol. 140, p. 121 (1992) – **Figures 20**–**22**

**Figure 20.**
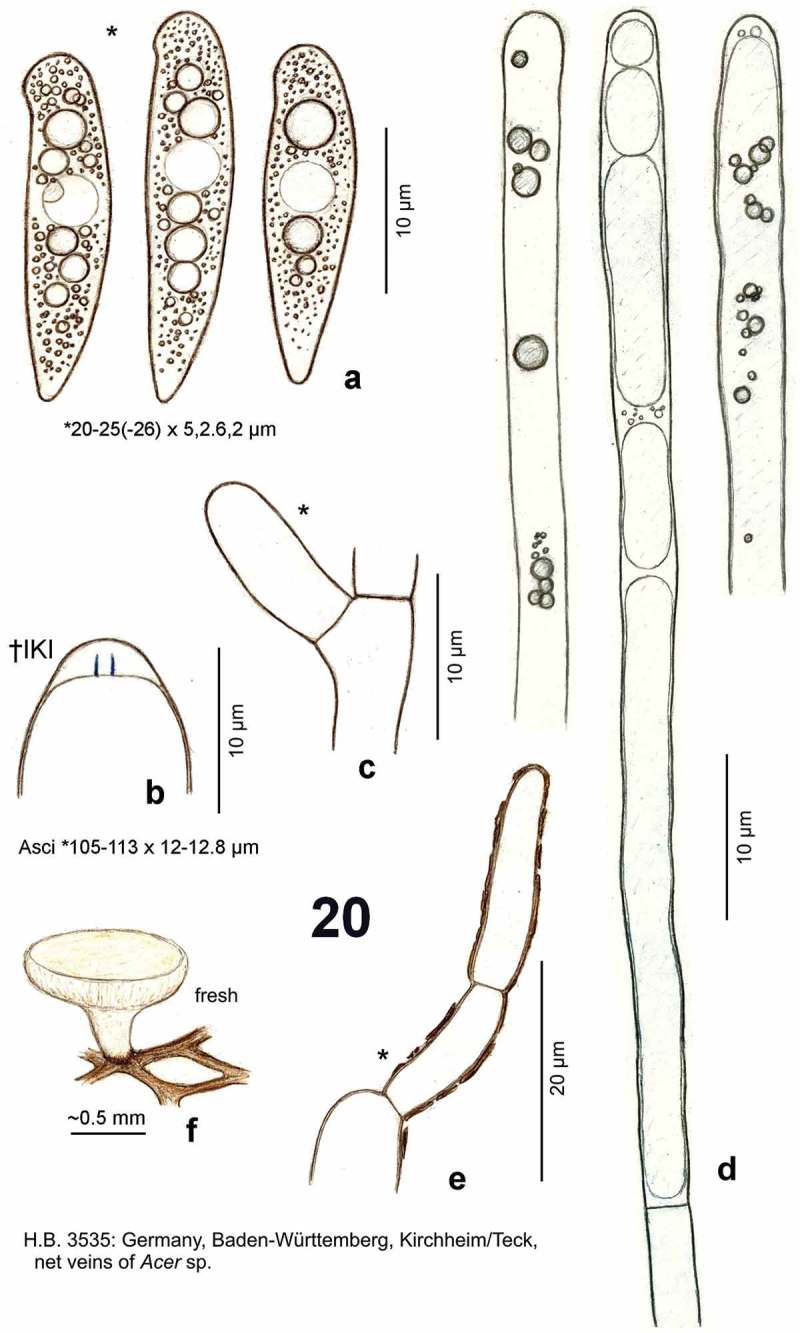
*Hymenoscyphus vacini*: a. ascospores (freshly ejected, containing large and small LBs); b. ascus apex with euamyloid apical ring (*Hymenoscyphus*-type); c. simple-septate ascus base; d. paraphyses containing elongate, low-refractive vacuoles, partly with scattered internal globose VBs of high refractivity; e. cortical hypha of ectal excipulum covered by olive-brown exudate; f. apothecium emerging from net veins of *Acer* leaf. – Living state (except for b).

**Figure 21.**
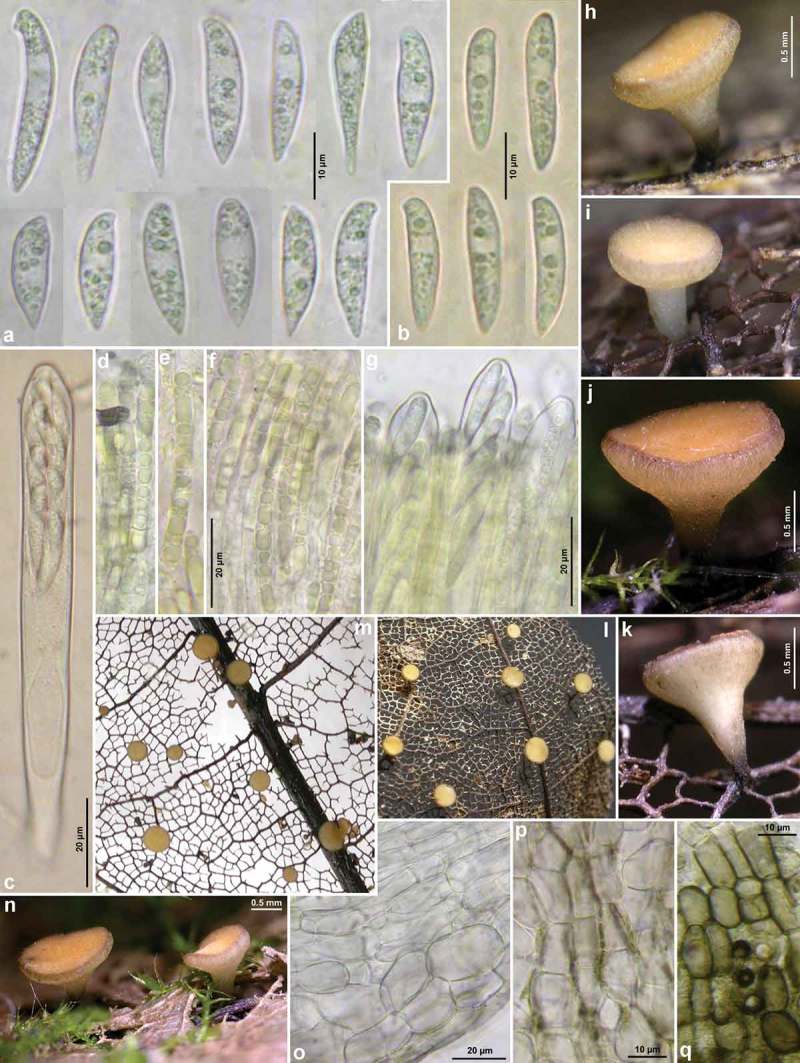
*Hymenoscyphus vacini* (on leaves of *Acer*): a–b. freshly ejected ascospores (containing LBs); c. mature turgescent ascus; d–f. paraphyses (containing pale yellowish VBs); g. mature turgescent asci projecting beyond paraphyses; h–n. fresh apothecia; o. ectal excipulum at lower flanks in median section; p. ectal excipulum at mid flanks in surface view, with olive-brown exudate; q. do., towards base of stipe. – All in living state. – a, c–k, m–q. H.B. 9590 (DE-ST, Merseburg); b, l: H.B. 8296 (DE-BY, München).

**Figure 22.**
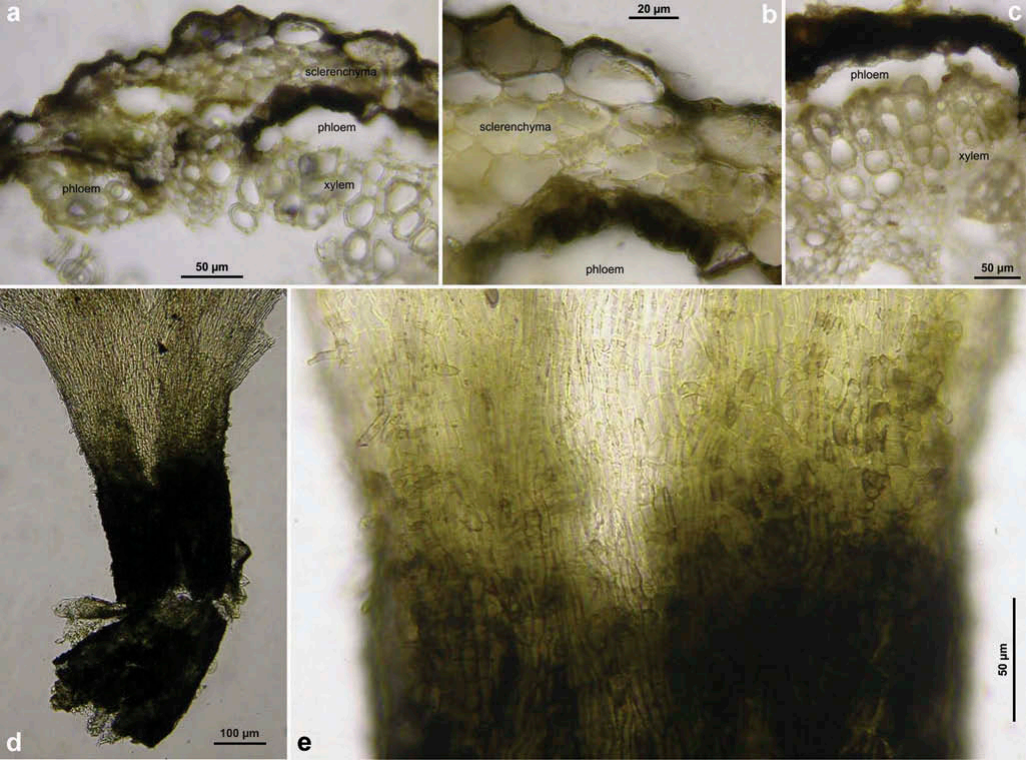
*Hymenoscyphus vacini*: a–c. cross section through cortical region of net vein (in H_2_O), showing black demarcation line surrounding the sclerenchyma on outer and inner face (cortical parenchyma above and phloem beneath being entirely decomposed); fungal hyphae visible inside cells; d–e. apothecial stipe in surface view (slightly squashed, in KOH). – a–c. H.B. 9590 (DE-ST, Merseburg); d–e. H.B. 456 (DE-BW, Stuttgart).

≡ *Helotium vacini* Velen., Novitates mycologicae, p. 185 (1940)
≡ *Lanzia vacini* (Velen.) Spooner, in Kirk & Spooner, Kew Bull., p. 570 (1984)

##### Etymology

Named after the collector, V. Vacek.

##### Holotype

Czechia, South Moravia, Žarosiče, Čtvrtý Žlíbek, veins of *Acer platanoides*, 21.VIII.1939, V. Vacek (PRM 148882; not seen).

##### Apothecia

Moist (0.5–)0.7–1.5(–2)((–3)) mm diam.; disc whitish or pale cream, turing yellowish-ochraceous, slightly concave to flat, margin thin, ± smooth, exterior whitish to pale cream, often densely covered by very fine brownish fibres; stipe 0.2–0.5(–0.8) × 0.2–0.4 mm, concolorous with receptacle, partly translucent, mostly strongly blackened at the base, erumpent from sclerenchyma-phloem layer; dry yellowish-ochraceous. **Asci** *(90–)100–115(–122) × (9.5–)10.5–12(–12.8) µm {6}, †90–110 × 10–11 µm {1}, *6–13 µm longer than paraphyses, 8-spored, spores *obliquely biseriate, pars sporifera *54–67(–71) µm long, tardily or rapidly ejecting spores in water mount; **apex** (†) conical, dome †2–2.5 µm thick, apical ring occupying lower 1/2–2/3 of dome, IKI medium to strongly blue (bb), apically abruptly fading, *Hymenoscyphus*-type {6}; **base** short and broad, arising from simple septa {7}, never with a subbasal protuberance. **Ascospores** *(19–)20–25(–28)((–34)) × (3.7–)4.5–5.5(–6.5) µm {9}, †20–25 × (3.5–)4–4.5(–5) µm {2}, mostly very distinctly scutuloid, cylindrical to strongly fusiform, apex rounded to obtuse, with a slight to strong lateral protrusion, base strongly attenuated, often acute, rarely tail-like, straight to slightly inequilateral or curved, containing 3–10 medium-sized LBs [1–1.8(–2.5)((–3)) µm diam.] and many small ones in each half {10}, lipid content 4–5; sheath not observed, setulae absent; wall surface CRB–; overmature 1–2-septate, with hyaline or pale yellow wall, lipid content low. **Paraphyses** apically uninflated, terminal cell *(30–)37–70(–95) {2} × (3–)3.5–5(–6) {4} µm. **Ectal excipulum** hyaline, at flanks of t. prismatica-angularis oriented at a low angle to the surface, cells broadly brick-shaped, *15–30 × 12–18 µm {2}, externally partly covered by 5–8 µm wide hyphae (somewhat undulating near margin); near base of stipe of ± thick-walled, angular to globose cells; on receptacle and stipe with radial lines or scattered patches of bright olivaecous-brown rough exudate (also on cortical hyphae near margin), near base of stipe black-brown. **VBs** in paraphyses very faintly to medium (rarely strongly) refractive, hyaline to pale amber-yellow, round or angular to very elongate, extending (30–)40–60(–85) µm from tip, low-refractive vacuoles partly containing a number of scattered or grouped, globose, strongly refractive VB-guttules 0.5–2 µm diam. {4}; VBs partly turning red-brown in IKI. **Crystals** absent in complete tissue including stipe base. **Anamorph**: unknown.

##### Habitat

*Aceri-Fraxinetum, Galio-Carpinetum, Pruno-Fraxinetum, Salicetum*, on fallen previous year’s entirely skeletonized leaves, on primary, secondary and net veins (upper and lower face) of *Acer* sp. {5}, *A. platanoides* {2}, *A. pseudoplatanus* {16/1}. **Assoc.**: *Lachnum rhytismatis* {1}. **Desiccation tolerance**: After 2 days in the dry state, some cells of ectal excipulum at flanks and many spores still alive. **Geology**: limestone, gneiss, Knollenmergel. **Phenology**: VIII–IX(–X). **Altitude**: 25–905 m.

##### Characterization

*Hymenoscyphus vacini* differs from *Hy. albidus* s.l. in rather large ascospores that contain a few medium-sized and many small LBs in the living state, also in apothecia with brownish external fibres, a comparatively short stipe with a dark brown base, and a strict occurrence on entirely skeletonized leaves of *Acer*.

##### Nomenclature and circumscription

Spooner (in Kirk & Spooner 1984, p. 570, fig. 11) gave a detailed description based on three British records on leaves of *Acer pseudoplatanus*. For one of them, he stated the substrate to be skeletonized. In a section of the stipe base and the vein beneath, he figured a small but distinct, black internal stroma that surrounds the vascular bundle and the sclerenchyma of the vein. Although Spooner noted that ‘there is no clear stromatic development visible on the substratum surface’, the dark brown external appearance of the veins in the here reported specimens is clearly due to the black internal stroma, which is located below the cortical parenchyma. With its demarcation line surrounding both sides of the sclerenchyma (Figure 22), this stroma corresponds to the type of pseudosclerotium observed in *Hy. aesculi*.

Spooner saw a close resemblance in spore shape with typical species of *Hymenoscyphus*, but he followed the current opinion to consider a dark stroma, though superficially invisible, as a family character of the Sclerotiniaceae (today Rutstroemiaceae). Because of the ectal excipulum with its thin-walled prismatic cells covered by a cortical layer of brown- and rough-walled hyphae and the hyaline spores, Spooner transferred the species to *Lanzia*. Independently hereof, also Svrček (Svrček 1985, p. 182) placed the species in *Lanzia*. When doing this superfluous combination, Svrček referred to a manuscript in press on that species (‘New or less known Discomycetes. 14. Čes. Mykol. 40’, 1986). However, neither *Lan. vacini* nor *Lan. aesculi*, to which he also refers, is mentioned there. Maybe he refrained from this report when being aware of Spooner’s contribution to *Lan. vacini*. His illustration of the holotype (Svrček 1985, pl. 17 fig. 5) fits very well the present specimens.

Collections of *Hy. vacini* were reported as an unnamed species by the first author (in Baral & Krieglsteiner 1985, p. 123), who compared it with *Hy. caudatus* and *He. fraternus* (Peck) Dennis, taxa with often longer, basally not darkened stipes. The latter grows on non-blackened petioles of *Acer* in North America (White 1942, p. 173, as *Helotium fraternum*). When Korf and Zhuang (1985) transferred *Hymenoscyphus serotinus* (Pers.: Fr.) W. Phillips to *Lanzia*, they believed that this species is not specifically distinct from *Hy. vacini*; i.e., they took *Hy. serotinus* in a wide concept that includes both wood and leaves as host.

##### Specimens included

All on skeletonized leaves of *Acer pseudoplatanus*, in a few cases *Acer* sp. or *A. platanoides*.

**B** **elgium**: **West Vlaanderen**, 7 km ENE of Wingene, 3.7 km NNW of Ruiselede, 1 km W of Kruiskerke, Vortebossen, 25 m, 11.XI.1997, B. Declercq (B.D. 97/101, d.v.). – **Germany**: **Sachsen-Anhalt**, 17 km WSW of Merseburg, 2.8 km WSW of Albersroda, Müchelholz, 190 m, 20.VIII.2011, P. Rönsch (H.B. 9590). – **Baden-Württemberg**, 9 km ENE of Karlsruhe, 2 km SW of Weingarten, Weingartener Moor, 115 m, leaves of ?*Acer pseudoplatanus*, 16.IX.1985, W. Winterhoff (W.W. 85315). – 6.5 km W of Stuttgart, 1 km W of Solitude, 500 m, leaves of *Acer* sp., 21.IX.1976, H.O. Baral (ø). – 5 km WNW of Stuttgart, 2 km S of Weilimdorf, Möglinger Stellerain, 380 m, 13.IX.1975, H.O. Baral (H.B. 456). – 6 km NW of Stuttgart, ~2 km NW of Feuerbach, Lemberg, ~360 m, 20.IX.1975, H.O. Baral (ø). – ibid., 12.X.1975, H.O. Baral (ø). – ibid., 29.IX.1976, H.O. Baral (ø). – ibid., 20.IX.1975, H.O. Baral (ø). – 5.5 km NE of Tübingen, NE of Pfrondorf, Gähklinge, 410 m, 5.IX.1988, H.O. Baral (H.B. 3552). – 5.5 km WSW of Reutlingen, S of Ohmenhausen, Löcherwiesenweg, 430 m, 16.IX.1989, H.O. Baral & E. Weber (ø). – 9 km SSW of Reutlingen, 2.3 km ESE of Gönningen, Gönninger Seen, 600 m, 16.IX.1989, H.O. Baral (ø). – ~1.7 km N of Kirchheim u. Teck, Hohes Reisach, ~380 m, leaves of *Acer* sp., 25.VIII.1988, L.G. Krieglsteiner (H.B. 3535). – 8.5 km ENE of Radolfzell, 1.4 km NNW of Langenrain, Teufelstal, 430 m, 25.VIII.1976, H.O. Baral (ø). – 9.7 km ENE of Radolfzell, 1.4 km ENE of Langenrain, Marienschlucht, 450 m, 24.VIII.1976, H.O. Baral (H.B. 782). – 8.3 km NE of Radolfzell, 3.3 km SE of Bodman, SE of camping area, 410 m, 25.VIII.1976, H.O. Baral (ø). – 8 km NE of Radolfzell, 1 km SE of Bodman, S of castle, 430 m, 18. & 19.VIII.1976, H.O. Baral (H.B. 781a, 781bø). – 3.8 km NE of Radolfzell, 1.6 km SE of Möggingen, SW of Mindelsee, 410 m, 28.VIII.1976, H.O. Baral (ø). – **Bayern**, 14 km NNE of Amberg, 1 km N of Hirschau, Frühmess, 450 m, 27.IX.1991, H.O. Baral & E. Weber (ø). – Bayerischer Wald, 12 km SE of Zwiesel, ~4.5 km NNE of Spiegelau, Lärchenberg, S-slope of Rachel, ~890 m, leaves of indet. angiosperm, 5.IX.1989, N. Luschka (REG 374, vid. E. Weber). – 9.5 km SE of München, 1 km E of Neuperlach, Kieswerk, Putzbrunner Str., 545 m, leaves of *Acer* sp., 27.VIII.2006, B. Fellmann (H.B. 8296). – **Austria**: **Salzburg**, 7.5 km WSW of Salzburg, 1 km SW of Wals, Saalach-Altarm, 437 m, leaves of *Acer* sp., 15.V.1993, W. Dämon (W.D. 11/93). – **Steiermark**, 4 km WNW of Gröbming, 0.8 km NW of Weyern, Lend, 905 m, 5.IX.2009, G. Friebes (ø, d.v.). – **Czechia**: **South Moravia**, ~30 km SE of Brno, near Žarosiče, Čtvrtý Žlíbek, ~300 m, veins of *Acer platanoides*, 21.VIII.1939, V. Vacek (PRM 148882, **holotype**, d.v.). – ibid., veins of *A. pseudoplatanus* and *A. platanoides*. 27.VIII.1946 (PRM 683912, n.v.).

### Key to species of Hymenoscyphus *that form a dark stroma in leaves (rarely twigs) of* Acer, Aesculus, Fraxinus, *and* Picrasma (see also Table 3)

**Common features**: apothecial stipe with a brown to black base that emerges from a subepidermal black stroma; asci with euamyloid apical rings; ascospores always heteropolar (scutuloid), with a high lipid content, without setulae.

1. Ascospores *(19–)20–24(–28) × (3.7–)4.5–5.5(–6.5) µm, ±fusoid-clavate, containing several medium-sized LBs 1–1.8(–2.5) µm diam. and many small ones; living paraphyses with low- to medium refractive, pale yellowish-amber VBs; apothecia externally brownish striate or punctate; on net veins of skeletonized leaves of *Acer*, Europe ***Hy. vacini***
  – Ascospores *(13–)14–20(–24) × (3.7–)4–5(–5.5) µm, containing 3–6 large LBs 1.5–3(–4) µm diam. and some small ones; paraphyses with medium to high-refractive, hyaline VBs; apothecia externally whitish or only faintly brownish punctate; on petioles, rachises, primary or secondary veins of other hosts 2
2. On *Aesculus*; medullary excipulum in stipe base without crystals; living spores often with a swollen middle part (crystals and swollen spores uncertain for *Hy. honshuanus*) 3
  – On *Fraxinus*; medullary excipulum in stipe base always containing crystals; living spores never with a swollen middle part 4
3. Asci arising from croziers; spores †3.8–5 µm wide; apothecia yellowish, stipe 1–4 mm long; ectal excipular cells at flanks †5–10 µm wide; Japan ***Hy. honshuanus***
  – Asci arising from simple septa; spores †(3–)3.3–3.8(–4) µm wide; apothecia whitish, stipe 0.25–2 mm long; ectal excipular cells at flanks †10–20 µm wide; Europe, Japan ***Hy. aesculi***
4. Asci arising from simple septa 5
  – Asci arising from croziers 6
5. Ascospores *(13.5–)14.5–18.5(–20.5) µm long; apothecia (0.8–)1–2.5(–4) mm diam.; on *Fraxinus excelsior*, fruiting VII–IX, Europe ***Hy. albidus***
  – Ascospores *13.3–16 µm long; apothecia 0.6–2.8 mm diam.; pseudosclerotium linear, 0.5–0.8 mm wide, on one side of the rachises of *Fraxinus platypodia*, fruiting IX, Japan ***Hy. linearis***
6. Crystals in stipe base mostly aggregated in druses; spores *(15–)16–21(–22) µm long; apothecia (0.7–)1–6(–8.5) mm diam.; on *Fraxinus* spp., fruiting VI–IX, Eastern Asia but introduced to Europe ***Hy. fraxineus***
  – Crystals in stipe base singly; spores †15.5–18.7 µm long; apothecia 0.6–2 mm diam.; on *Picrasma quassioides*, fruiting VIII, Eastern China ***Hy. albidoides***

**Table 3.** Survey of some selected ecological and morphological features of the five here treated foliicolous species of *Hymenoscyphus* with a black stroma (ascus and spore size in Asian *Hy. fraxineus* after description in Hosoya et al. (1993) and a drawing by J.G. Han personal communication).

	*Hy. albidus*	*Hy. fraxineus*	*Hy. fraxineus*	*Hy. honshuanus*	*Hy. aesculi*	*Hy. vacini*
Distribution	Europe	Europe	E-Asia	E-Asia	Europe, [E-Asia]	Europe
Host	*F. excelsior, F. angustifolia*	*F. excelsior, F. angustifolia*	*F. mandshurica, F. rhynchophylla*	*Aesculus turbinata*	*Aesculus hippocastanum*	*Acer pseudoplatanus*
Phenology	(VI–)VII–IX(–X)	(V–)VI–IX	VII–IX	VIII	VII–X	VIII–IX(–X)
Period of record	1850–2012	2006–2014	1990–2011	1961	1942 – 2012	1939–2006
Altitude [m]	3–1690	3–1120	23–1325	~500–800	3–700[–1325]	115–905
Ascospores [µm)	*(14.5–)15–18.5(–19.5) × (3.8–)4–4.7(–5)	*(15–)16–20(–22) × (3.7–)4–4.5(–5)	†14–21 × 3.2–5	†15–20.3 × 3.8–5(–5.4)	*(14–)15–20(–21.5) × (3.7–)4.2–5(–5.2)	*(16–)20–24(–29) × (3.7–)4.5–5.5(–6.5)
Asci [µm]	*91–102 × 10–11.2	*97–135 × 10–12.7	†96–126 × 8–10	†85–94 × 8.5–9	*86–104 × 9–11.5	*90–113 × 9.5–12.8
Croziers	–	+	+	+	–	–
Apoth. [mm]	(0.8–)1–2.5(–4)	(0.7–)1–6(–8.5)	(0.5–)1–2.5(–3.3)	~1.5–3.5	(0.3–)0.6–1.5(–2)	0.6–2

### Remarks on teleomorph and anamorph features of the treated species

#### Apothecial colour

Except for the blackish base of the stipe and sometimes some brownish dots on the receptacle, fresh apothecia of *Hy. albidus, Hy. fraxineus*, and *Hy. aesculi* appear as macroscopically pure white, which is reflected by the specific epithet of *Hy. albidus*. The white colour is astonishing since, under the microscope, the surface of the receptacle and stipe in *Hy. albidus* and *Hy. fraxineus* was often covered by a pale to bright golden-yellow-ochraceous, though very thin exudate.

In the dry state, the apothecia attain also macroscopically a pale to bright yellowish-cream to orange colour, which is partly due to this exudate, but also to oxidation of the VBs inside the paraphyses and in the cortical hyphae that cover the ectal excipulum. For instance, Svrček (1985, p. 132) stressed that fresh apothecia of *Hy. albidus* turn rust-coloured when bruised, a colour change for which these vacuolar contents are responsible.

In *Hy. aesculi* a striking colour change of the receptacle, particularly the hymenium, towards olivaceous-grey to blackish was observed in rehydrated material (Figures 16(g), (j)–(k), 17(j)–(l), 18(o)–(p), 18(c)–(d)), which was not found to be mentioned in the literature, except for those specimens from *Aesculus indica* misidentified as *Hy. albidus* by Sharma (1991). Svrček (1989, p. 73) merely stated the pure white apothecia did not turn reddish. The bright olivaceous-brown pigment (Figure 11(f)) is located in the cytoplasm, especially in the upper part of the paraphyses, and likewise seems to originate from an oxidative chemical change of the VBs in the dead state.

#### Size

Apothecial disc diameters were found to vary strongly within a species. In those on leaves, the diameter seems to depend mainly upon whether the apothecia emerge from a thick petiole or from thin veins. Although *Hy. vacini* grows on thin net veins of skeletonized leaves, this species is able to produce rather large apothecia.

In *Hy. fraxineus* a tendency towards larger apothecia in comparison with *Hy. albidus* is confirmed in the present study (see Tables 1 and 2). A clear trend towards larger apothecia in *Hy. fraxineus* (maximum 3–7 mm diam.) was reported by Kowalski (2012) in a table, while Queloz et al. (2011) stated that they could not observe any difference in apothecial size between the two species. The disc diameter of *Hy. albidus* is given in the literature (fresh or rehydrated) as 1–2 mm (Desmazières), up to 2 mm (White, Dennis), 1–2.5 mm (Arendholz), 1–3 mm (Breitenbach & Kränzlin), 1.5–3 mm (Boudier), or 2–4 mm (Srvček). Stipe length is reported as about 1–1.5(–2) mm. This is in concordance with our observations, and gives evidence that this species rarely exceeded 4 mm in diam. Only Patouillard indicated a larger size (3–5 mm), and also Kirisits and Kräutler (2013, fig. 1) observed diameters of up to 5 mm in a collection from the Bretagne. *Hy. fraxineus* may reach a size of about 8 mm as a maximum, but is often also found with rather small apothecia.

Online photos of Slovenian records under the name *Hy. albidus* (by N. Ogris, Ljubljana, http://www.zdravgozd.si/prirocnik/zapis.aspx?idso=319 – accessed 23 May 2014) show exceptionally long stipes with red-brown lower half. Possibly, the long stipes result from keeping the collection in a moist chamber in a dark room.

#### Stroma

A character which has all here treated species in common concerns the dark brown or black, melanized pseudosclerotial plate outside of the sclerenchyma of petioles, rachises, and veins that they inhabit. This stromatized host tissue was reported already in the type of *Hy. albidus* by Desmazières (1851), who thought that it might be an *Asteroma*, an anamorph connected mainly to members of the family Valsaceae (Diaporthales). However, there is no doubt that the stroma or pseudosclerotium belongs to the *Hymenoscyphus*.

Sections through petioles show that the apothecial initials are formed from mycelium inside the pseudosclerotia (Figures 3(a), 3(e), 8(f)). In pure culture on modified Seed agar medium, ‘at first a whitish-grey mycelium was formed, which changed soon to dark brown or black and pseudostromatic areas with a hard surface were developed’ (Pham et al. 2013, p. 447). Contrary to Svrček (1985), who stated that not all apothecia of *Hy. albidus* emerge from the blackened areas (‘epidermis’), we have never seen apothecia inserted on unblackened parts of the substrate in any of the here treated species.

What appears dark brown to black by macroscopical view is a thin layer (demarcation line or pseudosclerotial plate) in the outer region of the host tissue, which completely surrounds the infected parts beneath, and which separates them laterally from uninfected regions. This dark pigment of the pseudosclerotial plate completely fades when placed in eau de Javelle for about 5 min, as is generally observed with melanized pigments.

In all these foliicolous species, the pseudosclerotial plate develops beneath the epidermis within the large-celled cortical parenchyma, often at the border to the smaller-celled, thick-walled sclerenchyma. During winter the epidermis and cortical parenchyma and also the phloem are degraded and may entirely disappear (Figure 17(m)–(n), 17(s)), but remnants of brown cortical parenchyma or phloem are partly still present when apothecia are formed (Figures 11(k), 19). Only in rare cases the light-coloured epidermis can still be found loosely attached to the dark stroma beneath by covering large areas of the rachis (Figure 11(c)). The dark stromatic colour originates from black-brown hyphae that form a plectenchyma (textura epidermoidea) on both sides of the host cell walls of the large-celled cortical parenchyma and the outer cells of the sclerenchyma (Figure 3(e)). The lumina of the host cells are densely filled with hyaline or pale brown hyphae (Figures 3(d), 3(f)–(g), 8(i)–(k), 11(h)). The same is true in the host tissue below the apothecial stipe (Figures 3(a), 3(e), 18h). Cells of the sclerenchyma distant from the demarcation line are only sparsely filled with hyphae, lining the inner face of their walls (Figure 8(g), 18(i)).

In *Hy. aesculi* and *Hy. vacini* also the inner border of the sclerenchyma is frequently delimited by a black pseudosclerotial plate when viewed in cross section (Figures 17(m), 17(q)–(s), 19(b), 22). However, also those cells below the inner pseudosclerotial plate contain small amounts of hyphae.

Demarcation lines that delimit the pseudosclerotium from uninfected tissue are infrequently seen in *Hy. albidus* and *Hy. fraxineus* when studying cross-cuts of rachises. Especially when several infections took place within a single leaf, the black lines frequently cross outer or inner parts of the mark parenchyma (Figure 11(i)). Also Gross, Zaffarano et al. (2012) observed up to eight different infections and mating type locus (MAT) genotypes in a single rachis colonized by *Hy. fraxineus*, although it looked homogeneously blackened from outside. A limited (insular) pattern of pseudosclerotia more obviously permits to conclude that a multiple infection of a single rachis took place.

The present study suggests that differences in the extension of the pseudosclerotium occur between *Hy. albidus* and *Hy. fraxineus*. The apothecia of *Hy. albidus* often grow on sharply isolated (insular) black areas on the otherwise pale straw-coloured rachis, though in some specimens, including the type, the stroma covers a main part of the rachises. *Hy. fraxineus* often stains more or less the entire rachis of a leaf in black, or a major part of it, though also not rarely only insular areas. In *Hy. aesculi* more or less the entire petiole was found to be occupied by the pseudosclerotium.

Various literature reports of *Hy. albidus* account for this peculiarity by phrases such as ‘on blackened areas’ or ‘on blackened patches’ (White 1944; Dennis 1956, 1978; Arendholz 1979; Clark 1980; Breitenbach & Kränzlin 1981; Ellis & Ellis 1997). Also Patouillard’s (Patouillard 1885, pl. 382), Boudier’s (1908, pl. 492), and Gams and Arnold (2007, fig. 1A) illustrations show this insular pattern. The difference in the stroma between *Hy. albidus* and *Hy. fraxineus* is also recognizable on the two macrophotos of these species in Hietala and Solheim (2011). Likewise, on a photo of rachises from a montane locality without ash dieback in the Austrian Alps (Kirisits 2011, fig. 6), the partial blackening can clearly be seen, but these petioles were not genetically processed and their identity is uncertain. Overview photos of *Hy. fraxineus* show entirely blackened rachises in virtually all of the many illustrated papers on ash dieback (e.g., Engesser, Forster et al. 2009; Engesser, Meierb et al. 2009; Kirisits 2010; Metzler 2009, 2010; Solheim et al. 2011; Kowalski et al. 2013).

Above the black stipe base a constriction is often seen where it intergrades with the whitish main part of the stipe (Figures 7(n), 8(c), 8(f)). The black basal part of the stipe represents the primordium, from which the development of the stipe and later the receptacle starts. The primordia can be seen as minute blackish spikes on the stromatized rachises. Apparently they appear only in late spring shortly before apothecia are formed.

The black pseudosclerotial plate is not yet present when the wilted leaves fall to ground in autumn but is formed prior to the winter season (see Gross & Holdenrieder 2013). Since the fallen rachises lie loosely on the forest floor, they completely dry down during dry weather. The authors found that the mycelium in the pseudosclerotia survives at least 92 days in the dry state, while in nature under repeated drying and rewetting the pseudosclerotia can endure at least 2 years.

#### Croziers and simple septa

Differences in the ascogenous hyphae among closely related species play an important role in the taxonomy of ascomycetes. A couple of papers have drawn attention to this peculiarity, such as Huhtinen (1990) for *Hyaloscypha*, Baral (1996, p. 255) and Hengstmengel (1996, p. 192) for *Hymenoscyphus*. Nevertheless, croziers and simple septa are still being frequently neglected in many groups.

Literature reports on the mode of ascus base development in *Hymenoscyphus* species with a pseudosclerotium are sparse. According to White’s (1944, p. 608, figs. 19–24) precise illustration of Pl. Crypt. N. France, éd. I. n° 2004 (FH), the asci in the lectotype of *Hy. albidus* arise from simple septa. White’s statement ‘not originating from croziers’ in the description allows to assume that he saw this feature also in the examined isolectotype collection in FH (éd. II. n° 1604). Besides Arendholz (1979), White’s report is the only known to us from the past century, which mentions the type of ascus development in this species.

V. Ruiz-Badanelli (in Hairaud 2010) and Zhao et al. (2013) presented photographs of croziers of *Hy. fraxineus* (as *Hy. pseudoalbidus*). Hairaud gave also a photo of the simple-septate ascus base in *Hy. albidus* for his collection from July 2012, following personal communication with H.O. Baral. However, his note that he saw the feature also in the 2007 sample must be in error. A statement by Otto and Wagner (2011) about the difference in the ascus base between the two species originates also from such personal communication with the first author of the present paper.

*Hy. honshuanus* (≡*Lambertellinia scutuloides*) is here found to possess croziers (Figure 12(b)), although the protologue states the contrary. Korf and Lizoň (1994) observation was not accompanied by an illustration; however, it remains open whether this observation was erroneous, or the type collection represented a mixtum.

#### Homothallism, monokaryon, relative DNA content of nuclei

The cytological background of the presence vs. absence of croziers is still little understood. In *Hy. albidus* the absence of croziers is correlated with homothallism and with the absence of an anamorph state (see below under ‘Anamorph and life cycle’ section). Data on the karyological situation in the ascogenous hyphae of the here treated pseudosclerotium-forming species of *Hymenoscyphus* were presently not available.

Berkson (1966) observed in *Chaetomium* that species with croziers had dikaryotic ascogenous hyphae whereas others with simple septa were monokaryotic. Also Weber (1992, p. 68) observed monokaryotic ascogenous hyphae in species of Helotiales that lack croziers, although only in a smaller part of them, while in others monokaryotic and dikaryotic cells occurred mixed in the same ascogenous system. Nuclei of monokaryotic cells thereby had the double DNA content compared to those of dikaryotic cells of the same species. In species in which the ascogenous hyphae possess croziers, Weber observed mainly dikaryotic cells. Zickler et al. (1995) reported ‘uninucleate croziers’ in mutant strains of *Podospora anserina* which normally were dikaryotic. However, their microphotos (Figure 4(a)–(d)) show simple-septate ascogenous hyphae with a partial presence of hook-like protuberances which did not fuse with the cells below. The complete croziers drawn for the uninucleate mutants on their diagrammatic survey (Figure 5(b)–(c)) are not seen on their micrographs.

In her study on the relative DNA content of nuclei in vegetative cells, Weber (1992) revealed for *Hy. albidus* a value of 4× of the basic value (1×), the lowest found in the Helotiales. Within *Hymenoscyphus*, this value of 4× was not rarely encountered in her study (e.g., in *He. scutula* and *Hy. vacini*), but values of 2×, 3×, 6×, and rarely 1× also occurred. A correlation of these values with the presence or absence of croziers could not be established; however, species with croziers as well as simple septa occurred in quite equal frequency within each value, except for those species with values of 1× (simple-septate) and 2× (croziers), which were too sparsely encountered in order to draw a conclusion. In any case, it would be interesting to measure the relative DNA content of the two so far not investigated species of *Hymenoscyphus* with a pseudosclerotium, *Hy. fraxineus* and *Hy. aesculi*. A survey on the known genome size values in fungi are found in Kullman et al. (2005).

A correlation between croziers and apothecial size similar as in *Hy. albidus* and *Hy. fraxineus* was noted in two further species pairs of the Helotiales (Baral in Weber 1992, p. 110, 118): (1) *Calycina herbarum* (without croziers) and *C.* aff. *herbarum* (with croziers and larger apothecia), a very similar species which was currently confused with *Ca. herbarum*, and which inhabits a comparable spectrum of herbaceous stems; (2) *Heyderia pusilla* (without croziers, on needles of *Pinus*) and *Hy. cucullata* (=*Hy. abietis*, with croziers and larger fruit bodies, on needles of *Picea*). Within both species pairs the relative DNA content did not differ (*Calycina* 2×, *Heyderia* 6×).

#### Apical ring

The morphology of the ascus apical thickening in the dead expanded state when stained with iodine is usually very similar among the true members of the large genus *Hymenoscyphus*. Its characteristic morphology was addressed by Baral (in Baral & Krieglsteiner 1985, p. 119), and later referred to as *Hymenoscyphus*-type (Baral 1987, p. 126, fig. 10–12, Verkley 1993). The ascus apex in the Sclerotiniaceae and Rutstroemiaceae (*Sclerotinia*-type) sharply differs hereof and easily permits distinction with the light microscope between sclerotiniaceous/rutstroemiaceous and hymenoscyphoid taxa in most cases (see Baral 1987). White (1944) and Svrček (1985) illustrated the *Hymenoscyphus*-type of apical ring in *Hy. albidus*. However, it was perhaps more because of a similar spore shape that White believed in a very close relationship to *He. scutula* and *Hy. caudatus*.

#### Ascospore size and shape

Heteropolar, subapically rounded and with a lateral hook-like protrusion, basally ± pointed ascospores are typical of many of the true members of *Hymenoscyphus*. This characteristic spore type was called ‘scutuloid’ by Baral (in Baral & Krieglsteiner 1985) according to the situation in the representative species *He. scutula*, but was rarely also referred to as *virguliformis*, which means in Latin comma-shaped, e.g., by Patouillard (1885, p. 173), who erroneously wrote in French ‘spores virgultiformes’ instead of ‘virguliformes’. The spore size in the type material of *Hy. albidus* was given as minimum 15 µm by Desmazières, 11–17 × 3.5–4 µm by Nylander (1868), 15–18 × 3.5–4 µm by Karsten (1871), 15–18 × 3–4 µm by Massee (1895), and 13–17 × 4–5 µm by White (1944).

*Hy. fraxineus* is said to have slightly longer ascospores, but variation and overlap in this feature forbid recognition of the two species on *Fraxinus* from spore length alone. Queloz et al. (2011) gave a graphic representation of length values of some selected specimens. Accordingly, spore length ranges were (11–)13–**14–16**–17(–18) µm in *Hy. albidus* and (15–)16–**17–18**–20(–22) µm in *Hy. fraxineus*, both possibly evaluated from dead spores. The tendency to longer spores in *Hy. fraxineus* is confirmed from the specimens studied by us. Our spore length values are about 0.5–1 µm over those of Queloz et al. (2011), probably because of a slight shrinking effect between living and dead spores. The longest freshly ejected spores that we measured in *Hy. fraxineus* were almost 25 µm long.

The characteristic spore shape of *Hy. aesculi* with a swollen middle part is only present when living spores are studied in a water mount. After releasing the spore turgor by heating or applying killing agents, the spores shrink and more or less completely loose this inflation (see Figure 18(n)–(o)). Also inside the turgescent asci, the spores are distinctly narrower and less swollen in their middle part because of the ascus turgor. When discharged in a water mount, the spores swell to their typical shape within a few seconds.

#### Ascospore contents

Published illustrations of living spores of the here presented species are rather rare. Particularly freshly ejected living spores, but also those inside fully turgescent asci, show a regular pattern of larger and smaller oil drops (LBs). *Hy. albidus, Hy. fraxineus*, and *Hy. aesculi* possess quite the same pattern of rather large, globose LBs (2–3.5 µm diam. in *Hy. albidus* and *Hy. fraxineus*, 1.5–2.5 µm in *Hy. aesculi*), about 1–4 in each half, surrounded by small ones. The spores of *Hy. vacini* consistently contain only medium-sized LBs (~1–2 µm diam.) among many small ones.

Patouillard’s (1885, pl. 382) drawing, though rather small and inexact, appears to be almost the only published documentation of living mature ascospores within the *Hy. albidus* aggregate, showing about four rather large globose drops in each spore. Two recent illustrations of living spores are found in Hairaud (2010) and Gross, Holdenrieder et al. (2014, fig. 1h). Even Boudier (1908, pl. 492), who consequently studied ascomycetes in the fresh state, illustrated the living spores of *Hy. albidus* with only small- to medium-sized drops, the latter about 1.2–1.8 µm diam., though with a rather regular pattern. Perhaps theses spores were not fully mature.

The original lipid pattern can often also be observed in herbarium material, but it requires caution to select those spores which possess enough maturity, and to avoid all those which are overmature or in which the LBs have fused to irregular aggregations. Actually, most reports in the literature concern dead spores that contain irregular rather variably shaped aggregations.

Ascospore reports such as ‘often with a large oil globule’ (Dennis 1956, p. 93) or ‘often with 1–3 oil drops’ (Arendholz 1979, p. 80) are due to fusion of the original oil drops during the influence of lethal mountants or the natural death of spores. Surprisingly, White’s 1944, fig. 24) drawing of the type of *Hy. albidus* shows a rather regular spore content with a ‘granular’, multiguttulate sporoplasm, the largest LBs exceeding not even 1 µm diam. This is astonishing, since oil drops usually fuse in dead material, but never split into small ones. The explanation for this appears to be that White (1943, p. 137) followed a method described by Martin (1934, p. 264), who placed fungal fragments in alcohol before applying KOH and phloxine. We made a test with a specimen of *Hy. fraxineus*: the large oil drops in the spores indeed disappeared and only the small ones remained visible. Apparently it is the alcohol that dissolves the large oil drops (see Figure 10(h_3_)→(h_4_)).

#### Ascospore sheath and setulae

Distinct setulae at the spore ends were not observed in any of the species treated here, while a delicate sheath around ejected spores was occasionally seen, though not yet in *Hy. honshuanus* and *Hy. vacini*. Because of its fragile nature, it is a rarely reported structure. When studying herbarium material, it remains inevitably undetected since it does not detach from the wall when dead spores are rehydrated, due to the absence of cell turgor. The sheath can only be observed when the spores are ejected from living asci in a water mount: after discharge, they rapidly take up water and swell, and the inelastic sheath bursts and eventually floats besides the spores. Gross, Holdenrieder et al. (2014, fig. 1m–n) reported a thick, hyaline to pale brown mucilage secreted from the spores of *Hy. fraxineus*, by which they are thought to adhere to the leaf surface after wind dispersal. This mucilage was observed on overmature (1-septate or brown) spores during. In fact, the mature spores are hyaline and aseptate during ejection, and soon dehydrated after dispersal, including their mucilage. Possibly the sheath observed by us covers a thin mucilage that gets more abundant during germination and affixes the spore to the leaf when the appressorium is formed.

#### Overmature ascospores, pigmentation

Apparently all true members of *Hymenoscyphus* eject hyaline, non-septate ascospores. However, at an overmature stage of development, the spores frequently get 1(–2)-septate and their wall may turn pale to light brown and often also warted. Because brown or septate spores of a *Hymenoscyphus* are never forcibly ejected, such spores in this genus can only be found inside dead asci or outside asci, but never inside living asci (see also Baral 1992, p. 375).

The presence of pigmented spores has been overestimated as a generic character in the Helotiales. As pointed out by Galán and Baral (1997, p. 61) and Hengstmengel (2009, p. 271), this feature of overmature spores is quite a common character in *Hymenoscyphus,* though it is seen only inconsistently and sometimes very rarely in a given species. The proportion of brown spores in a preparation depends on the senescence of the apothecia. For some reason, perhaps unfavourable field conditions, some populations produce many such brown spores when senescent, while in others none or only a few can be found.

Spore germination is observed in both hyaline and brown spores. Possibly, the hyaline spores directly infect the living leaves, whereas the brown spores are resting spores being able to survive a longer time. Brown germinating spores were figured by Hosoya et al. (1993) for *Hy. fraxineus* (as *Lam. albida*), and by Kowalski and Holdenrieder (2009, fig. 1g). In the present study, brown spores were only two times seen in *Hy. fraxineus* (Figure 10(f)), once in *Hy. honshuanus*, and once in *Hy. aesculi*.

#### Ectal excipulum

In sections of living specimens of *Hy. albidus*, the ectal excipulum is a textura prismatica with comparatively thin though slightly gelatinized walls. In dead specimens, or when adding killing agents such as KOH, the cells shrink especially in width, and the gel between the walls becomes more obvious due to imbibition. The shrinking effect was probably the reason why Svrček (1985) found the cells to be only 4–5 µm wide, though he obviously did not see any gelatinization. Likewise, Arendholz (1979), who classified the ectal excipulum of *Hy. albidus* as a t. intricata, did not see glassy or agglutinated cell walls. White (1944, fig. 22) found the excipular cells in the lectotype 8–10(–15) µm wide, but did not mention or clearly figure a gel between the cell walls.

In *Hy. fraxineus* strong variation in the width of the excipular cells was noted, which appears to depend mainly on the development stage: in younger apothecia the ectal excipulum forms a t. prismatica-porrecta in both stipe and receptacle, with a cell width of *~6–10 µm, whereas in older and larger apothecia it forms a t. prismatica-angularis with *~10–30 µm wide cells.

#### Hair-like protrusions

Svrček (1985) mentioned the outside of the receptacle of *Hy. albidus* to be finely downy, the stipe almost tomentose in the lower part. Also Dennis (1956, p. 94) described and illustrated ‘short, slender, obtuse hairs about 10–15 × 2 µm’ on the excipular surface in this species. We here describe them in *Hy. albidus* and *Hy. fraxineus* (Figure 9(l)–(m)), but saw a pubescent stipe also in *Hy. aesculi*. These protrusions were not present in each of the studied samples of these species, however.

#### Vacuolar bodies

In all here treated species of *Hymenoscyphus*, refractive vacuolar bodies (VBs) occupy most of the lumen of the terminal cells of the living paraphyses and partly also of the cortical cells of the marginal ectal excipulum. In young paraphyses, they are globose or somewhat angular, but soon fuse and finally form very elongate vacuoles (see also Baral 1992, p. 363). In the dead state, they are distorted or entirely disappeared. Also KOH applied to living paraphyses irreversibly makes them disappear (Baral 1992), and that without provoking any colour reaction. Staining agents when added to a water mount, provoke a distinct stain to VBs: CRB slowly stains them turqoise, and IKI light yellowish to bright red-brown. In IKI mounts, many minute red-brown granules may extrude into the surrounding medium, but this effect was only inconsistently obtained in *Hy. albidus, Hy. fraxineus*, and *Hy. vacini*, and not in *Hy. aesculi*.

A colour change of the VBs from hyaline towards olivaceous-brown was repeatedly noted in *Hy. aesculi*, resulting in partially blackish-brown hymenia in herbarium material. In the other species studied, the VBs turned merely yellowish-cream with age. The phenomenon appears to be due to an oxidative chemical process.

#### Crystals

Arendholz (1979) observed abundant crystals of ‘presumably Calcium oxalate’ in the lower 1/4 of the stipe. We have regularly seen these crystals in both *Hy. albidus* (Figure 3(c)) and *Hy. fraxineus* (Figures 8(d)–(e), 9(h)–(i), (k)), and Zheng and Zhuang (2014) report them for *Hy. fraxineus* and *Hy. albidoides*. Although crystals occur also inside some of the cells of the infected rachises, those found in the dense tissue of the medullary excipulum of the stipe base are undoubtedly formed by the fungus, not by the host.

The crystals are hyaline, ±rhomboid, and frequently aggregated in druses. They are KOH-inert and do not stain with CRB or IKI. Under polarized light, they are birefringent by changing the direction of the light for about 90° (Figure 9(i)). Crystals occur also in the living rachises of *Fraxinus*, inside cells of the radial rays between the vascular bundles. However, these are not arranged in druses. Crystals were not observed in *Hy. aesculi* and *Hy. vacini*, nor in any other species of *Hymenoscyphus* examined by us.

#### Anamorph and life cycle

*Hy. fraxineus* is heterothallic and produces a *Chalara* anamorph in pure culture, whereas the avirulent *Hy. albidus* is homothallic (Gross, Zaffarano et al. 2012) and not associated with an asexual state (Kirisits et al. 2013; Kirisits & Kräutler 2013); also it displays slower growth on agar media.

Various authors have shown that the conidia of *Ch. fraxinea* do not germinate. Their perhaps exclusive function as spermatia was suggested by various authors, including Gross, Zaffarano et al. (2012; see also Gross, Holdenrieder et al. (2014)), whereas distribution seems to be exclusively accomplished by ascospores. The whole life cycle from leaf infection via ascospores to production of apothecia lasts only 1 year (Gross, Zaffarano et al. 2012, fig. 5). Yet, the pseudosclerotia in the fallen leaves may produce apothecia also in the second year (Gross & Holdenrieder 2013). Moreover, seeds of *Fraxinus* may contribute to dispersal: in 8.3% of the investigated seeds DNA of *Hy. fraxineus* could be detected (Cleary et al. 2012).

According to Gross, Zaffarano et al. (2012), the formation of spermatia in *Hy. fraxineus* is linked to the heterothallism of that species. The authors found that *Hy. fraxineus* needs different mating types in order to produce apothecia, whereas *Hy. albidus* is homothallic (self-fertile) and able to fruit from a single-spore mycelium (A. Gross personal communication). Because of its homothallism, we assume that *Hy. albidus* does not require complex mechanisms of nuclear division during the dicaryophase which result in the formation of croziers in *Hy. fraxineus*.

The situation in *Hy. fraxineus*/*Hy. albidus* raises the question whether also in other species of *Hymenoscyphus* the presence/absence of an anamorph is linked to the presence/absence of croziers. *Chalara* anamorphs have virtually been unknown in that genus before the connection was established by Kowalski and Holdenrieder (2009). Unpublished observations of a *Chalara* asexual morph in four species with asci arising consistently from croziers support this correlation. Two of them were observed in ascospore isolates by E. Weber: (1) hyaline phialides in a species close to *Phaeohelotium imberbe* (H.B. 7469); (2) light olivaceous phialides in *Hymenoscyphus infarciens* (H.B. 7025a, CBS 122016). The other two concern hyaline to pale brown phialides emerging from the ectal excipulum at the flanks of the receptacle (Baral in preparation): (3) in the lectotype of *Hy. subferrugineus*; (4) in a specimen identified as *Hy. calyculus* (H.B. 3128). *Hy. linearis*, however, a species with simple-septate asci, was found to produce a *Chalara* asexual state in culture.

Conidia of *Hy. fraxineus* are abundantly produced during autumn and winter on recently infected rachises, after leaf fall at low temperatures, preferentially near pseudosclerotial plates (Kowalski & Bartnik 2010; Gross, Holdenrieder et al. 2014). The pale to light olivaceous-brown phialides have a size of (13–)15–24(–28) × 3.5–5(–5.7) µm, with a 7–10(–12.5) µm long and (2.1–)2.5–3.1 µm wide collarette, and the phialoconidia measure *3–4(–4.2) × (1.7–)2–2.3(–2.5) µm, the first formed conidia being much more elongate, *(5–)6–7(–9) × (1.7–)2–2.5(–2.8) µm (Kowalski 2006; Floreancig 2009; Ogris et al. 2009; Flajšman 2011; Shabunin et al. 2012).

Size of conidiophores (13.5–18.5 × 4–5.5 µm) and conidia (2.5–3 × 1.5–2 µm) as given by Hosoya et al. (1993) for Japanese material is at the lower end of the range, except for the inflated basal part of the conidiophores which are more inflated (ventricose).

### Remarks on ecological aspects of the two treated species on *Fraxinus*

#### Altitude

Apothecia of *Hy. albidus* were recorded in the region of the Alps up to an altitude of 1690 m as indicated by the sample from Giswil (Switzerland, NMLU 2309-80) made in 1980 (Queloz et al. 2011). Before *Hy. fraxineus* started its invasion, *Hy. albidus* showed a wide but scattered distribution in lowland regions of Europe, where it became nowadays extinct, except in those regions of Western and Southwestern Europe which have not yet been invaded. In 2009 *Hy. albidus* was still present in montane regions of Switzerland, according to Queloz et al. (2011): in southern parts of the country at altitudes of 600–1075 m only *Hy. albidus* could be found, whereas *Hy. fraxineus* was in that year exclusively present in the northern lowlands at 345–570 m, although some *Hy. albidus* records were made also there at altitudes of 508–695 m.

Kirisits (2011, fig. 5) presented for *Hy. fraxineus* a map of Eastern Tyrol (Austria), with records of ash dieback by July 2010 at elevations of about 650–720 m in the Drava valley near Lienz, whereas the disease did at that time not yet occur in other parts of Eastern Tyrol, including localities at higher elevations (e.g., in the Gail valley). However, observations made in 2014 indicate that apothecia or dieback symptoms occurred already at 1120 m in the Prättigau (Graubünden, H.O. Baral), at 1130 m in the Hürital (Rosalmig, Zug, U. Graf personal communication), at 1150 m in the Gesäuse National Park (Ennstaler Alpen) and 1300 m in Kärnten (G. Koller personal communication), and at up to 1600 m in the Virgental (Eastern Tyrol) at the uppermost altitudinal limit of *F. excelsior* (T. Kirisits personal communication). This suggests that *Hy. albidus* is threatened in the entire distribution range of ash in Central Europe. In Eastern Asia the observed maximum altitudinal records of *Hy. fraxineus* were 1325 m in Japan, 1100 m in South Korea, and 1310 m in Northeast China (Jilin).

#### Host range

Within Europe, *Hy. fraxineus* was recorded, apart from the European temperate species *F. excelsior*, also on the North American *F. nigra* (Estonia, Drenkhan and Hanso 2010) and on two submediterranean species from Southern and Southeastern Europea, *F. angustifolia* and *F. ornus*. Artificial wound inoculation experiments revealed an equally high or slightly higher susceptibility of *F. angustifolia* to *Hy. fraxineus* compared to *F. excelsior* (Matlakova 2009). When inoculated, the fungus also caused symptoms on *F. ornus*, though less intensely than on the two other species (Matlakova 2009, Gross, Holdenrieder et al. 2014). However, ash dieback symptoms due to natural infections have so far never been observed on flowering ash, neither on woody parts nor on leaves, and this species may be resistant to the fungus (Gross, Holdenrieder et al. 2014; T. Kirisits personal communication). Gross, Holdenrieder et al. (2014) reported the occurrence of apothecia of *Hy. fraxineus* on petioles of *F. ornus*, which may indicate that it occurs endophytically in leaves of this ash species.

*F. nigra* was badly affected with symptoms, *F. pennsylvanica* only moderately, and *F. americana* and *F. mandshurica* were least affected (Drenkhan & Hanso 2010). In its presently known original area of Eastern Asia, the hosts of *Hy. fraxineus* are *F. mandshurica* and *F. rhynchophylla* (partly treated as a subspecies of *F. chinensis*).

*Hy. albidus* is known with certainty only from *F. excelsior* and sometimes *F. angustifolia*. For two here mentioned records on *Acer*, voucher specimens have not been preserved. A preserved specimen on a leaf of ‘*Pyrus*’ from Belgium was found to grow in fact on *Fraxinus*. Misidentifications of either host or fungus explain in some cases a seemingly wide host spectrum.

Similarly narrow host spectra are noted in the genus *Rutstroemia*. The common *Rutstroemia longipes* is said to be confined to rachises of *Fraxinus* in North America (R.P. Korf personal communication), and the closely related *Rutstroemia luteovirescens* is frequent on petioles of *Acer* in Europe. However, the independence of these two fungal species and the extent of their host spectra have been questioned in the past (see above).

#### Fruiting on twigs

As an exception, both *Hy. albidus* and *Hy. fraxineus* were found fruiting on twigs of *Fraxinus* in the present study, though in both cases also petioles were inhabited. The record of *Hy. albidus* (Echternach, LUX 047701) concerns a corticated 1 mm thick twig with blackened bark, while in that of *Hy. fraxineus* (Luzern, H.B. 9571) the apothecia grew on blackened wood of an almost entirely decorticated, 5.5 mm thick twig. The rare occurrence of *Hy. fraxineus* on twigs was repeatedly reported, e.g., by Gross, Holdenrieder et al. (2014, fig. 1d). The rareness of this behaviour is astonishing, since living twigs are regularly invaded by the fungus. Whether also *Hy. albidus* is able to invade living woody parts is not known.

#### Phenology

The slightly earlier production of apothecia of *Hy. fraxineus* in comparison with *Hy. albidus* is shown in Table 4, based on the specimens listed in our collection data. Literature data suggest that *Hy. albidus* occurred also in October (e.g., Krieglsteiner 1999, p. 238, 30.X.1997, near Schweinfurt). A fruiting period of *Hy. fraxineus* during May–October(–November) was demonstrated by Chandelier et al. (2014, fig. 4) using spore traps and real-time polymerase chain reaction (PCR). This earlier start of fruiting plays a role in the dominance of the pathogen over *Hy. albidus*.

**Table 4.** Phenology of *Hy. albidus* (35 collections) and *Hy. fraxineus* (55 collections) within Europe, based mainly on data in Tables 1 and 2. Indicated is the number of collections for each species.

	May 1	May 2	June 1	June 2	July 1	July 2	Aug. 1	Aug. 2	Sept. 1	Sept. 2	Oct. 1	Oct. 2
*Hy. albidus*	–	–	–	2	4	8	7	5	7	3	–	–
*Hy. fraxineus*	–	3	2	10	15	15	6	7	7	2	–	–

#### Distribution and replacement

**Figure 23.**
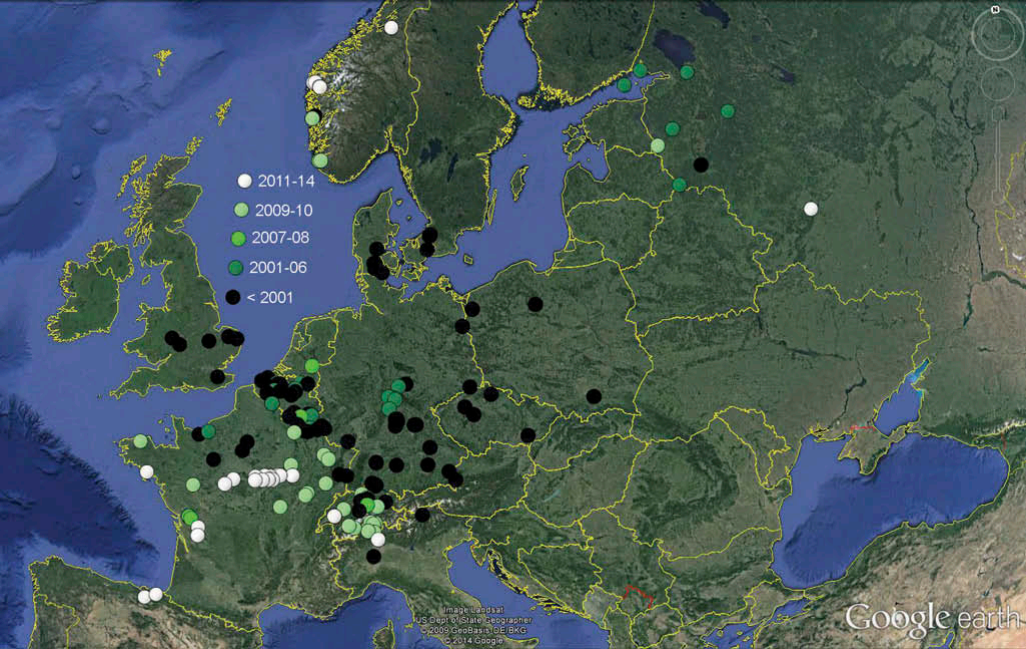
Distribution of *Hymenoscyphus albidus* within Europe according to trustable records known to us (those for which data about the host and the presence of a stroma were not available are omitted, also all those doubtful records which fall in the period of invasion by *Hy. fraxineus*).

**Figure 24.**
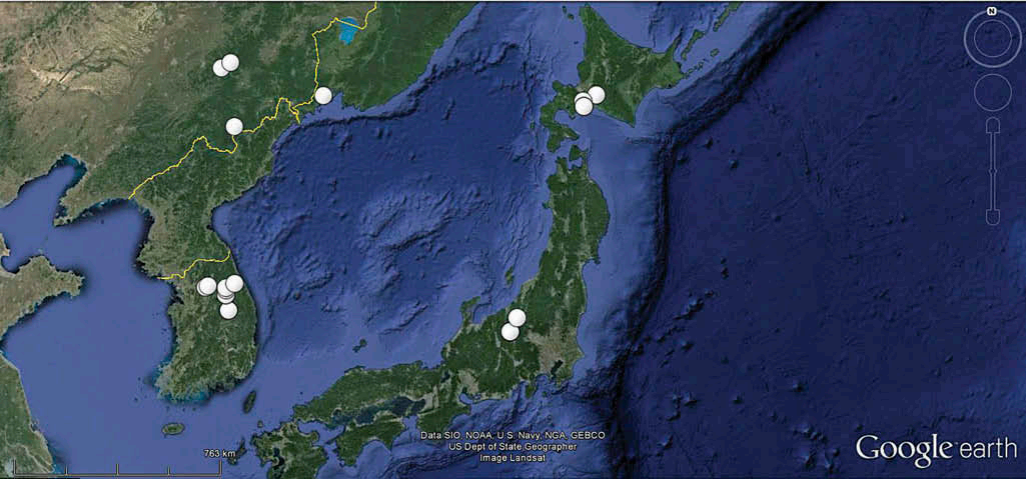
Known records of *Hymenoscyphus fraxineus* in Eastern Asia.

The presently known original distribution of *Hy. fraxineus* is shown in Figure 24. Whether it covers the entire natural area of its known host trees *F. mandshurica* and *F. rhynchophylla* is not known. The original distribution of *Hy. albidus* appears to comprise all more or less temperate to montane but also oromediterranean regions of Europe, where *F. excelsior* or *F. angustifolia* occur. A rather dense distribution at least in Central and Western Europe is evident, based on the presently available data (Figure 23).

*Hy. fraxineus* is a neomycete within Europe and threatens other fungi in that ecological niche similarly as introduced plants do with the native vegetation. Our estimation is that particularly host-specific fungi such as *Hy. albidus* or *Cy. fraxinophila* are severely threatened by the invasive fungus.

The area-wide extinction of *Hy. albidus* within Central Europe from ca. 2000 onwards is obvious, due to the mass colonization of the rachises by *Hy. fraxineus*. As a matter of fact, the species could not be discovered anymore in several countries of Central, Northern, and Eastern Europe, the last known records dating from ca. 1990–2008 (see also below and Figure 23). The species could so far maintain only in high-montane areas, e.g., in the Alps, and in lowlands outside the present distribution area of *Hy. fraxineus*, particularly in the southwestern part of France. A history of the spread of *Hy. fraxineus* along with the extinction of *Hy. albidus* is given, e.g., by Bakys (2013) and Vasaitis (2013).

*Cy. fraxinophila* fruited quite abundantly in late autumn (IX–XII) all over Central Europe. A total of 36 records in the first author’s database refer to the period 1975–1995, whereas only two concern later years (2006 and 2009, Mecklenburg). Despite a thorough search for the species, it could not be collected in the last years in the area of Tübingen. However, O. Koukol (personal communication) observed *Cy. fraxinophila* in 2013 at several sites within Czechia, although *Hy. albidus* disappeared there during the invasion by *Hy. fraxineus*. It can be assumed that *Hy. fraxineus* does not infect all living leaves of an ash tree, so there probably remain enough uninfected leaves that fall to the ground during autumn. Infection of these leaves by *Cy. fraxinophila* is assumed to take place on the ground during the spore discharge period in Sept.–Dec.

The predicted decline of the European ash might also affect lignicolous and other fungi. Jönsson and Thor (2012) discussed the extinction risk for the epiphytic lichen diversity on bark of living ash trees, with 174 species recorded on the island of Gotland (Sweden).

### Historical records and present distribution of *Hymenoscyphus albidus* and *Hymenoscyphus fraxineus*

When excluding misidentifications, *Hy. albidus* was predominantly considered as rare in earlier times. The species was sometimes regarded as common, particularly in England and France, but some of these records concern misidentifications on substrates other than *Fraxinus. Hy. fraxineus* existed originally only in Eastern Asia (Japan, Korea, northeast of China, and far east of Russia). The first records of apothecia within Europe known to us were made in 1999 in Lithuania. With the invasion by *Hy. fraxineus, Hy. albidus* became extinct in most regions of Europe (see Figure 23).

#### Japan

Apothecia recorded in Japan on blackened rachises of *Fraxinus mandshurica* var. *japonica* were identified as *Lam. albida* by Hosoya et al. (1993). The very detailed documentation includes a pure culture and an anamorph which fits very well *Ch. fraxinea*. A sequence (ITS and part of SSU) was deposited at the NBCR (National Biological Resource Center) of the Japanese National Institute of Technology and Evaluation under the number NBRC102368 and was, therefore, overlooked by European researchers. After re-examination of eight specimens collected during 1990–2011, these were found to possess croziers at the ascus base in each of the apothecia tested (see Table 2). Five of these were sequenced and confirmed to belong to *Hy. fraxineus* (Zhao et al. 2013).

#### Korea

J.G. Han (personal communication) examined 10 records on rachises of *Fraxinus mandshurica* and *F. rhynchophylla* morphologically, four of them also genetically. All were found by him to possess croziers, and his sequences refer them to *Hy. fraxineus*.

#### China (northeast)

Zheng and Zhuang (2014) recorded *Hy. fraxineus* on *F. mandshurica* at four localities in the northeast of China (Jilin province). The authors observed croziers at the ascus base, and their sequences refer these records to *Hy. fraxineus*.

#### Russia (far east)

A collection of *Hy. fraxineus* from Kedrovaya Pad Reserve (Khasansky District, Primorsky Kray) on *F. mandshurica* was made in 17.VIII.2005 by E. Popov (personal communication), identified by the presence of croziers (1508-8-Ked). Marčiulynienė et al. (2013) confirmed the presence of *Hy. fraxineus* from three sites near Primorye by molecular methods.

#### Russia (westernmost part)

Records of *Hy. albidus* were made by E. Popov (personal communication) during 2004–2006 from Leningrad Oblast, e.g., from two islands in the Gulf of Finland. Moreover, several finds from Pskov (1998–2009) and one from Kaluga Oblast (2013) were made by him, as well as one from Novgorod Oblast (Valdaysky district, 4.IX.2001, Popov 2005). All these were re-examined by him recently and the absence of croziers was confirmed (documentation not seen). E. Popov observed that *Hy. albidus* fruited usually very abundantly, and almost everywhere in stands where *Fraxinus* grows. Only in the Volga-Akhtuba floodplain, where *F. pennsylvanica* was very commonly introduced, he did not observe *Hy. albidus*.

In a forest with *Alnus incana* and *F. excelsior* in Pskov Oblast visited by E. Popov every year since 1996, with *Hy. albidus* being common, no symptoms of disease were obvious until 2009 when all ash trees showed strong dieback, all dying after 1 or 2 years, also in several similar communities within a radius of 5 km. Other regions (Kaluga and Orel Oblast) did not show the disease so far. Shabunin et al. (2012) recorded *Hy. fraxineus* southwest of St. Petersburg (Leningrad Oblast) in 2012 by molecular methods.

#### Poland

Several records of *Hy. albidus* can be found in Polish publications. For instance, Lisiewska and Bujakiewicz (1976, p. 57) mentioned a specimen (as *He. robergei*) for the Dębina reserve in Wielkopolska (Central Poland), and Bujakiewicz (2001, p. 116) reported *Hy. albidus* from the Ostrów Panieński reserve close to Chełmno (Northern Poland) collected during 1981–1984. Molecular data have not been gained from these specimens, but the occurrence on blackened rachises prior to ca. 1990 leaves little doubt about their identity.

Ash dieback symptoms are known from Northern Poland since 1992 (Kowalski 2006), and since 1998 decline of *F. excelsior* occurred all over the country (Vasaitis 2013). Nevertheless, apothecia were noticed as part of the disease only since 2008 (Kowalski & Holdenrieder 2009). During surveys in 2005–2006 and 2011, *Hy. fraxineus* had totally replaced *Hy. albidus*, according to molecular analyses of 230 apothecia-derived cultures which all represented *Hy. fraxineus* (Kraj et al. 2012; Kowalski et al. 2013).

#### Lithuania

Six herbarium specimens under the name *Hy. albidus*, collected on blackened petioles of *F. excelsior* from the districts of Kėdainiai and Vilnius in 1999–2002, were recently reviewed by E. Kutorga (personal communication). He could demonstrate the presence of croziers by microphotos in all of them. The distribution of ‘*Hy. albidus*’ in Lithuania based on specimens (not tested for croziers) and literature data was reported by Treigienė et al. (2010) for the districts of Kėdainiai, Ukmergė and Biržai during field trips in 1997–2005, but these observations most likely also concern *Hy. fraxineus*.

The ash dieback disease was noticed since about 1996/97 (Juodvalkis & Vasiliauskas 2002), mainly in Biržai and Panevėžys districts (Northern Lithuania), Kėdainiai, Jonava and Ukmergė districts (Central Lithuania, see also Stepanenkova (2010) and Bakys (2013)). In the following years it spread over the whole country. A DNA sequence that matches *Hy. fraxineus* (AY787704) was for the first time gained in 2001 by Lygis et al. (2005, as *Hymenoscyphus* sp. 970, later corrected to *Ch. fraxinea* in GenBank, V. Lygis/E. Kutorga personal communication) from a stem base of *F. excelsior* in Biržai district (northeast of Lithuania). Mass production of apothecia was noticed by E. Kutorga from about 1999 onwards including 2013.

#### Estonia and Latvia

Rytkönen et al. (2011) isolated *Ch. fraxinea* from *F. excelsior* in 2008 in NW- and SE-Estonia and in 2009 in W-Latvia. Also Drenkhan and Hanso (2010) isolated the fungus in 2009 in SE-Estonia. However, the first indications of ash dieback were as early as 1995 in the northwest of Estonia, where the disease reached most ash stands during 2003, and from where it spread towards the southeast of the country during 2006–2007 (Drenkhan et al. 2014, fig. 4). Also in Latvia massive ash dieback occurred since mid-1995s (Vasaitis 2013).

#### Finland

Karsten (1871, p. 112) stated that collections from his country were unknown to him. The first record of *Ch. fraxinea* was in 2007 and 2008 in the SW-part of the country (Åland archipelago and mainland), from where the disease spread towards the southeast. The first symptoms of ash disease were in 2000 in the Åland archipelago (Rytkönen et al. 2011).

#### Sweden

*Hy. albidus* is known from a single locality near Helsingborg in Skåne (south of Sweden), where it was abundant in 1994 and 1996 (S.Å. Hanson personal communication). The first observation of the disease was in 2001, and in 2004 the entire distribution area of *F. excelsior* in the south of Sweden was affected (Timmermann et al. 2011). A frequent occurrence of apothecia was first noted in Skåne in 2002 and particularly from 2004 onwards (S.Å. Hanson personal communication).

#### Norway

*Hy. albidus* was recorded in 1985 from the southwest of Norway (Bergen) by S. Olsen (K. Homble personal communication), and is today still present along the west coast, according to molecular data by Hietala and Solheim (2011). Possibly it had occurred also in the southeast of Norway before *Hy. fraxineus* invaded the country, but K. Homble never found apothecia referable to *Hy. albidus* in the Oslo area before 2009.

The first symptoms were noted in 2006 in the most southern part of the country. The first isolate of *Ch. fraxinea* was made in SE-Norway in May 2008 in a nursery in the Østfold region, and in 2009 the disease has spread along the entire coastline there (Talgø et al. 2009; Timmermann et al. 2011). K. Homble (personal communication) noticed mass occurrence of apothecia in VIII.2012 in Kjaglidalen (Bærum, Akershus, 15 km west of Oslo), while H. Solheim (personal communication) observed the first mass occurrence in 2009 near Ås (30 km S of Oslo).

Hietala and Solheim (2011) found by molecular methods that *Hy. albidus* survived along the west coast of Norway. According to Hietala et al. (2013, p. 5), ‘The low ascospore number of *Hy. albidus* detected by real-time PCR in our Norwegian ash stand could relate to such a transition. On the other hand, our data suggest that *Hy. albidus* is still present in the studied forest.’.

#### Great Britain

Berkeley and Broome (1878, p. 29, No. 1724) list a single record of *Hy. albidus* located in Southeast England, with the data ‘On ash petioles. [Kent,] East Farleigh, Sept. 13, 1876’. The same record is mentioned by Cooke (1878, p. 127). Also Phillips (1887, p. 138) gave only one though very remote West English record ([Shropshire,] Shrewsbury, Copthorne). Dennis (1956, p. 93, as *He. robergei*) based his description on two British specimens, the one mentioned by Berkeley, and one in the east of England: Norfolk, Wheatfen [SE of Norwich], 27.VII.1946.

Clark (1980) recorded *Hy. albidus* rather often on black patches of *Fraxinus* petioles (but also on *Aesculus*) in Warwickshire (West Midlands of England), with the first record in 1967. Ellis and Ellis (1997, p. 137, fig. 600) probably relied on Clark’s records when saying that *Hy. albidus* is common during July–October. A distribution map is shown in Webber and Hendry (2012), which includes also misidentified records, especially on substrates other than *Fraxinus*.

The British Isles were considered as being free of *Hy. fraxineus* until January 2012 when ash seedlings were imported from the Netherlands and Germany. At four locations in England they showed the typical signs of the disease (Webber & Hendry 2012). In August 2012 *Ch. fraxinea* was reported as well from Scotland in a stand of young ashes planted in spring 2009 (Webber & Hendry 2012; Munro 2012). Mainly the eastern part of the country was affected.

An intensive survey carried out since November 2012 confirmed 427 sites with ash trees infected by *Ch. fraxinea* (status 25.III.2013). Southeastern areas of the British main island are affected the most (http://www.defra.gov.uk/news/2012/11/09/wms-ash/), but confirmed sites are distributed from Wales to Scotland and Ireland.

#### Denmark

A couple of collections of *Hy. albidus* have been made between 1989 and 1999, some of which were DNA-checked (McKinney et al. 2012, T. Laessøe personal communication). According to the molecular analysis, other collections made in 2005–2010 turned out to belong always to *Hy. fraxineus*, including three sites where *Hy. albidus* apothecia had been collected previously, and it was concluded that *Hy. fraxineus* had eliminated *Hy. albidus* in these regions.

The first records of ash disease were in 2002 near Haderslev (a small port on the Baltic side of the Jutland peninsula), and in 2003 in Zealand and Bornholm (Thomsen et al. 2011; Bakys 2013).

#### The Netherlands

Arnolds et al. (1999) considered *Hy. albidus* as a comparatively rare species, though rather common in the Ijsselmeerpolders and Zeeland. A Dutch online database (http://www.verspreidingsatlas.nl/622010) shows indeed numerous records throughout the country, many of which dating before 1990. Only *Fraxinus* petioles are given as substrate. A record made by S. Helleman in Nijmegen (Gelderland) in 2007 is here confirmed to represent *Hy. albidus*.

Ash dieback was first reported in 2010 at young trees in public parks in Bellingwedde in the far northeast of the country close to the German border (EPPO 2010). L. Deceuninck recorded for the first time apothecia of *Hy. fraxineus* in 2012 at Schokland (Flevoland, B. Declercq personal communication), while S. Helleman (personal communication) did not observe occurrence of *Hy. fraxineus* around Boxmeer (Noord-Brabant) until recently (2013).

#### Belgium and Luxembourg

Mouton (1886, p. 140) reported *Hy. albidus* from Beaufays near Liège (on petioles of *Fraxinus*, without any further data). In a preliminary checklist for Flanders, Declercq (personal communication) considered *Hy. albidus* as rather common, based on numerous records collected and identified by Lieve Deceuninck, Bernard Declercq and Piet Bormans during 1989–2012. Also from Wallonia a lot of records were made during 1993–2008 by B. Declercq. For all of them the absence of croziers was noted (B. Declercq personal communication, documentation not seen). A further record from the southeast of Belgium and three from Luxembourg preserved at LUX (all leg. C. Besch, during 1985–1990) were re-examined in the present study and found to represent *Hy. albidus*.

According to Roskams and De Haeck (2011), the first record of ash disease was in autumn 2010 in Eastern Flanders (Hainaut), while in Flemish Brabant it was already present as early as 2007. In 2011 the disease was recorded throughout Flanders. For Wallonia (Southern Belgium) the disease was firstly recorded in June 2010 (Chandelier et al. 2011). Apothecia of *Hy. fraxineus* were detected for the first time in Belgium and Luxembourg in 2012 and 2013 (B. Declercq, G. Marson personal communication, Garnier-Delcourt et al. 2013), and that in great abundance at various sites.

#### France

Roberge’s specimens of *Hy. albidus* around Caen (dépt. Calvados) concern the abundant type collection made around 1850, and a scantier later sample. Cooke (1876, p. 132, pl. LXV fig. 297) and Patouillard (1885, p. 173, pl. 382) gave the first illustrations of the species. While Cooke studied the type material (Desm. n° 2004), Patouillard obviously depicted a recent collection (with living spores) but regrettably did not indicate its origin. Boudier (1908, p. 287, pl. 492) provided a detailed coloured illustration based on a record from Fôret de Carnelle north of Paris (dépt. Val-d’Oise), and stated that the species is frequent in summer and autumn.

A further record was made near Paris on 2.IX.1945 by Arnaud (Gams & Arnold 2007). Here the apothecia (on blackened petiole) were parasitized by the hyphomycete *Pleurocatena acicularis* Arnaud ex Gams & Arnold. From near Besançon a record of *Hy. albidus* was made in 22.VIII.1996 and illustrated by G. Moyne (personal communication).

From 2007 onwards, M. Hairaud (personal communication) observed *Hy. albidus* repeatedly in dépt. Deux Sèvres, especially in Marais Poitevin. Based on a molecular identification, Husson et al. (2011) listed 18 recent records of *Hy. albidus* from Central and West France, one made in 2002 (dept. Calvados), and 17 in 2009 (dépts Côte d’Armor, Haute-Marne, Maine-et-Loire, Saône-et-Loire, Haute-Saône, Moselle, Meurthe-et-Moselle).

According to a map given by Goudet and Piou (2012), the first record of ash disease was in 2008 in dépt. Haute-Saône, though a map in Timmermann et al. (2011) gives 2007 for this region. A photo taken by G. Moyne (personal communication) near Besançon (La Vèze, bois d’Aglans, dépt. Doubs, 13.VIII.2008) showing abundant and rather large apothecia on entirely blackened petioles supports that the disease invaded this part of Eastern France already before 2008. In their molecular study, Husson et al. (2011) identified in the whole northeast of France exclusively *Hy. fraxineus* (collected in 2009), while in Central and West France only *Hy. albidus* was recorded (see above). In 2010 the disease was widespread in Northeastern France, but was also recorded in Northern France, and in 2012 in the northwest (Normandie, Jersey).

#### Germany

When Arendholz (1979, p. 79) performed his study on leaf-inhabiting Helotiales, he had only material of *Hy. albidus* of the nineteenth century at his disposition: the type collection, the material of Berkeley, and a specimen from Germany (Sachsen, Großer Winterberg [Sächsische Schweiz, Elbsandsteingebirge], X.1898, W. Krieger, Fungi Sax. 1486, HBG). Baral and Krieglsteiner (1985, p. 121) reported collections from the south of Germany made during 1975–1979 along the rivers Rhein and Donau, also from Franken and Eifel, but were unaware of records from Northern Germany. Beyer (1992, p. 67) described a single collection made in August near Burggaillenreuth (Bayreuth), and believed the species to be rare. G.J. Krieglsteiner (1993, pl. 745) presented a distribution map for W-Germany that shows a scattered occurrence of *Hy. albidus* within Niedersachsen, Rheinland-Pfalz, Baden-Württemberg, and Bayern, but whether it includes also misidentified samples on other substrates or without pseudosclerotium remains unclear.

L.G. Krieglsteiner (1999, p. 238, 2004, p. 601) reported *Hy. albidus* from 4 localities in the Main area between Schweinfurt and Würzburg (Bayern), and from the Rhön mountains at the edge of Bayern, Hessen and Thüringen with 14 collections. Judging from the phenology data (30.VII.–30.X.) and the occurrence in the years between 1996 and 2004, these records might all concern *Hy. albidus*. The same is true for nine samples from the Hainich National Park made between 16.VII. and 17.XI. in 2003 made by F. Putzmann, G. Hirsch and A. Gminder (P. Püwert & I. Wagner personal communication). Especially those specimens collected in 2003–2004 should be re-examined for croziers in order to exclude *Hy. fraxineus*. However, *Hy. fraxineus* has apparently never been recorded within Thüringen in the years before 2008 (P. Püwert & I. Wagner personal communication); therefore, we strongly suspect that the ash disease was still absent in the Hainich and Rhön region in 2004.

As early as 2002, ash dieback or conspicuous damages on ash were noticed in the northeastern lowlands (Schumacher et al. 2007; Leonhard et al. 2009; Heydeck & Dahms 2012). The first scientific proof of the presence of *Ch. fraxinea* for Germany was published by Schumacher et al. in 2007 and was detected in material collected from woodlands and nurseries, e.g., from Salzwedel (Altmark, Sachsen-Anhalt). The earliest records of mass fructifications that came to our notice were observed in Sachsen (SN, 2006) and Mecklenburg-Vorpommern (MV, 2007) (see Table 2). In Mecklenburg many ash stands have been cut in order to avoid damage of the timber; therefore, the pathogen was suppressed (T. Richter personal communication).

In Thüringen, the fructifications of *Hy. fraxineus* became very abundant from 2008 onwards (P. Püwert & I. Wagner personal communication). Although the ascus base was studied by I. Wagner (personal communication) in only a few collections, the absence of abundant fruit bodies in previous years suggests that all these records from the south of Thüringen (around Sonneberg) but also some from the Rhön mountains collected in 2011 belonged to *Hy. fraxineus*. The presence of *Ch. fraxinea* was confirmed officially for Thüringen as late as 2009 (Baier et al. 2010; TLWJF 2010).

In Baden-Württemberg ash dieback was first noted in 2006 for a single tree in the eastern part of the country (Gaildorf, Metzler 2010, personal communication). The first detection of *Ch. fraxinea* dates from 2009 from near Aalen (Schröter et al. 2009, B. Metzler personal communication), and the disease remained almost undetected in SW-Germany before 2009 (Metzler 2009). At four sites in Baden (along the Rhine between Freiburg and Karlsruhe) dieback symptoms could be backdated to 2007 based on repeated yearly infection, followed by proliferation of replacement shoots (Metzler et al. 2012). The first symptoms detected in the region around Stuttgart (Calw, Ludwigsburg, Heilbronn, Schwäbisch Hall, etc.) were all made in May and June 2009 (B. Metzler personal communication). Apothecia were first recorded near Heidelberg in 2009 (M. Bemmann, unpreserved) and near Tübingen in 2011 (H.O. Baral, Table 2).

For Bayern the disease seems to have started in 2008, being recorded at various sites, especially in the north (Main area) and the southeast to east (Leonhard et al. 2009). Apothecia were first recorded in 2009 by P. Karasch in the southeast, and in 2010 in the northeast by H. Ostrow (P. Karasch personal communication).

#### Austria

Some records of *Hy. albidus* from Oberösterreich are included in the distribution map of Krieglsteiner (1993, pl. 745). A recent map (Dämon et al. 2009) shows 98 records under the name *Hy. albidus*, mainly from the north and east of Austria, and merely 7 of *Hy. fraxineus*. Only a few of the *Hy. albidus* records date before 2000, whilst the majority was made between 2008 and 2009; therefore, probably most of them concern *Hy. fraxineus*. For a critical review of these records and a map see Kirisits and Cech (2009). After the invasion of *Hy. fraxineus, Hy. albidus* has not been observed in Austria anymore (Kirisits & Kräutler 2013).

Ash dieback appears to have invaded Austria from Czechia. The first observations on younger trees date from 2005, while in 2008 symptoms were observed in all Austrian provinces. *Ch. fraxinea* was first isolated in 2007, and apothecia were first detected in 2009 in great number in various parts of the country (Kirisits & Cech 2011).

#### Switzerland and Liechtenstein

Breitenbach and Kränzlin (1981) reported *Hy. albidus* in the cantons Luzern, Obwalden and Nidwalden, and stated the species to be uncommon. Prongué et al. (2004) gave four records for Liechtenstein, and the drawing on our Figure 1 refers to one of them. A recent distribution map of Switzerland (Senn-Irlet 2014) gives four records of *Hy. albidus* before 1990 and eight between 1991 and 2005, but 26 after 2005. However, the records after 2005 certainly include *Hy. fraxineus*. Based on molecular identification, Queloz et al. (2011) reported 24 collections of *Hy. albidus* from the cantons Bern, Glarus, Obwalden, Ticino, Valais, Zug and Zürich, collected mainly in 2009 but also earlier. Most of them originate from montane to subalpine areas in more southern regions of Switzerland (see Figure 23).

The epidemic was not evident before 2007 in Switzerland, and diseased trees were noticed in 2008 only in the north and northwest of the country. During 2009–2011 *Hy. fraxineus* spread towards the Alps (Engesser & Meier 2012; Pautasso et al. 2013). Apothecia of *Hy. fraxineus* were recorded by Queloz et al. (2011) during 2009–2011. In the montane southern cantons Ticino, Glarus and Wallis the disease was still absent during that period.

#### Italy

Rehm (1893, p. 797) mentioned a collection from Südtirol (leg. G. Bresadola), in which apothecia of obviously *Hy. albidus* arise from blackened patches on the petioles of *Fraxinus* (other records in Rehm concern *Cy. fraxinophila*). A record from Vigevano (Pavia) on rotting petioles of *Fraxinus* (22.IX.1983) is included in the checklist of fungi from the ‘Parco della Valle del Ticino’ (Gaggianese et al. 2002).

In NW-Italy along the Italo-Slovenian border, Ogris, Hauptman, Jurc et al. (2010) recorded in July 2009 ash dieback symptoms and isolated *Ch. fraxinea* from one canker.

#### Czechia

Velenovský (1934, p. 205) considered *He. albidum* as frequent and often abundant in whole Bohemia. However, he confused this species with *Cy. fraxinophila* (see below). Although two of his four preserved specimens represent *Hy. albidus* (Central Bohemia: Bilichov 23.VII.1925, PRM 147373; Slané IX.1928, PRM 148933), it can be assumed that his observation of an abundant occurrence was only true for *Cy. fraxinophila*.Two further Czech specimens were deposited by V. Vacek (Cernošice, 12.VII.1942, PRM 683594; Žarošice, 5.IX.1942, PRM 683595). Revision of this material and also that from Bilichov (PRM 147373) done by M. Tomšovský and O. Koukol based on morphology and especially molecular data (see GenBank HF937562.1, HF937561.1, HF937560.1) confirmed the identification as *Hy. albidus*. No material of *Hy. albidus* is present in PRC and BRNM. When searching for *Hy. albidus* since 2009, no records were made of this species which appears to be extinct.

Ash dieback symptoms were observed already in the mid-1990s, and increasingly in 2004 (Jankovský et al. 2008). In Moravia, the first record of *Ch. fraxinea* was in 2007 (Jankovský & Holdenrieder 2009). In most parts of Bohemia and Moravia, *Hy. fraxineus* is now abundant everywhere (O. Koukol personal communication). Collections from Bohemia deposited in PRM are, e.g., from Srbská Kamenice in the north of Czechia (6.VI.2009, PRM 961903, M. Chlebická) and Bílý vrch between Mochov and Celákovice northeast of Praha (8.VII.2011, PRM 899715, M. Kríž). Numerous further collections taken since 2009 from various localities in Czechia belonged exclusively to *Hy. fraxineus*, with the highest observed altitude at 970 m in Southern Bohemia (Ceské Žleby, 17. IX. 2013, leg. V. Poustka, PRC 1806), whereas *Hy. albidus* seems to be extinct (O. Koukol personal communication).

#### Hungary

Bánhegyi (1941, p. 18) reported a collection of *Hy. albidus* on petioles of *Fraxinus* (Budapest, Hüvösvölgy, 12.VII.1940), and Svrček (1979, p. 153) one on blackened petioles and veins of *Fraxinus angustifolia* (PRM 818040, collected by him in 20.IX.1978 during the VII. Congress of European Mycologists in Budapest in the Bugac puszta; a specimen made during this congress is also found in CUP-059500).

Symptoms of ash dieback on *F. excelsior* and isolation of *Ch. fraxinea* were first reported in May 2008 in NW-Hungary (Szabó 2009).

#### Slovenia and Croatia

According to N. Matočec and I. Kušan (personal communication), *Hy. albidus* is not uncommon in montane areas of Croatia. However, *Hy. fraxineus* was observed to have replaced it since about 2010, though in lower rate at altitudes above 1000 m, while it was abundant also at the few studied colline sites. Because these results are part of a funded project, we refrained from including Croatian records in our map of *Hy. albidus*.

Ogris et al. (2009) and Ogris, Hauptman, Bogovi (2010) observed dieback of ash in Slovenia during 2006–2009, and the first records of apothecia in 2009 and 2010 (as ‘*Hy. albidus*’). For Croatia, Barić et al. (2012) reported first records of *Ch. fraxinea* in 2009, and dieback symptoms since 2010–2011 on both *F. excelsior* and *F. angustifolia*, with apothecia observed since 2011.

### Molecular research

The identification of the pathogen is currently accomplished by molecular methods. The used gene regions for species delimitation mainly concern the internal transcribed spacer ribosomal DNA (ITS), the calmodulin gene (CAL), and the translation elongation factor 1-α gene (EF1-α). The nuclear large subunit rDNA (LSU, = 28S) sequence is available so far only for one strain of *Hy. fraxineus* in GenBank (HM145907, Europe) and another in Nite Biological Resource Center (NBRC102368, Japan), whereas the nuclear small subunit rRNA (SSU, = 18S) was rather frequently obtained. The β-tubulin gene is present in GenBank for a single strain of a *Hymenoscyphus* only (*Hy. fructigenus*, FJ477056), but was used by Zheng and Zhuang (2014) to distinguish between *Hy. fraxineus* and *Hy. albidoides*, with a consistent difference at 12 nucleotide positions. Microsatellite markers were developed for studying the population genetics of the pathogen (Gross, Grünig et al. 2012; Gross, Zaffarano et al. 2012). In addition, the MAT was sequenced and analysed to investigate the mating system of *Hy. fraxineus* and *Hy. albidus* (Gross, Zaffarano et al. 2012).

#### Differences between European *Hymenoscyphus fraxineus* and *Hymenoscyphus albidus*

Queloz et al. (2011) defined *Hy. fraxineus* based on 20 nucleotide positions in the CAL gene and 18 positions in the region of the EF1-α gene. At these 38 specifically mentioned, so-called definition positions, *Hy. fraxineus* consistently differed from *Hy. albidus* in the available sequences. Therefore, they were selected to constitute the protologue of the new species (see also Queloz et al. (2012) for corrections to these positions). Four further positions represent gaps that were not considered in the protologue definition (V. Queloz, personal communication). These are pos. 235 in the CAL gene, and pos. 232–234 in the EF1-α gene (the alignment at pos. 232–236 is not at an optimum in the table of Zhao et al. 2013)(Table 5).

However, also in the ITS rDNA region 11 nucleotide positions can be used to distinguish *Hy. fraxineus* from *Hy. albidus*. Based on the presently available data in GenBank, together with the original ITS sequence of Japanese material in NBRC, four sequences published in Shabunin et al. (2012) from Leningrad Oblast, and four gained by V. Queloz (personal communication) from Central European specimens of the first author, European *Hy. fraxineus* differs from *Hy. albidus* in the ITS1 region at positions 77 (T/C), 79 (T/C), 80 (gap/G), 87 (C/T), 101 (T/C), 117 (C/T), and 124 (T/C), and in the ITS2 region at positions 355 (G/C), 361 (T/C), 436 (C/A) and 450 (C/A) (Tables 5 and 6) [position numbers starting with the last five nucleotides of the SSU: CATTA]. One exception must be mentioned: though otherwise perfectly matching *Hy. fraxineus*, a twice sequenced isolate from Czechia (071026.1, FJ429386, GU586921) shows at position 124 the character C which would be typical of *Hy. albidus*, as well as Chinese, Korean and Japanese *Hy. fraxineus*.

Apart from the characteristics in the ITS, Husson et al. (2011, table 4) mentioned a difference of four nucleotides in the partial SSU between the two species: positions 56 (C/T), 90 (C/A), 182 (T/C), and 269 (C/G) [the authors counted from the beginning of their sequences that start with CTTGGTCA]. It must be noted that those critical positions which they listed for the ITS bear a few errors: positions **460**, 461, 463 should read 460, 462, **463** (bold = gap, position 460 corresponds to pos. 77 in Figures 23–24), and 607 is an error for 507 (=pos. 124 in Figures 23–24). LSU sequences from *Hy. albidus* were not available in GenBank for comparison with *Hy. fraxineus*.

#### Type sequences of *Hymenoscyphus albidus*

Husson et al. (2011) succeeded to isolate ITS rDNA from the two ~160 years old duplicates of the type collection of *Hy. albidus* preserved in the Caen Herbarium. According to their sequences deposited in GenBank, only 67 nucleotides (part of ITS1) were gained from n° 1604 (HM193465), but 510 nucleotides (part of SSU, ITS1, and 5.8S) from n° 2004 (HM193466). Both sequences fully match previously obtained sequences of *Hy. albidus* in GenBank, thus confirming the identity of the type material. Five critical positions are located in the region of the 67 nucleotides of n° 1604, (77, 79, 80, 87, 101; Tables 5 and 6), allowing exclusion of *Hy. fraxineus*, while the sequence from n° 2004 covers all 7 critical positions of the ITS1.

**Table 5.** Part of ITS1 which includes seven critical positions. At two of them (marked in blue: 80, 124) European *Hy. fraxineus* deviates from NE-Asian *Hy. fraxineus*. Positions in red (77, 79, 87, 101, 117) concern definition positions against *Hy. albidus* that apply also for Asian samples (see also Table 6).

**Table 6.** Variable nucleotide positions of the ITS1 (77–155), 5.8S (212), and ITS2 (355–453) of *Hymenoscyphus albidus* and *Hy. fraxineus* from European and Asian samples [position numbers starting with the last five nucleotides of the SSU: ‘CATTA’]; ***/** = normal case, * = infrequent abnormal case. Definition positions that apply also to Asian samples are marked in red, whereas at the blue markings Asian and European samples of *Hy. fraxineus* differ, the former matching *Hy. albidus*. Positions identical to all sequences are omitted, and identical sequences are equally omitted except for a few from Asia.

#### Intraspecific variation in the ITS

Within the many European sequences of *Hy. fraxineus* variation in the ITS was noted only in on sample (positions 124 and 379, see Table 6). In *Hy. albidus* three sequences showed variation, which concerns positions 86, 93, and 399. Among the 7 Japanese sequences variation occurred at positions 155, 212, and 443, and among the 6 Chinese sequences only at position 443 (Zheng & Zhuang 2014). The four Korean sequences (KF830850, KF830851, KF830852, KF830853) do not show any variation (J.G. Han personal communication). This infraspecific variation of *Hy. albidus* and *Hy. fraxineus* lies in the range of 0–0.5% of the entire ITS1-5.8S-ITS2 region, and the distance between the two species is about 2.2–2.4%.

#### Does European *Hymenoscyphus fraxineus* originate from the northeast of Asia?

The phylogenetic analyses by Zhao et al. (2013), J.G. Han (personal communication) and Zheng and Zhuang (2014) have shown that European and Asian *Hy. fraxineus* cluster together in a clade with rather high conformity and support, sharply separate from *Hy. albidus*. However, two deviations were noted, which question the asserted invasion of the pathogen from the presently known distribution area in Eastern Asia. According to the results presented by Zhao et al., all seven Japanese ITS sequences fully concur with European *Hy. fraxineus*, except for two nucleotide positions (80 and 124) at which the Japanese ones show the character of *Hy. albidus* (80: G instead of a gap, 124: C instead of T; counted when starting with CATTA, see Tables 5–6). The very same peculiarity is shown by all four ITS sequences from South Korea (J.G. Han) and all six from the northeast of China (Zheng & Zhuang 2014). As mentioned above, a sample of *Hy. fraxineus* from Czechia which was twice sequenced (FJ429386, GU586921) shows at position 124 the character of the Asian samples, while at position 80 it shows the typical gap of European *Hy. fraxineus*. This sequence further deviates from all sequences of *Hy. albidus*/*Hy. fraxineus* at position 379: T vs. C, Table 6).

In the two available sequences of LSU, European *Hy. fraxineus* (HM145907, Slovenia) deviates from Japanese (NBRC102368) at two positions in the overlapping part. More sequences are needed to clarify whether these two positions are constant markers. In the CAL and EF1-α gene regions, Japanese and European *Hy. fraxineus* isolates do not consistently deviate from each other, with at best one exception: at position 266 of CAL, Japanese isolates have A and European G, but one Japanese (TNS-F-12503, AB705208) has also G.

Further arguments against an origin from the northeast of Asia were presented by Drenkhan et al. (2014) who showed that the outbreak of the disease in NW-Estonia in 1995 was not associated with the areas where Mandshurian ash had repeatedly been introduced as seeds or seedlings since nearly 150 years.

#### Variation of Asian *Hymenoscyphus fraxineus* towards the genome of *Hymenoscyphus albidus*

Various nucleotide positions are presently known at which Asian *Hy. fraxineus* shows infraspecific variation. These can be divided into two groups: those positions at which *Hy. fraxineus* does not differ within Europe from *Hy. albidus*, and those at which the two differ. In the latter group (the definition positions), the Asian sequences show either the character of *Hy. fraxineus* or that of *Hy. albidus*, but never a further possibility. In other words: when deviating from European *Hy. fraxineus*, variation of Asian *Hy. fraxineus* was always towards the character of *Hy. albidus*. This strange phenomenon, which reminds of the two consistent deviations in the ITS between European and Asian *Hy. fraxineus*, might indicate a lineage from the ancient Asian *Hy. fraxineus* towards the younger *Hy. albidus.*

Variation in the ITS region within Asian *Hy. fraxineus* is noted at three positions (155, 212, 443) which are located outside the definition positions. Variation in the EF1-α gene within Japanese isolates concerns various positions. Among these are 11 definition positions, and all of them show either the character of European *Hy. fraxineus* or that of *Hy. albidus* (see Zhao et al. 2013, table 3; gaps not counted, pos. 236 must show a T instead of a gap). Also one position in the CAL gene (244) shows this phenomenon in both Japanese and Chinese specimens, and a further position (49) varies in one Chinese specimen towards *Hy. albidus*. These variations concern not only transitions but also transversions.

#### Phylogenetic considerations within the *Fraxinus*-inhabiting species

According to fossil finds, the genus *Fraxinus* occurred for the first time in the early Eocene(?) in North America. The land bridge between North America and Asia during this epoch allowed it to spread westwards while forming different species. According to molecular data, Asian species such as *F. mandshurica* are older than European species such as *F. excelsior* and *F. angustifolia* (Wallander 2008; Hinsinger 2010). Parallel to this evolutionary progress, different leaf decaying fungi developed on different *Fraxinus* species, such as *R. longipes* in North America (see below), *Hymenoscyphus fraxineus* in Eastern Asia, and *Hymenoscyphus albidus* in Europe.

The poor genetic variability of both *Hy. albidus* and *Hy. fraxineus* in comparison with a rather high variability of Eastern Asian *Hy. fraxineus* as detected by Zhao et al. (2013) and Gross, Hosoya et al. (2014) might suggest that not only the invasion of *Hy. fraxineus* but also that of *Hy. albidus* to Europe started from a small part of the whole Asian gene pool of *Hy. fraxineus*. This hypothesis is supported by the striking coincidence in the variable nucleotides of Japanese *Hy. fraxineus* with the stable nucleotides of European *Hy. albidus* (Zhao et al. 2013). Possibly, the ancient invasion of Europe derived from a single infection event, which resulted in the homothallic European species *Hy. albidus*. The loss of an anamorph as well as croziers at the ascogenous hyphae appears to be a consequence of this homothally. If these presumptions were true, the ancient invasion from Asia was perhaps also initially accompanied by ill effects to European ash.

#### Relationship of *Hymenoscyphus aesculi* and *Hymenoscyphus vacini*

In a phylogenetic study of the ITS rDNA of various species of *Hymenoscyphus* by V. Queloz and A. Gross (personal communication), two sequences of *Hy. aesculi* (H.B. 5736, 8914) and two of *Hy. vacini* (H.B. 8296, 9590) were gained from the dry apothecia. Unexpectedly, the two species did not cluster in the vicinity of *Hy. fraxineus* and *Hy. albidus*, but in a clade with two other foliicolous species, *Hy. microserotinus* (HMAS 68521) and ‘*Hy. serotinus*’ (HMAS 82122, = *Hy.* aff. *vacini*, see Baral and Bemmann 2013).

A pairwise distance analysis of the complete ITS1-5.8S-ITS2 region showed a difference between *Hy. aesculi*/*Hy. vacini* and *Hy. albidus*/*Hy. fraxineus* of 11–12.5%. The two *Hy. aesculi* sequences vary by 0.2% (1 nucleotide and 4 gaps in the ITS2), while the two *Hy. vacini* sequences are virtually identical and show a distance of 4.7–5% to *Hy. aesculi. Hy. microserotinus* deviates from *Hy. aesculi* by 4.5% and from *Hy. vacini* by 3.5%, and *Hy.* aff. *vacini* by 9–11% from the remaining three species of this clade.

### Generic concept of *Hymenoscyphus* and taxonomic weight of the dark stroma

*Hy. albidus* is currently placed in *Hymenoscyphus*, the type genus of the family Helotiaceae. From its earlier placement in *Helotium* it was transferred merely for nomenclatural reasons. The concept of *Hymenoscyphus* as applied in later years was heterogeneous, however, and included species showing affinities to the Hyaloscyphaceae, such as *Ca. herbarum* (Pers.) Gray (see Baral and Krieglsteiner 1985, p. 119; Baral 1994). On the other hand, species that obviously belong in Hymenoscyphus, including most of the here treated species, have been misplaced in rutstroemiaceous genera.

Karsten (1871) considered *He. albidum* as a variety of *He. scutula* (Pers.) W. Phillips. Both taxa undoubtedly belong in the core of *Hymenoscyphus*, with the type species *Hy. fructigenus* (Bull. ex Mérat) Gray, characterized by scutuloid ascospores. In a wider concept of the genus, an apical ring of the *Hymenoscyphus*-type is characteristic. This generic concept of *Hymenoscyphus* includes taxa with and without a dark substratal stroma.

In strong contrast to this concept, Carpenter (1981) argued with ‘the presence of a well-developed substratal stroma at the base of the apothecia, and an outer ectal excipulum of a textura prismatica’, the combination of which he believed to be diagnostic of the genus *Lanzia* (Sclerotiniaceae), to which he consequently combined *Hy. albidus*. Without stating arguments, Korf (1982) transferred the species to *Lambertella* in the same family (both genera are today classified in the Rutstroemiaceae). Like Carpenter, Korf (personal communication) was convinced of the high taxonomical importance of the substratal stroma within this family, but he preferred for *Hy. albidus* the genus *Lambertella* because he saw some brown spores (probably in European material which he collected in 1978). However, this concept neglects the characteristic scutuloid spore shape as well as the type of ascus apical ring, which is very distinctive between members of Helotiaceae and Sclerotiniaceae.

Svrček (1985) followed this concept when transferring *Helotium aesculi* and *He. vacini* to *Lanzia*, but it remains a mystery why he did not perform this transfer with *Hy. albidus*. Also Hosoya et al. (1993) fully accepted Carpenter’s view but preferred for *H. albidus* the genus *Lambertella* because of the drastic colour change of the overmature spores towards brown, which he observed in Japanese material now identified as *H. fraxineus*.

Baral (in Baral & Krieglsteiner 1985, p. 121) and Spooner (1987, p. 199) expressed their doubts about the taxonomic value of the stroma at the family level, being aware of the morphological similarity in ascospore shape and excipular structure of, e.g., *Hymenoscyphus albidus* and *Lan. vacini* with species of *Hymenoscyphus* which do not darken the substrate, such as *He. scutula*.

Zhang and Zhuang (2004) and Zhuang and Liu (2007) found by molecular methods that previous concepts of *Lanzia* need reconsideration, and that species with scutuloid spores, such as *Hy. serotinus* and *Hy. microserotinus*, belong in *Hymenoscyphus*, despite their dark substratal stroma. The placement of *Hy. albidus* and *Hy. fraxineus* in *Hymenoscyphus* is confirmed by molecular-phylogenetic analyses in Zhao et al. (2013) and Zheng and Zhuang (2014), also by two unpublished molecular studies (V. Queloz & O. Holdenrieder and J.G. Han & H.D. Shin, personal communication). The undoubtedly closely related *Hy. aesculi* and *Hy. vacini* are similarly misplaced in *Lanzia*, but molecular data were only available for *Hy. aesculi* which nested within *Hymenoscyphus* (V. Queloz personal communication).

*Lambertella torquata* Zhuang (1995), on herbaceous stems from Anhui Province in China, has likewise scutuloid spores and a conical ascus apex (the shape of the apical ring is not described); therefore, it certainly represents a *Hymenoscyphus* as well. The described infundibuliform collar at the spore base undoubtedly concerns a modification of the setulae which are characteristic of quite a few species of *Hymenoscyphus*. Due to its very large spores *Lam. torquata* obviously represents a distinct species, which is here transferred as follows:

#### *Hymenoscyphus torquatus* (W.Y. Zhuang) I. Kušan & Baral, **comb. nov.** – MycoBank MB 809092

Basionym: *Lambertella torquata* W.Y. Zhuang, Mycotaxon 56, p. 41 (Zhuang 1995)

Also *Lambertella caudatoides* Zhuang and *Lambertella tengii* Zhuang have scutuloid spores and were described in *Lambertella* because the spores turn light brown when they become median septate (Zhuang 1999, 2002). Undoubtedly they belong in *Hymenoscyphus*, but their transfer should await careful comparison and redescription, e.g., concerning the ascus base.

A further misplacement of stroma-forming discomycetes in the Rutstroemiaceae concerns *Dicephalospora* Spooner (1987, on twigs) and the two petiole-inhabiting species *Lanzia huangshanica* Zhuang (1995) and *Lanzia aurantiaca* (Zhuang) Zhuang in Zhuang and Liu (2007). A collection of *D. rufocornea* (Berk. & Broome) Spooner from Mauritius was studied (H.B. 6962) and found to have an amyloid apical ring of the *Hymenoscyphus*-type. The spores are not scutuloid, though slightly heteropolar, and possess round gelatinous appendages at both ends, typical of *Dicephalospora. Lan. huangshanica* has similar spores but without appendages, but the type of amyloid ring is not discernible on the photos in Zhuang (1995). A pale yellow granular exudate releases a honey-yellow pigment in KOH in *Dicephalospora*, whereas no such pigment is released in *Lan. huangshanica*. In a phylogenetic study by Zhuang and Liu (2007), the two taxa cluster in one clade, quite separate from the true Rutstroemiaceae, but also remote from *Hymenoscyphus*. Possibly *Lan. huangshanica* will be transferred to *Dicephalospora* in the future, a view also shared by Zhuang and Liu (2007).

#### *Rutstroemia longipes*, a similar though unrelated species on *Fraxinus* in North America

Records of *Hymenoscyphus albidus* or *Hy. fraxineus* on the American continent appear to be so far unknown. However, an equivalent leaf decaying fungus on *Fraxinus* with striking similarities exists: *R. longipes*. According to White (1941, p. 203, 204), this very common species fruits in late summer (July–Sept.) on entirely blackened rachises of *Fraxinus americana* and *F. nigra* in eastern and central areas of North America.

This species was also reported from Europe; however, a collection under this name on *Fraxinus angustifolia* from Hungary (leg. R.P. Korf & P. Lizoň, det. R.P. Korf, PRM 818046), made during the VII. Congress of European Mycologists in Budapest in Sept. 1978, was briefly mentioned by Svrček (1979, p. 155). A further collection made during this meeting is deposited in CUP and was examined by the first author: Kiskunság National Park, Bugac, Hungary, on blackened rachises of *Fraxinus angustifolia* ssp. *pannonica*, 20.IX.1978, R.P. Korf, H. Dissing et al. (CUP-059489, H.B. 9860).

During the past hundred years the species has been placed in various genera. White (1941) lists the following synonyms:

***Rutstroemia longipes*** (Cooke & Peck) White, Lloydia 4, p. 203 (1941)
≡ *Peziza longipes* Cooke & Peck, Bull. Buffalo Soc. nat. Sci. 1, p. 295 (1875)
≡ *Phialea longipes* (Cooke & Peck) Sacc., Syll. fung. (Abellini) 8, p. 267 (1889)
≡ *Lanzia longipes* (Cooke & Peck) Dumont & Korf, Mycotaxon 7(2), p. 185 (1978)
≡ *Hymenoscyphus longipes* (Cooke & Peck) Kuntze, Revis. gen. pl. (Leipzig) 3(2), p. 485 (1898)
= *Helotium sulfurellum* Ellis & Everh., Bull. Torrey bot. Club 10(7), p. 98 (1883)
≡ *Ciboria sulfurella* (Ellis & Everh.) Rehm, in Durand, Bull. Torrey bot. Club 29, p. 461 (1902)
≡ *Calycina sulfurella* (Ellis & Everh.) Kuntze, Revis. gen. pl. (Leipzig) 3(2), p. 449 (1898)
= *Ciboria tabacina* Ellis & Holw., Bull. Geol. Nat. Hist. Surv. Minnesota 3, p. 35 (1887)

White (1941) emphasized the ‘more prominently developed stroma than any other member of the genus’ [*Rutstroemia*]. His precise description of the stroma in section being ‘seated directly on the sclerenchyma’ matches exactly the situation in *Hy. albidus* and *Hy. fraxineus*. However, an acervular conidial state is formed ‘more or less associated with the black stromatic lines’, with conidiophores and conidia not unlike *Ch. fraxinea*. Comparable ellipsoid conidia are formed from the germinating ascospores.

The thin apothecial stipe of a very variable length reminds indeed of a Rutstroemiaceae, and the entirely yellow apothecia resemble those of *R. luteovirescens* (Roberge ex Desm.) W.L. White, a species currently recorded on petioles of *Acer* but originally described from *Tilia* and *Platanus*. Seaver (1951) even synonymized *R. longipes* with *R. luteovirescens*. The morphological differences between the two species are actually not very striking, and a genetical comparison has apparently never been done. White (1941) distinguished them by in the fresh state sulphur-yellow vs. greenish-yellow apothecia, which on drying turn dark brown in *R. longipes* while remaining more or less unchanged in *R. luteovirescens*, also by slightly larger asci and spores in the latter species.

The oblong-ellipsoid, slightly inequilateral ascospores were reported by White as (9–)10–14(–16) × 4.5–6 µm and to contain two large oil drops when fresh. In the specimen from Hungary their size is given by Svrček (1979) as 10–11.5 × 4.5–5 µm, which is here confirmed (†9.8–11.5 × 4.3–5 µm). The overmature spores are 1–3-septate. The production of ellipsoid conidia on short pegs or germ tubes appears to be rather untypical for a member of *Rutstroemia*, in which usually globose to subglobose conidia are formed (see also Whetzel 1945, as ‘spermatia’, these conidia are also typical of other genera of Sclerotiniaceae). However, for *Torrendiella ciliata* Graddon (1979) illustrated narrowly tear-shaped conidia. Acervuli in which similar elongate-ellipsoid conidia are formed were reported by White also for *R. longiasca* (Cavara) White, *R. renispora* (Ellis) White, and *R. pruni-serotinae* Whetzel & White.

Despite White’s detailed descriptions, those of the asci are very brief and in some species he does not even state the iodine reaction. Also information about the ascus base is lacking, contrary to his later studies. His drawings are not exact enough to recognise the type of apical ring: even White’s illustrations of typical members of *Rutstroemia* mislead to think that they have an ascus apex of the *Hymenoscyphus*-type. The present re-examination of *R. longipes* shows that the amyloid apical ring is of the *Sclerotinia*-type. Also a specimen of *R. pruni-serotinae* (New York, Ithaca, Lloyd Preserve, McLean, leaves of *Prunus spinosa*, 5.VIII.1932, H.H. Wetzel, CUP 025545, H.B. 9863) was re-examined and confirmed to be sclerotiniaceous. In both specimens the asci arise from croziers.
